# Discovery of benzamide-hydroxypyridinone hybrids as potent multi-targeting agents for the treatment of Alzheimer's disease

**DOI:** 10.1080/14756366.2021.1978081

**Published:** 2021-10-04

**Authors:** Xiaoying Jiang, Jianan Guo, Changjun Zhang, Jinping Gu, Tao Zhou, Renren Bai, Yuanyuan Xie

**Affiliations:** a College of Pharmaceutical Science, Collaborative Innovation Centre of Yangtze River Delta Region Green Pharmaceuticals, Zhejiang University of Technology, Hangzhou, China; b College of Material, Chemistry and Chemical Engineering, Key Laboratory of Organosilicon Chemistry and Material Technology, Ministry of Education, Hangzhou Normal University, Hangzhou, China; c College of Chemistry and Chemical Engineering, Central South University, Changsha, China; d School of Food Science and Biotechnology, Zhejiang Gongshang University, Hangzhou, China; e School of Pharmacy, Hangzhou Normal University, Hangzhou, China; f Key Laboratory of Elemene Class Anti-Cancer Chinese Medicines, Engineering Laboratory of Development and Application of Traditional Chinese Medicines, Collaborative Innovation Center of Traditional Chinese Medicines of Zhejiang Province, Hangzhou Normal University, Hangzhou, China

**Keywords:** Alzheimer’s disease, multifunctional candidate, benzamide-hydroxypyridinones, iron chelators, MAO-B inhibitors

## Abstract

A novel class of benzamide-hydroxypyridinone (HPO) derivatives were innovatively designed, synthesised, and biologically evaluated as potential multitargeting candidates for the treatment of Alzheimer's disease (AD) through pharmacophores-merged approaches based on lead compounds **18d**, benzyloxy phenyl analogs, and deferiprone (DFP). These hybrids possessed potent Monoamine oxidase B (MAO-B) inhibition as well as excellent iron chelation, with pFe^3+^ values ranging from 18.13 to 19.39. Among all the compounds, **8g** exhibited the most potent selective MAO-B inhibitor (IC_50_ = 68.4 nM, SI = 213). Moreover, **8g** showed favourable pharmacokinetic properties and had great potential to penetrate the BBB *in silico* and PAMPA-BBB assay. Molecular modelling showed that **8g** could adopt an extended conformation and have more enhanced interactions with MAO-B than **18d**. *In vitro* and *in vivo* assays demonstrated that **8g** remarkably resisted Aβ-induced oxidation and ameliorated cognitive impairment induced by scopolamine. Taken collectively, these results suggest that compound **8g** is a potential multifunctional candidate for anti-AD treatment.

## 1. Introduction

With the development of society and the improvement of living standards, human life expectancy has been extended and population ageing has swept the world. The prevalence of dementia is increased with ageing and longevity, especially Alzheimer’s disease (AD). AD is an irrevocable progressive neurodegenerative disorder characterised by a progressive deterioration in memory, incoherent language, cognitive impairments, and behavioural abnormalities^1^. In recent years, it has affected about 50 million people worldwide and it is expected to increase four times by 2050^2^. The incidence rate will also continue to rise, placing a heavy burden on families and societies. Therefore, AD has become a major socio-economic and healthcare concern which has led to an urgent need to develop novel and more efficient anti-AD drugs.

The pathogenesis of AD is still enigmatic and complicated. Many factors, such as loss of acetylcholine (ACh)^3^^,^^4^, aggregation of Aβ^5^, hyperphosphorylation of tau protein^6^, disturbance of biometallic homeostasis^7^, oxidative stress^8^, neuroinflammation, and activation of microglia cells^9^, are all considered to play a pivotal role in the pathogenesis of AD and possess complicated interconnections. The recognised multifactorial nature of AD and its consequent complexity is thought to account for the absence of effective drugs based on a single target. Therefore, the multitarget-directed ligands (MTDLs) can simultaneously intervene in more than two AD pathogenesis and may achieve better therapeutic outcomes when the mechanisms of action are complimentary^10^^,^^11^.

An elevated level of iron has been demonstrated to be associated with a variety of pathogenesis of AD. The higher iron levels in AD patients will stimulate the expression of amyloid protein precursor (APP) gene and tan protein, which leads to binding to Aβ and tau protein, further promoting Aβ aggregation and tau hyperphosphorylation^12^. The excess iron ions can also activate microglia cells to produce reactive oxygen species (ROS), causing mitochondrial dysfunction, oxidative stress, and neuronal death^13^. In addition, the hydrogen peroxide produced by the oxidation of neurotransmitters was catalysed by MAO-B, which will further participate in the free radical reaction catalysed by iron and then aggravate oxidative stress^14^^,^^15^. Therefore, we believed that combining two major functions (MAO-B inhibition and metal chelation) into a single molecule may afford a promising multifunctional therapeutic strategy for AD therapy (metal chelation, MAO-B inhibition, Aβ aggregation inhibition, and antioxidant activity).

Deferiprone (DFP), a typical orally active hydroxypyridinone (HPO) iron chelator, has been widely used clinically for the treatment of thalassaemia^16^. It has also been involved in many clinical trials to treat AD and Parkinson’s disease (PD) because of their ability to remove excess iron from the brain^17^^,^^18^. HPOs have high selectivity and affinity for iron which can form steady neutral 3:1 iron complexes at physiological pH, enabling these complexes to easily penetrate cell membranes through simple diffusion and facilitate iron removal from iron-overload cells^19^^,^^20^. Many studies have reported HPO derivatives as MTDLs with potential efficacy in the treatment of AD^21–24^.

In our previous work, some coumarin-HPO derivatives were designed and biologically evaluated as multitargeted iron chelators^25–27^. As a continuation of this research, we present the design, synthesis, and biological evaluation of a class of novel benzamide-HPO derivatives as multitargeting iron chelators with potent anti-AD effects here.

**Figure 1.**
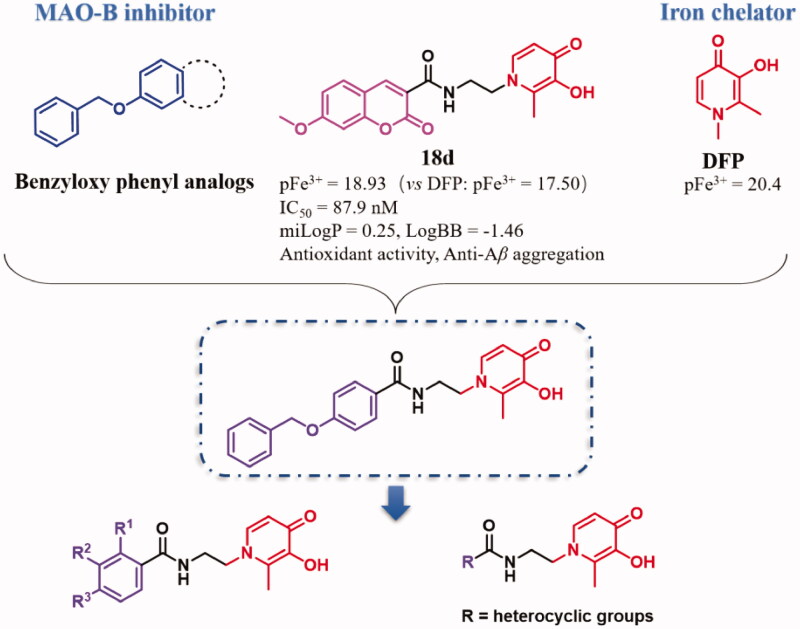
Rational design of benzamide-HPO hybrids as MTDLs.

The main rational design for these novel benzamide-HPO hybrids was through pharmacophores-merged approaches based on lead compound **18d**^27^, benzyloxy acetophenone, and DFP. According to our previous research, most of the coumarin-HPO hybrids possess certain MAO-B inhibitory activity and excellent iron-chelating activity^27^. The structure-activity relationship (SAR) shows that C-2 substituted HPOs and C-7 benzyloxy or alkoxyl substituted coumarins were optimal, and the amido bond enhanced the interaction with MAO-B. Compound **18d** was demonstrated to be a promising MTDL. However, these compounds have disadvantages of poor solubility, poor permeability, and relatively low Log *P* and Log BB values, which may be due to the lactone ring of coumarin. Moreover, benzyloxy phenyl and its analogs have been reported to possess preferable MAO-B inhibition with high selectivity and potent inhibition, such as safinamide^28–30^. Therefore, to improve the activities and physicochemical properties, the structure was simplified and optimised based on **18d**. A novel class of benzamide-HPO hybrids was innovatively designed and synthesised based on pharmacophores-merged approaches (Figure 1).

## 2. Results and discussion

### 2.1. Chemistry

**Scheme 1.**
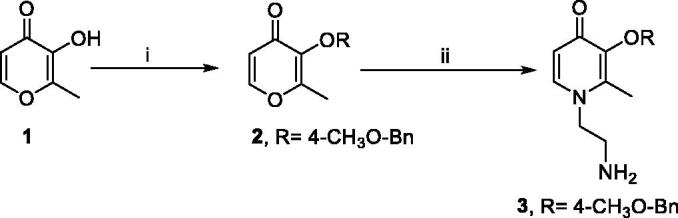
Synthetic route of compound **3**.^a a^Reaction conditions: (i) 4-methoxylbenzyl chloride, K2CO3, DMF, 80 °C, 2 h. (ii) Ethylenediamine, NaOH, ethanol: water = 1.1:1 (v/v), 70 °C, 1.5 h.

**Scheme 2.**
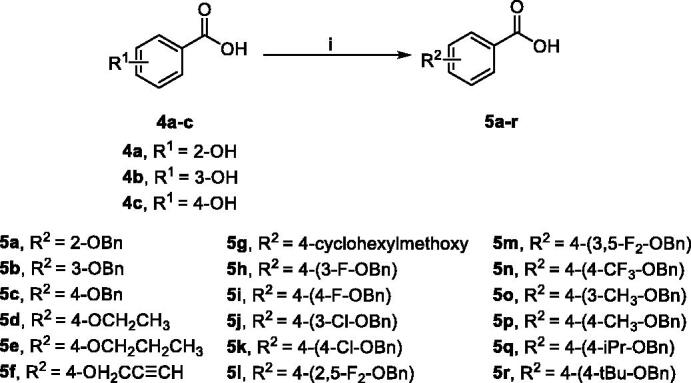
Synthetic route of compounds **5**.^a a^Reaction conditions: (i) alkyl or benzyl bromides, KOH, ethanol: water = 2:1 (*v*/*v*), reflux, 5–30 h.

**Scheme 3.**
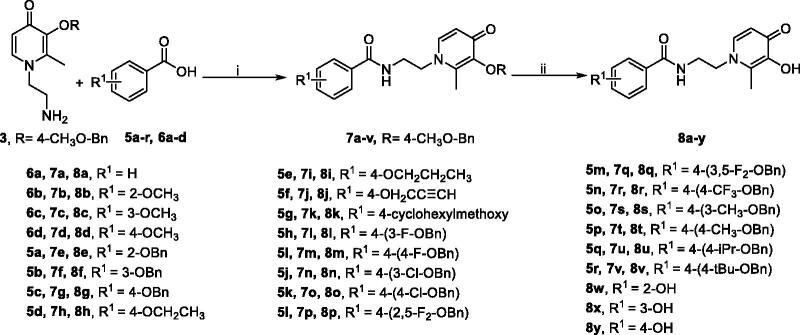
Synthetic route of compounds **8**.^a a^Reaction conditions: (i) (1) **5a-r/6a-d**, DCC, DMAP, 2-mercaptothiazoline, DCM, r.t., 24 h; (2) **3**, DCM, r.t., 24 h. (ii) BBr_3_ or BCl_3_, anhydrous DCM, −78 to −48 °C to r.t., 12 h.

**Scheme 4.**
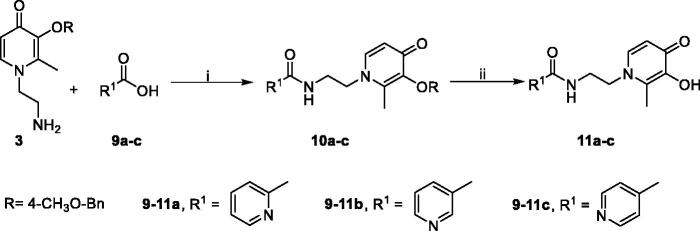
Synthetic route of compounds **11**.^a a^Reaction conditions: (i) (1) **9a–c**, DCC, DMAP, 2-mercaptothiazoline, DCM, r.t., 24 h; (2) **3**, DCM, r.t., 24 h. (ii) BCl_3_, anhydrous DCM, −48 °C to r.t., 12 h.

The synthetic strategies for the benzamide-HPO hybrids **8a–y** and **11a–c** are presented in Schemes 1–4. According to previous research, the biological activities of 2-methyl substituted HPOs are optimal. For the protective group of 3-hydroxyl on HPOs, 4-methoxybenzyl was more easily removed than the benzyl group^27^. Therefore, to reduce the difficulty of selective deprotection, 4-methoxylbenzyl chloride was used to protect the 3-hydroxyl group of commercially available maltol **1** (Scheme 1). Then compound **2** was reacted with ethylenediamine to produce intermediates **3** under alkaline conditions^27^.

The synthesis of alkoxy and benzyloxy substituted benzoic acids was shown in Scheme 2. The *O*-alkylated or *O*-benzylated products **5a–r** with moderate yields were obtained by the reaction of *o*-, *m*-, and *p*-hydroxybenzoic acids **4a–c** with benzyl bromides or alkyl bromides in ethanol/water with KOH^31^.

Subsequently, amide derivatives **7a–v** and **10a–c** were formed by activation of carboxyl groups of benzoic acids **5a–r/6a–d** and pyridinecarboxylic acids **9a–c**, which using dicyclohexylcarbodiimide (DCC), 2-mercaptothiazoline, and 4-(dimethylamino)pyridine (DMAP). Selective deprotection of **7a–v** and **10a–c** was achieved by appropriate equivalent BCl_3_, providing the designed compounds **8a–v** and **11a–c** as white solids in excellent yields. However, to obtain compounds **8w–y**, the methoxy groups on compound **7b–d** were removed by BBr_3_ (Schemes 3 and 4).

### 2.2. Iron-chelating activity test

**Scheme 5.**
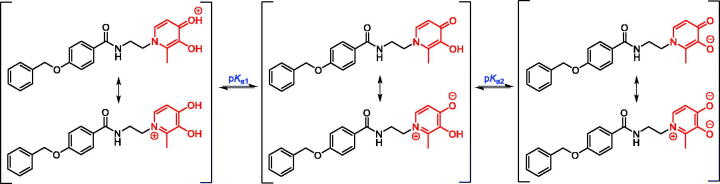
Resonance structures and proton equilibria of **8g**.

3-Hydroxypyridin-4-ones have high affinity and selectivity for Fe^3+^. Because of the competition effect in aqueous solutions at different pH values, the superior selectivity and affinity for Fe^3+^ derived from the extensive delocalisation of electrons in its resonance structures (Scheme 5)^20^. In biological conditions, the pFe^3+^ value is a more useful parameter than the traditional stability constant in assessing the affinity of ligands for Fe^3+^. It was defined as the negative logarithm of the concentration of free Fe^3+^ in solution at pH 7.4 ([Fe^3+^]_total_ = 10^−6 ^M, [ligand]_total_ = 10^−5 ^M)^20^. Therefore, the p*K*_a_ values of compounds and their affinity constants for Fe^3+^ were measured (Table 1)^32^.

**Table 1.** The p*K*_a_ values of all compounds and their affinity constants for Fe^3+^.^a^

Compound	p*K*_a1_	p*K*_a2_	Log *β*_1_^b^	Log *β*_2_^c^	Log *β*_3_^c^	pFe^3+^
**8a**	3.43	9.81	14.93	26.35	35.83	19.14
**8b**	3.41	9.88	14.64	26.20	35.45	18.56
**8c**	3.37	9.83	14.68	26.17	35.55	18.80
**8d**	3.34	9.82	14.52	25.95	35.25	18.53
**8e**	3.38	9.99	14.66	26.46	35.64	18.43
**8f**	3.38	9.92	14.75	26.85	35.33	18.38
**8g**	3.38	9.69	14.60	26.04	35.65	19.31
**8h**	3.27	9.80	14.67	26.06	35.33	18.67
**8i**	3.41	9.84	14.56	25.98	35.18	18.40
**8j**	3.41	9.88	14.67	26.14	35.24	18.35
**8k**	3.21	9.89	14.44	26.31	35.84	18.91
**8l**	3.51	9.74	14.76	26.72	35.87	19.39
**8m**	3.62	9.76	15.03	26.77	35.31	18.81
**8n**	3.32	9.55	14.64	25.95	34.39	18.51
**8o**	3.33	9.63	14.43	26.09	34.55	18.44
**8p**	3.27	9.82	14.47	26.58	35.44	18.74
**8q**	3.27	9.82	14.61	26.63	35.73	19.01
**8r**	3.25	9.78	14.48	26.01	34.98	18.39
**8s**	3.31	9.86	14.67	26.67	35.67	18.85
**8t**	3.12	9.93	14.45	26.42	35.19	18.18
**8u**	3.33	9.58	14.67	25.58	35.36	19.35
**8v**	3.29	9.56	14.69	25.51	35.20	19.25
**8w**	3.47	9.68	14.45	25.69	34.63	18.34
**8x**	3.28	9.75	14.67	25.93	34.96	18.46
**8y**	3.33	9.95	14.61	26.07	35.42	18.31
**11a**	3.35	9.86	14.52	25.92	35.10	18.27
**11b**	3.09	9.79	14.40	25.68	34.75	18.13
**11c**	3.15	9.81	14.74	26.21	35.58	18.89
DFP	3.64	9.79	14.75	26.04	34.84	18.24
DFP^a^	3.64	9.79	14.86	27.13	36.76	20.12
DFP^d^	3.61	9.78	15.03	27.42	37.35	20.74

^a^Measured in KCl (0.1 M).

^b^Measured in KCl (0.1 M): DMSO = 9:1 (*v*/*v*).

^c^Measured in KCl (0.1 M): DMSO = 3:2 (*v*/*v*).

^d^Measured in KCl (0.1 M) which was from reference^19^.

**Figure 2.**
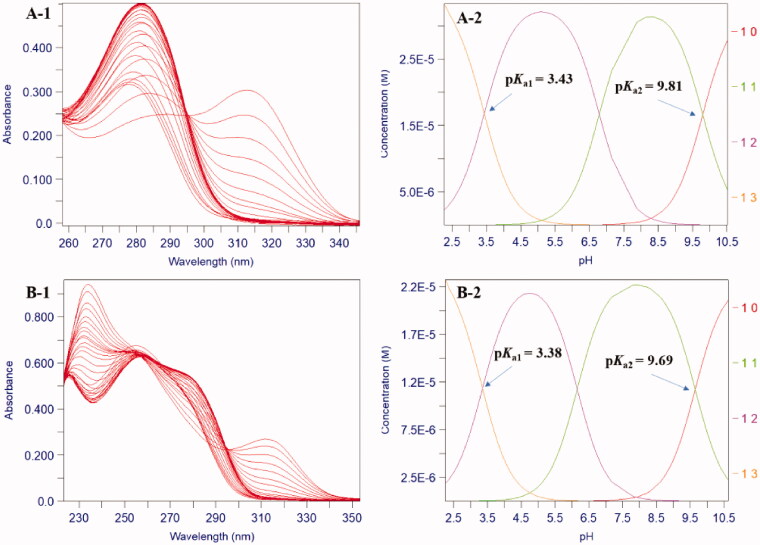
pH dependence of UV spectra of compounds **8a** and **8g** in 0.1 M KCl over the pH range 2.0–10.5 at 25 °C. (A-1/B-1**)** The 2D titration spectra of compound **8a/8g**. (A-2/B-2) Speciation plots of compound **8a/8g**.

Similar to the previous research, because of the amide bond, all compounds were fitting to three p*K*_a_ values by spectrophotometric and speciation plot analysis, such as compounds **8a** and **8g** (Figure 2)^27^. There was no doubt that the p*K*_a1_ (<3.40) was attributed to the protonation of the 4-carbonyl oxygen group, the p*K*_a2_ (9.55–9.99) was belonging to the dissociation of the 3-OH group. These compounds were all determined in 0.1 M KCl aqueous solution, indicating that they have good water solubility. The spectrophotometric titration yielded two main p*K*_a_ values for all compounds over the pH range 2.0–10.5, such as **8a** and **8g**, which are 3.43, 9.81 and 3.38, 9.69, respectively. It could be seen that the ionisation equilibrium of compounds is pH-dependent and they possess uncharged property in the pH range of 6.0–8.0 (Figure 2). Obviously, the p*K*_a1_ values of compounds were almost all lower than the corresponding value of DFP. This is because that the substitutional groups on 1-nitrogen affect the negative charge delocalisation of 4-carbonyl oxygen.

**Figure 3.**
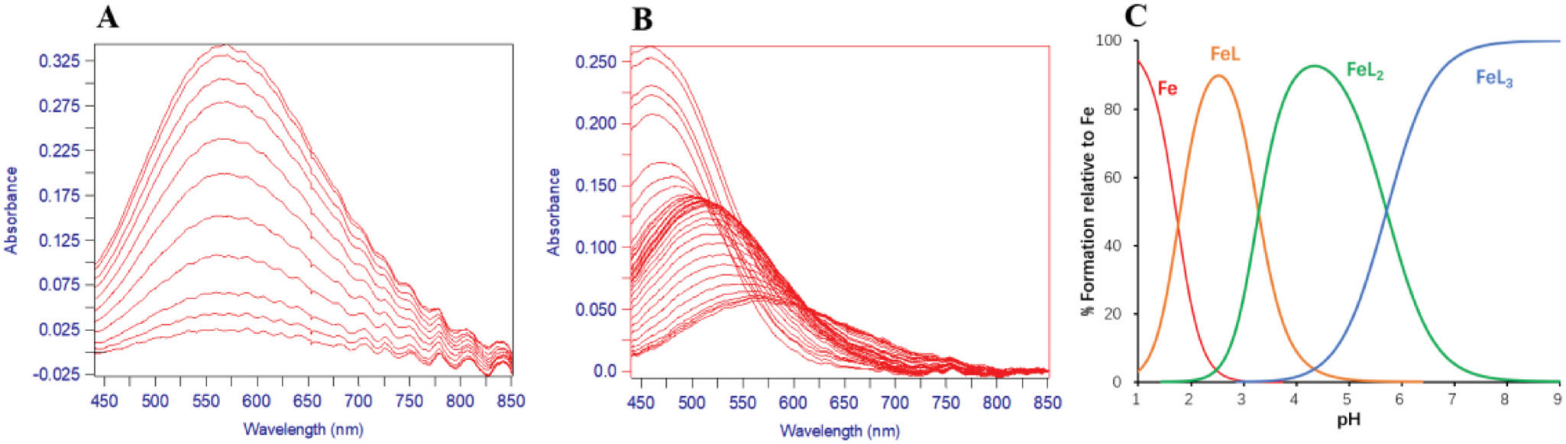
pH dependence of UV–Vis spectra of compound **8g** in 1.0 μM Fe^3+^ at 25 °C. (A) The spectra of compound **8g** (1.1 μM) over the pH range 1.113–2.183 in DMSO: 0.1 M KCl = 1:9 (*v*/*v*). (B) The spectra of compound **8g** (5.0 μM) over the pH range 2.534–9.069 in DMSO: 0.1 M KCl = 2:3 (*v*/*v*). (C) Speciation plot of Fe^3+^ and compound **8g**.

The affinity constants for Fe^3+^ (log *β*_1_, *β*_2_, and *β*_3_) were also analysed according to the absorption spectra of speciation between Fe^3+^ with ligands at different pH solutions. The pFe^3+^ values were then calculated based on the p*K*_a_ values and the above three affinity constants. All compounds exhibited excellent pFe^3+^ values (18.13–19.39) (Table 1). Compounds **8g**, **8l**, and **8u** exhibited the most potent iron chelation with pFe^3+^ values of 19.31, 19.39, and 19.35, respectively, which were higher than that of DFP (pFe^3+^ = 18.24) under the same experimental conditions. As found with the speciation plot of compound **8g**, the neutral 3:1 complexes dominated over the pH range 6–9 (Figure 3).

SAR showed that the 4-carbonyl and 3-hydroxyl groups on the HPO ring were necessary for iron chelation. The para- and meta- substitutions on the benzene ring showed better iron chelation than ortho-substitutions. Compounds with 4-bulky alkoxy, 4-benzyloxy, and 4-benzyloxy substituted by electron-withdrawing groups on the benzene ring exhibited good iron chelation. While the benzene ring replaced by pyridine ring exhibited relatively poor iron chelation. In general, all benzamide-HPO hybrids show favourable iron-chelating ability.

### 2.3. Human MAO-B inhibition assay

The MAO-B inhibitory ability of all benzamide-HPO hybrids was measured. As shown in Table 2, the inhibitory rate at the concentration of 1 μM and 100 nM were firstly screened using pargyline as the positive control. Most compounds displayed remarkable MAO-B inhibition with an inhibitory rate ranging from 60 to 80% at 1 μM. Compound **8l** showed more than 80% inhibitory effect but was still weaker than pargyline. When tested at 100 nM, most compounds exhibited MAO-B inhibitory rate between 40 and 50%. Five compounds (**8a**, **8g**, **8i**, **8l**, and **8m**) displayed inhibitory effects over 50%, which is superior to pargyline.

**Table 2.** MAO-B inhibitory rate of all compounds.

Compound	Inhibitory rate ± SEM (%, 1 μM)	Inhibitory rate ± SEM (%, 100 nM)	Compound	Inhibitory rate ± SEM (%,1 μM)	Inhibitory rate ± SEM (%, 100 nM)
**8a**	77.65 ± 0.60	57.78 ± 1.02	**8p**	65.11 ± 1.47	41.41 ± 2.45
**8b**	59.93 ± 1.57	38.78 ± 1.16	**8q**	63.89 ± 0.67	49.70 ± 4.22
**8c**	59.97 ± 0.14	31.91 ± 0.85	**8r**	63.44 ± 4.06	46.48 ± 6.99
**8d**	63.00 ± 3.90	36.02 ± 0.15	**8s**	71.33 ± 1.02	49.34 ± 2.58
**8e**	51.47 ± 1.06	33.08 ± 1.43	**8t**	64.10 ± 0.82	45.13 ± 1.62
**8f**	54.57 ± 0.51	37.93 ± 1.22	**8u**	62.92 ± 0.51	41.48 ± 1.94
**8g**	78.29 ± 0.16	50.92 ± 1.50	**8v**	64.25 ± 1.94	48.90 ± 1.93
**8h**	66.75 ± 0.34	48.07 ± 1.57	**8w**	56.43 ± 0.24	44.38 ± 0.23
**8i**	53.41 ± 0.31	50.34 ± 2.16	**8x**	55.75 ± 0.03	43.63 ± 0.34
**8j**	58.63 ± 1.18	38.71 ± 0.94	**8y**	55.28 ± 1.91	37.61 ± 1.01
**8k**	63.27 ± 0.92	39.49 ± 2.38	**11a**	61.48 ± 0.58	34.04 ± 5.19
**8l**	84.89 ± 3.51	51.43 ± 2.42	**11b**	60.84 ± 1.99	34.13 ± 1.58
**8m**	70.86 ± 2.51	50.44 ± 3.89	**11c**	69.71 ± 2.38	29.15 ± 2.14
**8n**	72.42 ± 3.26	47.66 ± 6.06	Pargyline	91.59 ± 0.09	50.30 ± 0.73
**8o**	63.61 ± 4.14	48.53 ± 4.37			

The IC_50_ values of 20 compounds with favourable MAO-B inhibition were subsequently measured (Tables 3 and 4). Most of the compounds exerted IC_50_ values between 100 and 200 nM. There was no doubt that five compounds (**8a**, **8g**, **8i**, **8l**, and **8m**) mentioned above also possessed much more potent MAO-B inhibition than pargyline, with IC_50_ values below 100 nM. Compound **8g** exhibited the highest inhibition and good selectivity for MAO-B (IC_50_ = 68.4 nM, SI = 213), which was demonstrated to be the most potent one. However, it is interesting that the compounds without benzyloxy phenyl motif (**8a**, **8i**) still showed available inhibition of MAO-B, with IC_50_ values very similar to that of compound **8g**, suggesting that benzyl may not be the key pharmacophore. This may provide useful guidance for us to design more concise and efficient compounds in the future.

**Table 3.** The IC_50_ values against MAO-B of the selected compounds.

Compound	IC_50_ (nM)	Compound	IC_50_ (nM)
**8a**	79.0 ± 0.30	**8q**	93.8 ± 6.48
**8d**	210.8 ± 1.21	**8r**	187.7 ± 0.42
**8g**	68.4 ± 6.05	**8s**	122.3 ± 0.21
**8h**	119.2 ± 2.76	**8t**	117.9 ± 4.24
**8i**	89.1 ± 10.09	**8u**	127.9 ± 3.96
**8k**	188.9 ± 7.51	**8v**	123.8 ± 1.32
**8l**	96.4 ± 6.78	**8w**	121.5 ± 0.86
**8m**	82.8 ± 0.80	**8x**	120.8 ± 1.94
**8n**	110.9 ± 1.10	**11b**	247.8 ± 9.56
**8o**	138.9 ± 1.74	Pargyline	107.3 ± 8.80
**8p**	174.6 ± 6.73		

**Table 4.** The IC_50_ values of compound **8g** against MAO-A and MAO-B.

Compound	IC_50_ ± SEM (nM)		SI^a^
	MAO-B	MAO-A	
**8g**	68.4 ± 6.05	14582 **±** 231.50	213
Pargyline	107.3 ± 8.80	4189 ± 5.00	39

^a^SI (selectivity index) = IC_50_ (MAO-A)/IC_50_ (MAO-B), which represents the selectivity for the MAO-B isoform.

The SAR study indicated that the para- and ortho-substitutions on the benzene ring exhibited better MAO-B inhibition than meta-substitutions. When substitutions were all on the para-benzene ring, compounds with saturated alkoxy with long chains, benzyloxy, and benzyloxy substituted by single electron-withdrawing groups at para-phenyl ring exhibited better inhibition effect on MAO-B. Moreover, poor MAO-B inhibition was obtained when the benzene ring was replaced by a pyridine ring.

### 2.4. Prediction of drug-like properties and BBB permeability

To further understand the drug-likeness, the molecular properties of these new hybrids were predicted and performed by molinspiration (http://www.molinspiration.com). It was found that miLog *p*-values of HPOs were closer to the experimentally measured values than those calculated by other programs^33^. All the compounds were in accordance with Lipinski’s rules and Veber’s rules. They also had appropriate topological polar surface area (TPSA) values except for **8a** because low TPSA (<75 Å^2^) may increase the risk of non-specific toxicity^34^. Subsequently, the BBB permeability is very critical for anti-AD compounds. Log *BB* was calculated with Clark’s equation while compounds with a Log *BB* value <−1.0 are not likely to enter the brain (Table S1)^35^. Therefore, 19 compounds (**8b**, **8e–8v**) possessed preferable drug-likeness with appropriate solubility and permeability. **8g** was found to be the optimum compound (miLog *P* = 1.87, Log BB = −0.77) when simultaneously possessed good iron chelation and MAO-B inhibition.

Another two *in silico* methods (ADMETlab and admetSAR) were also applied to predict the BBB permeability of compound **8g**^36^^,^^37^. This reliable classification model was built by machine learning ways and resampling methods^38^. As shown in Table 5, compound **8g** was classified as BBB + with a probability of 0.631 and 0.8164, respectively.

**Table 5.** The BBB permeability of compound **8g** is predicted by ADMETlab and admetSAR.

Property	Value	Probability
BBB^a^	Category 1	0.631
BBB^b^	Category +	0.8164

^a^Predicted by ADMETlab: Category 1: BBB+; Category 0: BBB−; BB ratio ≥ 0.1: BBB+; BB ratio < 0.1: BBB−.

^b^Predicted by admetSAR: Category −: BBB−, Category +: BBB+.

Certainly, the parallel artificial membrane permeation assay (PAMPA) was carried out to assess the capacity of **8g** to penetrate into the brain^39^. We have identified the effective permeability (*P*_e_) for seven commercial drugs with known CNS penetration as well as for the compound **8g** (Table 6). The standard concentration-absorbance curve for each compound was shown in the Supporting Information (Table S1). According to the BBB permeation limits defined by Di et al.^39^, compounds with *P_e_* > 4.0 were possessing high permeation, with *P_e_* < 2.0 were showing low permeation, and with 2.0 < *P_e_* < 4.0 were displaying uncertain permeability. Compound **8g** showed *P_e_* values above 4.0, suggesting that compound **8g** has a high potential to cross the BBB by passive diffusion.

**Table 6.** The permeability (*P*_e_, 10^–6 ^cm/s) results of compound **8g** and commercial drugs in the PAMPA-BBB assay and their prediction of CNS penetrability.

Compound	*P_e_* ± SEM^a^	*P*_e_	Prediction^d^
Donepezil	10.44 ± 0.98	7.3 ± 0.9^b^	CNS +
Testosterone	14.38 ± 1.53	17^c^	CNS +
Tacrine	4.12 ± 0.45	5.3 ± 0.19^b^	CNS +
Hydrocortisone	2.00 ± 0.04	1.9^c^	CNS ±
Piroxicam	2.05 ± 0.04	2.5^c^	CNS ±
Atenolol	0.45 ± 0.25	0.8^c^	CNS −
Theophylline	0.12 ± 0.01	1.07 ± 0.18^b^	CNS −
**8g**	4.07 ± 0.44		CNS +

^a^Data were from eight independent experiments.

^b^Data were from reference^39^.

^c^Data were from reference^40^.

^d^CNS +: *P_e_* (10^–6 ^cm/s) > 4.0, high BBB permeability; CNS ±: 2.0 < *P_e_* (10^–6 ^cm/s) < 4.0, uncertain BBB permeability; CNS −: *P_e_* (10^–6 ^cm/s) < 2.0, low BBB permeability.

### 2.5. Molecular modelling

**Figure 4.**
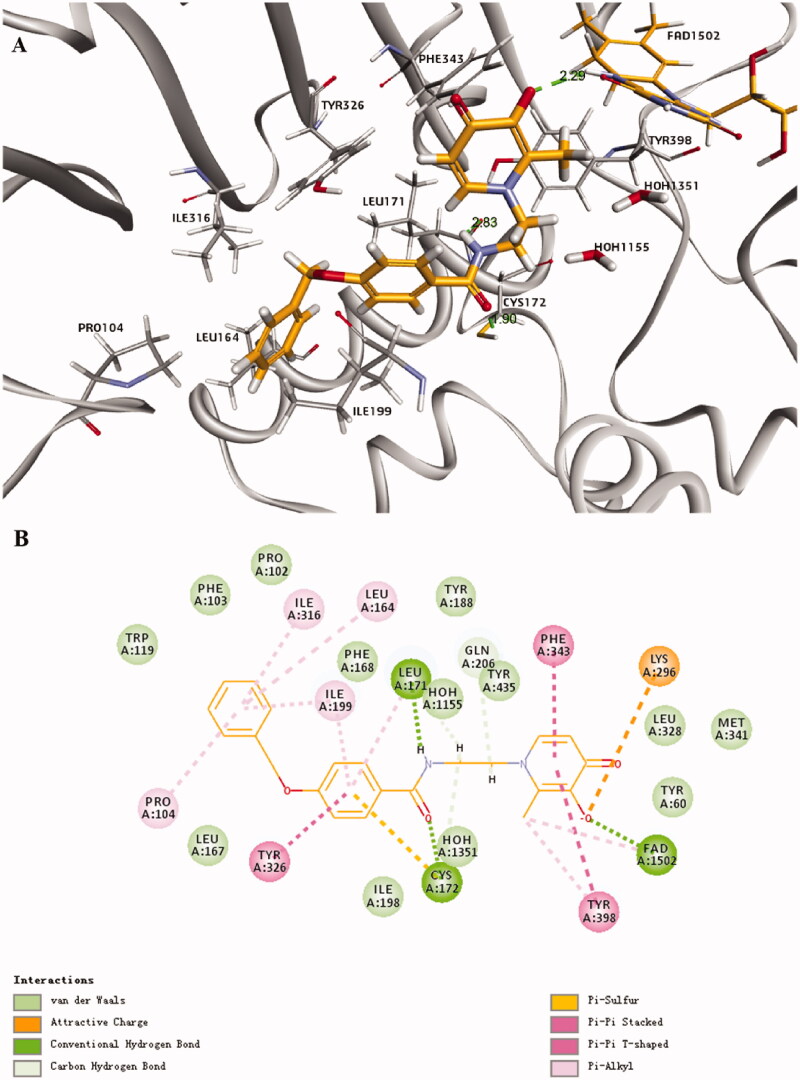
Best docking results for the compound **8g** with MAO-B (PDB entry 2V5Z) (compound **8g**, yellow sticks; FAD cofactor, orange sticks). (A) The 3D docking model. (B) The planar docking model.

The potential binding sites of optimum compound **8g** with MAO-B (PDB: 2V5Z) was shown in Figure 4, which was investigated by molecular docking. The ligand formed an extended conformation between the substrate and entrance cavities. The benzyloxyphenyl of **8g** has formed some lipophilic binding interactions with Tyr 326, Ile 316, Ile 199, Leu 164, Pro 104, Leu 171, and Cys 172 in the hydrophobic entrance cavity. The carbonyl oxygen of amide forms strong hydrogen bond interaction with Cys 172, NH forms strong hydrogen bond interaction with Leu 171, and also interacts with Gln 206 and Tyr 435 to some extent, indicating that the introduction of amide bond enhanced the interaction with enzyme and result in its good inhibitory activity against MAO-B. Furthermore, the hydroxyl of HPO forms Pi-sulfur interaction with Lys 296 and forms a hydrogen bond with the FAD cofactor. The methyl of HPO forms Pi-alkyl interaction with Tyr 398, which indicated that HPO also works as a critical segment for interacting with MAO-B. Therefore, all these interactions may explain the preferable activity of compound **8g**.

### 2.6. Antioxidant activity assays

**Figure 5.**
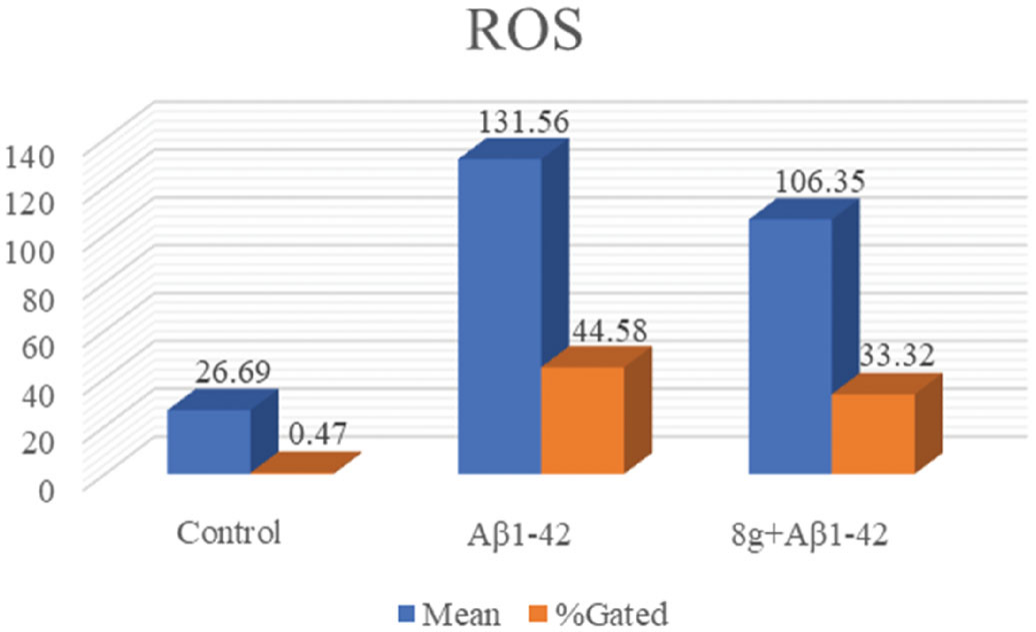
The antioxidant activity was measured by DCFH-DA. The concentration of **8g** and Aβ_1–42_ were 10 μM. “Mean” means average fluorescence intensity of all cells; “%Gated” means the percentage of positive cells in the total number of cells.

**Figure 6.**
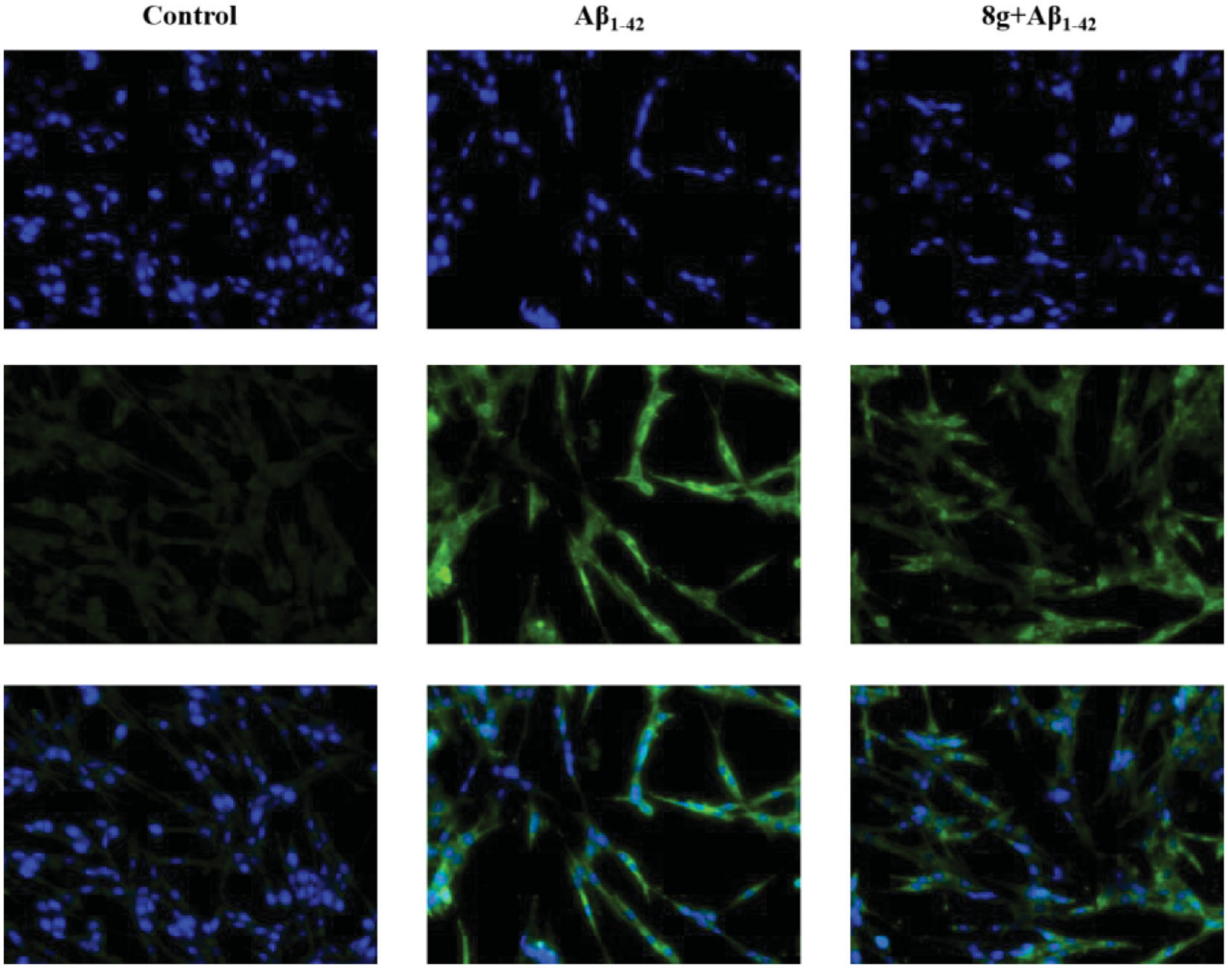
The intracellular ROS generation of compound **8g** (10 μM). Blue and green fluorescence represent the nucleus and cytoplasm, respectively.

Aβ deposition and peroxidation are also important clinical features of AD. Therefore, we subsequently assessed the antioxidation of compound **8g**, using 2′,7′-dichlorofluorescin diacetate (DCFH-DA) to detect intracellular ROS generation on the PC12 cell line derived from neural pheochromocytoma. Based on our previous research, the concentration of 10 μM of compound **8g** was chosen in this test. As shown in Figures 5 and 6, the fluorescence intensity of cells containing Aβ_1–42_ was much higher than normal cells, indicating that Aβ deposition would cause oxidative stress to a certain extent. When compound **8g** was adopted, it was surprisingly found that the Aβ_1–42_-induced intracellular ROS levels were significantly reduced (33.32% *vs*. model group 44.58%). The ROS fluorescence intensity also showed a significant decrease, indicating that **8g** had the ability to resist Aβ-induced oxidation.

### 2.7. Cognitive and memory assays *in vivo*

The anti-AD effect of compound **8g** on the scopolamine-induced cognitive dysfunction ICR mice model was assessed by Morris water maze (MWM) test^27^. Each group of mice was intraperitoneally injected with compound **8g** (15 mg/kg), pargyline (15 mg/kg), or PBS, and then injection with scopolamine (15 mg/kg) or PBS 30 min later once a day for 15 consecutive days. The MWM test was performed at the last 5 days including two days of memory and learning training, three days of cognitive assessment. On the fifth day, a probe training trial was performed and analysed. To make a contrast with lead compound **18d**, we still chose pargyline (15 mg/kg) as the positive drug. However, the dosage of scopolamine was increased from 5 mg/kg to 15 mg/kg to achieve a better modelling effect.

**Figure 7.**
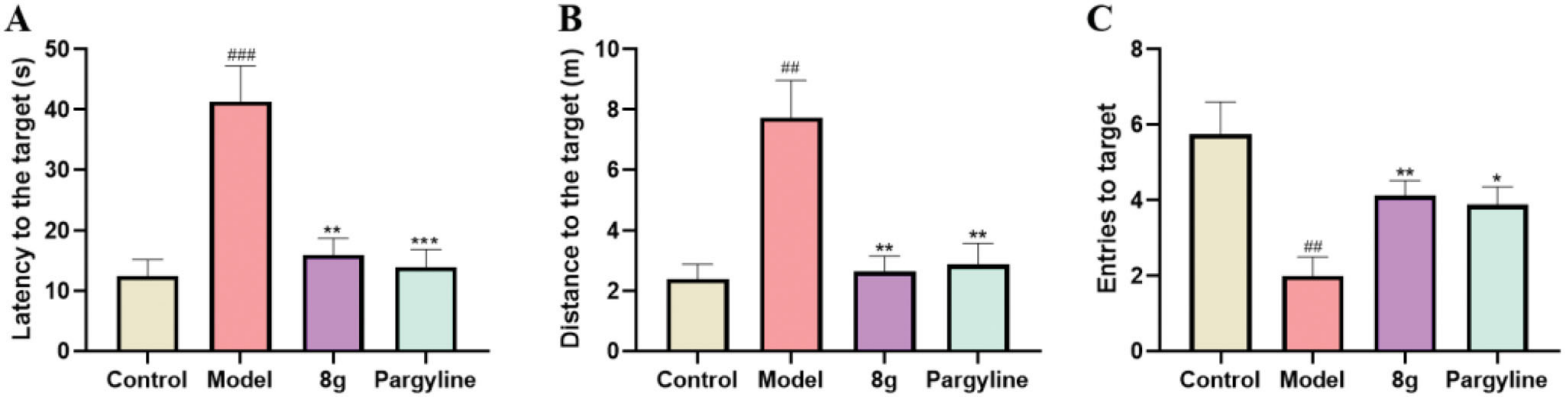
Effect of compound **8g** and pargyline on scopolamine-induced cognitive deficit ICR mice determined by MWM test. (A) Latency to the target. (B) Distance to the target. (C) Entries to the target.(*n* = 8, mean ± SEM; *p < 0.05, **p < 0.01, ***p < 0.001, **8g** or pargyline group *vs.* model group; ^##^*p* < 0.01, ^###^*p* < 0.001, Control group *vs.* model group).

**Figure 8.**
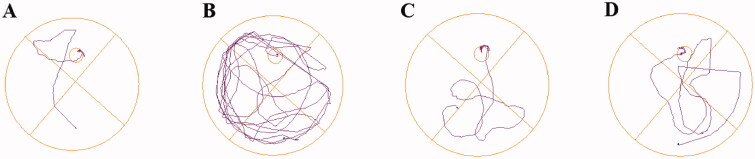
The representative trajectories in the MWM test. (A) Control. (B) Model. (C) Compound **8g**. (D) Pargyline.

The data for the last day was shown in Figure 7, the latency (12.54 ± 2.71 *vs.* 41.40 ± 5.87 s, ^###^*p* < 0.001) and the distance (2.40 ± 0.49 *vs.* 7.74 ± 1.23 m, ^##^*p* < 0.01) of mice treated with scopolamine were remarkably more prolonged than the control group. Moreover, the entries to the target were also significantly decreased (5.75 ± 0.86 *vs.* 2.00 ± 0.50, ^##^*p* < 0.01), demonstrating that the mice model of cognitive dysfunction has been well-established. Treatment with **8g** markedly reduced the latency (16.03 ± 2.76 s, **p < 0.01) and the distance to the target (2.64 ± 0.52 m, **p < 0.01), which a little better than the pargyline group (13.96 ± 2.94 s, ***p < 0.001) (2.89 ± 0.69 m, **p < 0.01). Compound **8g** (4.12 ± 0.40, **p < 0.01) also worked better than pargyline (3.85 ± 0.48, *p < 0.05) in increasing the entries to target. The representative trajectories (Figure 8) also showed that the model group was very lengthy and disorganised, while compound **8g** was clearer than pargyline, which demonstrated that the scopolamine-induced spatial learning and memory deficits were remarkably ameliorated by compound **8g**.

## 3. Conclusion

In conclusion, we reported a class of novel benzamide-HPO hybrids as potential anti-AD candidates with multiple biochemical properties based on the MTDLs strategy. All compounds possessed excellent iron chelation activity and showed promising MAO-B inhibition. Among them, compound **8g** was proved to be the most potent iron chelator (pFe^3+^ = 19.31) and the most effective selective MAO-B inhibitor (IC_50_ = 68.4 nM, SI = 213). *In silico* drug-likeness predictions and PAMPA-BBB assay demonstrated that **8g** possessed acceptable BBB permeability. Molecular modelling showed that **8g** could form an extended conformation and have more enhanced interactions with MAO-B than **18d**. *In vitro* assay indicated that compound **8g** significantly reduced the Aβ-induced intracellular ROS levels and remarkedly reversed the cognitive deficit in the MWM test. All results indicated that hybrid **8g** is an interesting and promising multifunctional agent with the potential to be a therapeutic candidate against AD.

## Supplementary Material

Supplemental Material
