# Supplementary material for: Discovery of benzamide-hydroxypyridinone hybrids as potent multi-targeting agents for the treatment of Alzheimer's disease
Source: J Enzyme Inhib Med Chem. 2021 Oct 4;36(1):2045–54. doi: 10.1080/14756366.2021.1978081 (PMC8510601; doi:10.1080/14756366.2021.1978081)
Supplement: Supplemental Material [file IENZ_A_1978081_SM2219.pdf]

Supporting Information

Discovery of benzamide-hydroxypyridinone hybrids as potent multi-targeting agents for the treatment of Alzheimer's disease

Xiaoying Jiang [a, b, c], Jianan Guo [a], Changjun Zhang [a], Jinping Gu [a], Tao Zhou [d], Renren Bai [e, f, *], Yuanyuan Xie [a, *]

[a] College of Pharmaceutical Science, Collaborative Innovation Centre of Yangtze River Delta Region Green Pharmaceuticals, Zhejiang University of Technology, Hangzhou, P.R. China.

[b] College of Material, Chemistry and Chemical Engineering, Key Laboratory of Organosilicon Chemistry and Material Technology, Ministry of Education, Hangzhou Normal University, Hangzhou, P.R. China.

[c] College of Chemistry and Chemical Engineering, Central south University, Changsha, P.R. China.

[d] School of Food Science and Biotechnology, Zhejiang Gongshang University, Hangzhou, P.R. China

[e] School of Pharmacy, Hangzhou Normal University, Hangzhou, P.R. China.

[f] Key Laboratory of Elemene Class Anti-Cancer Chinese Medicines; Engineering Laboratory of Development and Application of Traditional Chinese Medicines; Collaborative Innovation Center of Traditional Chinese Medicines of Zhejiang Province, Hangzhou Normal University, Hangzhou, P.R. China.

Contents

1. Supplementary Table
    1.1 Table S1. The ADMET evaluation of compounds
    1.2 Table S2. The standard concentration-absorbance curve for each compound
2. Chemistry
    2.1 Materials and instruments
    2.2 General synthetic procedure for **3**
    2.3 General synthetic procedures for **5a-r**
    2.4 General synthetic procedures for **7a-v** and **10a-c**
    2.5 General synthetic procedures for **8a-y** and **11a-c**
3. The determination of pK_a and iron (III) affinity
4. Calculation of pFe^{3+}
5. Human MAO inhibitory assay
6. PAMPA-BBB assay
7. Molecular modeling studies
8. Intracellular ROS detection
9. Cognitive and memory assays *in vivo*
10. ^{1}H and ^{13}C NMR spectra of compounds **8** and **11**
11. References

1. Supplementary Table

Table S1. The ADMET evaluation of compounds [a].

Compound	MW	miLog *P*	TPSA	HBD	HBA	Violations	Rotatable bonds	Log *BB* [b]
**8a**	272.30	0.22	71.33	2	5	0	4	-0.88
**8b**	302.33	0.77	80.56	2	6	0	5	-0.94
**8c**	302.33	0.25	80.56	2	6	0	5	-1.02
**8d**	302.33	0.27	80.56	2	6	0	5	-1.01
**8e**	378.43	2.37	80.56	2	6	0	7	-0.69
**8f**	378.43	1.84	80.56	2	6	0	7	-0.77
**8g**	378.43	1.87	80.56	2	6	0	7	-0.77
**8h**	316.36	0.65	80.56	2	6	0	6	-0.95
**8i**	330.38	1.15	80.56	2	6	0	7	-0.88
**8j**	326.35	0.43	80.56	2	6	0	6	-0.99
**8k**	384.48	2.56	80.56	2	6	0	7	-0.66
**8l**	396.42	2.01	80.56	2	6	0	7	-0.75
**8m**	396.42	2.03	80.56	2	6	0	7	-0.74
**8n**	412.87	2.52	80.56	2	6	0	7	-0.67
**8o**	412.87	2.55	80.56	2	6	0	7	-0.67
**8p**	414.41	2.12	80.56	2	6	0	7	-0.73
**8q**	414.41	2.12	80.56	2	6	0	7	-0.73
**8r**	446.43	2.76	80.56	2	6	0	8	-0.63
**8s**	392.45	2.29	80.56	2	6	0	7	-0.71
**8t**	392.45	2.32	80.56	2	6	0	7	-0.70
**8u**	420.51	3.38	80.56	2	6	0	8	-0.54
**8v**	434.54	3.58	80.56	2	6	0	8	-0.51
**8w**	288.30	0.71	91.56	3	6	0	4	-1.11
**8x**	288.30	-0.29	91.56	3	6	0	4	-1.26
**8y**	288.30	-0.26	91.56	3	6	0	4	-1.26
**11a**	273.29	-0.95	84.22	2	6	0	4	-1.25
**11b**	273.29	-1.02	84.22	2	6	0	4	-1.26
**11c**	273.29	-1.07	84.22	2	6	0	4	-1.27
**DFP**	139.15	-0.60	42.23	1	3	0	0	-0.58

[a] Predicted by Molinspiration property engine V2018.10.

[b] Log *BB* = -0.0148 TPSA + 0.152 cLog *P* + 0.139

Table S2. The standard concentration-absorbance curve for each compound.

Compound	λ_{max} (nm)	Standard curve	R^2
donepezil	270	y = 0.0236x + 0.0181	0.9990
testosterone	249	y = 0.0374x + 0.0289	0.9994
tacrine	324	y = 0.0333x + 0.0118	0.9998
hydrocortisone	247	y = 0.0373x + 0.0353	0.9999
piroxicam	288	y = 0.0369x + 0.0006	0.9990
atenolol	275	y = 0.0036x + 0.0198	0.9980
theophylline	270	y = 0.0446x + 0.0208	0.9990
**8g**	244	y = 0.0195x + 0.0294	0.9982

2. Chemistry

2.1 Materials and instruments

All the reagents and solvents were purchased from Sinopharm Chemical Co., Ltd., Energy Chemical Co., Ltd. and Aladdin Chemical Co., Ltd. They were all used without further purification. ^{1}H and ^{13}C NMR were obtained by using Varian and Bruker instrument at 400, 600 and 100, 150 MHz, respectively, where tetramethylsilane (TMS) was used as an internal standard. Melting points were measured on a Büchi B-540 capillary melting point apparatus. High-resolution mass spectra (HRMS) were measured on Shimadzu LCMSIT-TOF mass spectrometer or Bruker micro OTOF-Q II instrument. The pK_a and log β were performed by an automatic titration system based on spectrophotometry (an autoburette, a Mettler Toledo pH meter and a luminescence 759s UV-Vis spectrophotometer), which controlled by a Visual Basic program. Enzyme activity and ROS were measured by multifunctional microplate (TECAN SPARK), flow cytometry (Becton-Dickinson FACS Calibur) and inverted biological microscope (Becton-Dickinson IX51), respectively. The purity of benzamide-HPO hybrids was determined by analytical HPLC (Agilent system 1200) coupled with UV-vis/DAD using C18 reverse-phase column [IRELAND (5 μm, 4.6 mm × 150 mm)]. The total run was monitored at wavelengths 254 nm with the mobile phase consisted of a 40% acetonitrile/60%water (0.2% trifluoroacetic acid) and a flow rate of 1 mL/min. The purities of the benzamide-HPO hybrids were over 98%.

2.2 General synthetic procedure for 3

The mixture of maltol **1** (7.56 g, 60 mmol) and anhydrous K_2CO_3 (16.56 g, 120 mmol) in DMF (50 mL) with 4-methoxylbenzyl chloride (14.04g, 90 mmol) dropwise was stirred for 2 h at 80 °C. When completed, the reaction mixture was quenched by water (100 mL) and extracted by EtOAc (4×100 mL). The combined organic layers were washed with water (3×100 mL), saturated brine (3×100 mL), dried over Na_2SO_4 and concentrated to dryness to obtain **2** as a yellow oil.

A mixture of **2** (20 mmol), ethylene diamine (1.26 g, 21 mmol) and NaOH (0.72 g, 18 mmol) in ethanol (20 mL) and water (18 mL) was reacted at 70 °C for 1.5 h. The mixture was concentrated to dryness and purified by silica gel chromatography (DCM : MeOH : 25% NH_3 = 10:1:0.1) to afford **3** as a yellow oil.

2.2.1 3-((4-Methoxybenzyl)oxy)-2-methyl-4*H*-pyran-4-one (**2**)

Yellow oil, yield 97%; ^{1}H NMR (400 MHz, $CDCl_3$) δ 7.58 (d, J = 5.6 Hz, 1H), 7.30 (d, J = 8.4 Hz, 2H), 6.85 (d, J = 8.8 Hz, 2H), 6.36 (d, J = 5.6 Hz, 1H), 5.10 (s, 2H), 3.80 (s, 3H), 2.06 (s, 3H); ^{13}C NMR (100 MHz, $CDCl_3$) δ 175.3, 160.0, 159.9, 153.5, 143.8, 130.9, 129.2, 117.3, 113.9, 73.25, 55.4, 15.0.

2.2.2 1-(2-Aminoethyl)-3-((4-methoxybenzyl)oxy)-2-methylpyridin-4(1*H*)-one (**3**)

Yellow oil, yield 52%; ^{1}H NMR (400 MHz, DMSO-d_6) δ 7.53 (d, J = 7.6 Hz, 1H), 7.31 (d, J = 8.4 Hz, 2H), 6.90 (d, J = 8.4 Hz, 2H), 6.11 (d, J = 7.2 Hz, 1H), 4.95 (s, 2H), 3.80 (t, J = 6.8 Hz, 2H), 3.75 (s, 3H), 2.73 (t, J = 6.8 Hz, 2H), 2.14 (s, 3H); ^{13}C NMR (100 MHz, DMSO-d_6) δ 171.8, 158.9, 145.0, 140.6, 139.6, 130.1, 129.8, 115.5, 113.5, 71.3, 55.3, 55.0, 41.9, 12.0.

2.3 General synthetic procedures for 5a-r

A mixture of KOH (1.12 g, 20 mmol), alkyl bromides or benzyl bromides derivatives (20 mmol) and *o*-, *m*- or *p*-hydroxybenzoic acids **4a-c** (1.38g, 10 mmol) was refluxed in ethanol (20 mL) and water (10 mL) for 5-30 h until the disappearance of **4a-c**. Then water (30 mL) was added

and the reaction mixture was acidified to pH 2 using concentrated HCl. The precipitate formed was filtrated, washed with water and *n*-hexane, and dried to yield acids **5a-r** as white solids.

2.3.1 2-(Benzyloxy)benzoic acid (**5a**)

White solid, yield 45%, m.p. 77-79 °C; ^{1}H NMR (400 MHz, $CDCl_3$) δ 10.8 (s, 1H), 8.23-8.20 (d, J = 7.8 Hz, 1H), 7.57-7.56 (m, 1H), 7.45-7.42 (m, 5H), 7.18-7.12 (m, 2H), 5.26 (s, 2H); ^{13}C NMR (100 MHz, $CDCl_3$) δ 165.3, 157.3, 135.0, 134.2, 133.8, 129.16, 129.12, 127.8, 122.4, 117.9, 113.0, 72.1. [1]

2.3.2 3-(Benzyloxy)benzoic acid (**5b**)

White solid, yield 58%, m.p. 135-137 °C; ^{1}H NMR (400 MHz, DMSO-d_6) δ 12.98 (s, 1H), 7.54 (d, J = 8.0 Hz, 2H), 7.46 (d, J = 7.6 Hz, 2H), 7.40 (q, J = 7.8 Hz, 3H), 7.33 (t, J = 7.0 Hz, 1H), 7.28-7.25 (m, 1H), 5.16 (s, 2H); ^{13}C NMR (100 MHz, DMSO-d_6) δ 167.1, 158.3, 136.8, 132.2, 129.7, 128.5, 127.9, 127.7, 121.8, 119.7, 114.9, 69.4. [1]

2.3.3 4-(Benzyloxy)benzoic acid (**5c**)

White solid, yield 91%, m.p. 185-187 °C; ^{1}H NMR (400 MHz, DMSO-d_6) δ 7.89 (d, J = 8.8 Hz, 2H), 7.46 (d, J = 7.2 Hz, 2H), 7.40 (t, J = 7.2 Hz, 2H), 7.36-7.32 (m, 1H), 7.10 (d, J = 8.8 Hz, 2H), 5.18 (s, 2H); ^{13}C NMR (100 MHz, DMSO-d_6) δ 167.0, 162.0, 136.6, 131.4, 128.5, 128.0, 127.8, 123.2, 114.6, 69.5. [2]

2.3.4 4-Ethoxybenzoic acid (**5d**)

White solid, yield 76%, m.p. 198-200 °C; ^{1}H NMR (400 MHz, DMSO-d_6) δ 12.60 (s, 1H), 7.90 (d, J = 8.8 Hz, 2H), 7.01 (d, J = 8.8 Hz, 2H), 4.11 (q, J = 6.8 Hz,2H), 1.36 (t, J = 6.8 Hz, 3H); ^{13}C NMR (100 MHz, DMSO-d_6) δ 167.0, 162.2, 131.4, 122.8, 114.2, 63.4, 14.5. [3]

2.3.5 4-Propoxybenzoic acid (**5e**)

White solid, yield 70%, m.p. 146-148 °C; ^{1}H NMR (400 MHz, DMSO-d_6) δ 12.60 (br. s, 1 H), 7.88 (d, J = 8.9 Hz), 7.00 (d, J = 8.9 Hz), 3.99 (t, J = 6.9 Hz), 1.78-1.70 (m, 2 H), 0.98 (t, J = 6.9 Hz). [3]

2.3.6 4-(Prop-2-yn-1-yloxy)benzoic acid (**5f**)

White solid, yield 69%, m.p. 213-215 °C; ^{1}H NMR (400 MHz, DMSO-d_6) δ 12.63 (s, 1H), 7.91 (d, J = 8.8 Hz, 2H), 7.06 (d, J = 8.8 Hz, 2H), 4.89 (s, 2H), 3.60 (s, 1H); ^{13}C NMR (100 MHz, DMSO-d_6) δ 166.9, 160.7, 131.3, 123.7, 114.7, 78.7, 78.6, 55.7. [4]

2.3.7 4-(Cyclohexylmethoxy)benzoic acid (**5g**)

White solid, yield 38%, m.p. 214-216 °C; ^{1}H NMR (400 MHz, DMSO-d_6) δ 12.57 (s, 1H), 7.87 (d, J = 8.8 Hz, 2H), 6.98 (d, J = 8.8 Hz, 2H), 3.82 (d, J = 6.0 Hz, 2H), 1.78 (d, J = 12.4 Hz, 2H), 1.71 (d, J = 12.4 Hz, 3H), 1.62 (d, J = 11.6 Hz, 1H), 1.29-1.10 (m, 3H), 1.02 (q, J = 11.6 Hz, 2H); ^{13}C NMR (100 MHz, DMSO-d_6) δ 167.0, 162.4, 131.3, 122.8, 114.2, 72.9, 38.9, 37.0, 29.2, 26.0, 25.2. [5]

2.3.8 4-((3-Fluorobenzyl)oxy)benzoic acid (**5h**)

White solid, yield 97%, m.p. 194-196 °C; ^{1}H NMR (400 MHz, DMSO-d_6) δ 7.91-7.88 (m, 2H), 7.44 (q, J = 7.6 Hz, 1H), 7.31-7.28 (m, 2H), 7.20-7.14 (m, 1H), 7.11-7.08 (m, 2H), 5.20 (s, 2H); ^{13}C NMR (100 MHz, DMSO-d_6) δ 167.0, 163.4 (d, ^1J = 242.1 Hz), 161.6, 139.5 (d, ^3J = 7.4 Hz), 131.3, 130.5 (d, ^3J = 8.1 Hz), 123.6 (d, ^4J = 2.8 Hz), 114.8 (d, ^2J = 20.8 Hz), 114.6, 114.4 (d, ^2J = 21.6 Hz), 68.6 (d, ^4J = 2.0 Hz). [2]

2.3.9 4-((4-Fluorobenzyl)oxy)benzoic acid (**5i**)

White solid, yield 95%, m.p. 211-213 °C; ^{1}H NMR (400 MHz, DMSO-d_6) δ 12.64 (s, 1H), 7.91-7.88 (m, 2H), 7.51 (dd, J = 8.4, 6.0 Hz, 2H), 7.22 (t, J = 8.8 Hz, 2H), 7.10-7.07 (m, 2H), 5.16

(s, 2H); ^{13}C NMR (100 MHz, DMSO-d_6) δ 167.0, 163.1 (d, ^1J = 242.4 Hz), 161.8, 132.8 (d, ^4J = 3.1 Hz), 131.3, 130.1 (d, ^3J = 8.2 Hz), 123.2, 115.4 (d, ^2J = 21.3 Hz), 114.6, 68.7. [2]

2.3.10 4-((3-Chlorobenzyl)oxy)benzoic acid (**5j**)

White solid, yield 98%, m.p. 198-200 °C; ^{1}H NMR (400 MHz, DMSO-d_6) δ 12.65 (s, 1H), 7.92-7.89 (m, 2H), 7.53 (s, 1H), 7.45-7.38 (m, 3H), 7.11-7.08 (m, 2H), 5.20 (s, 2H); ^{13}C NMR (100 MHz, DMSO-d_6) δ 166.9, 161.7, 139.1, 133.2, 131.4, 130.4, 127.9, 127.4, 126.3, 123.4, 114.6, 68.5. [6]

2.3.11 4-((4-Chlorobenzyl)oxy)benzoic acid (**5k**)

White solid, yield 97%, m.p. 219-221 °C; ^{1}H NMR (400 MHz, DMSO-d_6) δ 12.69 (s, 1H), 7.91-7.88 (m, 2H), 7.50-7.44 (m, 4H), 7.10-7.06 (m, 2H), 5.18 (s, 2H); ^{13}C NMR (100 MHz, DMSO-d_6) δ 167.0, 161.7, 135.6, 132.6, 131.3, 129.6, 128.5, 123.4, 114.6, 68.6. [2]

2.3.12 4-((2,5-Difluorobenzyl)oxy)benzoic acid (**5l**)

White solid, yield 99%, m.p. 194-196 °C; ^{1}H NMR (400 MHz, DMSO-d_6) δ 12.67 (s, 1H), 7.93-7.89 (m, 2H), 7.45-7.40 (m, 1H), 7.35-7.23 (m, 2H), 7.14-7.11 (m, 2H), 5.20 (s, 2H); ^{13}C NMR (100 MHz, DMSO-d_6) δ 166.9, 161.5, 159.3 (dd, ^1J = 238.8 Hz, ^4J = 2.1 Hz), 157.6 (dd, ^1J = 241.0 Hz, ^4J = 2.4 Hz), 156.9 (d, ^4J = 2.2 Hz), 155.2 (d, ^4J = 2.3 Hz), 131.4, 125.5 (d, ^3J = 8.0 Hz), 125.3 (d, ^3J = 8.2 Hz), 123.6, 117.2 (d, ^3J = 8.8 Hz), 117.0, 116.9, 116.9 (d, ^2J = 23.1 Hz), 116.9 (d, ^2J = 24.7 Hz), 116.8 (d, ^2J = 24.0 Hz), 114.6, 63.4 (d, ^4J = 3.0 Hz). [7]

2.3.13 4-((3,5-Difluorobenzyl)oxy)benzoic acid (**5m**)

White solid, yield 83%, m.p. 220-222 °C; ^{1}H NMR (400 MHz, DMSO-d_6) δ 12.64 (s, 1H), 7.92-7.89 (m, 2H), 7.21-7.17 (m, 3H), 7.11-7.08 (m, 2H), 5.21 (s, 2H); ^{13}C NMR (100 MHz, DMSO-d_6) δ 166.9, 163.7 (dd, ^1J = 245.0 Hz, ^3J = 13.2 Hz), 161.5, 141.2 (t, ^3J = 9.2 Hz), 131.4, 123.6, 114.6, 110.6 (dd, ^2J = 25.5 Hz, ^3J = 7.0 Hz), 103.3 (t, ^2J = 25.5 Hz), 68.0. [7]

2.3.14 4-((4-(Trifluoromethyl)benzyl)oxy)benzoic acid (**5n**)

White solid, yield 90%, m.p. 220-222 °C; ^{1}H NMR (400 MHz, DMSO-d_6) δ 12.66 (s, 1H), 7.92-7.89 (m, 2H), 7.76 (d, J = 8.4 Hz, 2H), 7.68 (d, J = 8.4 Hz, 2H), 7.12-7.09 (m, 2H), 5.30 (s, 2H); ^{13}C NMR (100 MHz, DMSO-d_6) δ 166.9, 161.6, 141.4, 131.4, 128.5 (q, ^2J = 31.5 Hz), 128.1, 125.6 (d, ^1J = 270.4 Hz), 125.4 (q, ^3J = 3.6 Hz), 123.5, 114.6, 68.5. [2]

2.3.15 4-((3-Methylbenzyl)oxy)benzoic acid (**5o**)

White solid, yield 91%, m.p. 168-170 °C; ^{1}H NMR (400 MHz, DMSO-d_6) δ 12.64 (s, 1H), 7.89 (d, J = 8.8 Hz, 2H), 7.30-7.22 (m, 3H), 7.15 (d, J = 7.2 Hz, 1H), 7.08(d, J = 8.0 Hz, 2H), 5.12 (s, 2H), 2.31 (s, 3H); ^{13}C NMR (100 MHz, DMSO-d_6) δ 167.0, 162.0, 137.7, 136.5, 131.4, 128.7, 128.4, 124.9, 123.2, 114.6, 69.5, 21.0. [2]

2.3.16 4-((4-Methylbenzyl)oxy)benzoic acid (**5p**)

White solid, yield 85%, m.p. 217-219 °C; ^{1}H NMR (400 MHz, DMSO-d_6) δ 7.89 (d, J = 8.4 Hz, 2H), 7.34 (d, J = 7.8 Hz, 2H), 7.20 (d, J = 8.0 Hz, 2H), 7.07 (d, J = 8.4 Hz, 2H), 5.12 (s, 2H), 2.30 (s, 3H); ^{13}C NMR (100 MHz, DMSO-d_6) δ 167.0, 161.9, 137.3, 133.5, 131.3, 129.0, 127.9, 123.2, 114.6, 69.5, 20.8. [6]

2.3.17 4-((4-Isopropylbenzyl)oxy)benzoic acid (**5q**)

White solid, yield 71%, m.p. 206-208 °C; ^{1}H NMR (400 MHz, DMSO-d_6) δ 12.61 (s, 1H), 7.91-7.88 (m, 2H), 7.37 (d, J = 8.0 Hz, 2H), 7.26 (d, J = 8.0 Hz, 2H), 7.10-7.07 (m, 2H), 5.12 (s, 2H), 2.88 (hept, J = 7.8 Hz, 1H), 1.20 (s, 3H), 1.18 (s, 3H); ^{13}C NMR (100 MHz, DMSO-d_6) δ 167.0, 162.0, 148.3, 133.9, 131.3, 128.0, 126.4, 123.1, 114.6, 69.4, 33.2, 23.8. [7]

2.3.18 4-((4-(*tert*-Butyl)benzyl)oxy)benzoic acid (**5r**)

White solid, yield 53%, m.p. 233-235 °C; ^{1}H NMR (400 MHz, DMSO-d_6) δ 12.63 (s, 1H), 7.89 (d, J = 7.2 Hz, 2H), 7.40 (d, J = 6.4 Hz, 2H), 7.38 (d, J = 4.8 Hz, 2H), 7.09 (d, J = 5.2 Hz, 2H), 5.13 (s, 2H), 1.27 (m, 9H); ^{13}C NMR (100 MHz, DMSO-d_6) δ 167.0, 162.0, 150.5, 133.5, 131.4, 127.7, 125.2, 123.1, 114.6, 69.3, 34.3, 31.1.[8]

2.4 General synthetic procedures for 7a-v and 10a-c

DCC (0.226 g, 1.1 mmol), 2-mercaptothiazoline (0.130 g, 1.1 mmol), and a catalytic amount of DMAP (5 mg) were successively added to the DCM (10 mL) solution of benzoic acids **5a-r/6a-d** or pyridinecarboxylic acids **9a-c** (1 mmol). After stirring at room temperature for 24 h, *N,N'*-dicyclohexylurea (DCU) was filtered and the filtrate was added to the DCM (10 mL) solution of amine **3** (0.288g, 1 mmol), and continued to be stirred for 24 h. The solvent was removed under vacuum and the residue was purified by silica gel chromatography (DCM : MeOH = 100:1-20:1 gradient elution) to afford **7a-v** or **10a-c** as yellow solids.

2.4.1 *N*-(2-(3-((4-methoxybenzyl)oxy)-2-methyl-4-oxopyridin-1(4*H*)-yl)ethyl) benzamide (**7a**)

Yellow solid, yield 67%, m.p. 149-151 °C; ^{1}H NMR (400 MHz, CDCl$_3$) δ 9.23-9.07 (m, 1H), 8.06 (d, J = 7.6 Hz, 2H), 7.52-7.45 (m, 1H), 7.44-7.40 (m, 2H), 7.27 (s, 1H), 7.25 (s, 1H), 7.05 (d, J = 7.2 Hz, 1H), 6.82 (d, J = 8.4 Hz, 2H), 6.04 (t, J = 8.8 Hz, 1H), 4.89 (s, 2H), 4.04 (t, J = 4.8 Hz, 2H), 3.79 (s, 3H), 3.64 (q, J = 5.2 Hz, 2H), 2.12 (s, 3H); ^{13}C NMR (100 MHz, CDCl$_3$) δ 172.9, 168.7, 159.7, 146.1, 142.0, 139.4, 134.0, 131.7, 130.8, 129.5, 128.6, 127.9, 116.5, 113.8, 73.0, 55.4, 52.8, 40.3, 12.7; ESI-HRMS: m/z calcd for $C_{23}H_{25}N_2O_4$ [M+H]$^+$: 393.1809; found: 393.1808.

2.4.2 2-Methoxy-*N*-(2-(3-((4-methoxybenzyl)oxy)-2-methyl-4-oxopyridin-1(4*H*)-yl) ethyl) benzamide (**7b**)

White solid, yield 58%, m.p. 112-114 °C; ^{1}H NMR (400 MHz, CDCl$_3$) δ 8.14 (dd, J = 7.6, 1.2 Hz, 1H), 7.96 (t, J = 6.0 Hz, 1H), 7.49-7.44 (m, 1H), 7.30 (d, J = 8.4 Hz, 2H), 7.19 (d, J = 7.2 Hz, 1H), 7.08 (t, J = 7.6 Hz, 1H), 6.96 (d, J = 8.4 Hz, 1H), 6.82 (d, J = 8.8 Hz, 2H), 6.35 (d, J = 7.6 Hz, 1H), 5.14 (s, 2H), 4.03 (t, J = 6.0 Hz, 2H), 3.87 (s, 3H), 3.77 (s, 3H), 3.62 (q, J = 6.0 Hz, 2H), 2.13 (s, 3H); ^{13}C NMR (100 MHz, CDCl$_3$) δ 173.6, 166.3, 159.6, 157.7, 146.3, 141.0, 138.8, 133.6, 132.2, 130.9, 129.9, 121.6, 120.6, 117.2, 113.7, 111.6, 72.7, 56.2, 55.4, 52.4, 40.3, 12.7; ESI-HRMS: m/z calcd for $C_{24}H_{27}N_2O_5$ [M+H]$^+$: 423.1914; found: 423.1895.

2.4.3 3-Methoxy-*N*-(2-(3-((4-methoxybenzyl)oxy)-2-methyl-4-oxopyridin-1(4*H*)-yl) ethyl) benzamide (**7c**)

White solid, yield 88%, m.p. 116-118 °C; ^{1}H NMR (400 MHz, CDCl$_3$) δ 9.29 (t, J = 5.6 Hz, 1H), 7.65 (d, J = 6.8 Hz, 2H), 7.32 (t, J = 8.0 Hz, 1H), 7.24 (s, 2H), 7.05-7.01 (m, 2H), 6.81 (d, J = 8.4 Hz, 2H), 6.04 (d, J = 7.6 Hz, 1H), 4.88 (s, 2H), 4.02 (t, J = 5.0 Hz, 2H), 3.83 (s, 3H), 3.79 (s, 3H), 3.63 (q, J = 5.2 Hz, 2H), 2.11 (s, 3H); ^{13}C NMR (100 MHz, CDCl$_3$) δ 172.9, 168.5, 159.9, 159.8, 146.1, 142.0, 139.4, 135.4, 130.7, 129.6, 129.5, 120.1, 118.1, 116.5, 113.8, 112.8, 73.0, 55.6, 55.4, 52.8, 40.3, 12.6; ESI-HRMS: m/z calcd for $C_{24}H_{27}N_2O_5$ [M+H]$^+$: 423.1914; found: 423.1899.

2.4.4 4-Methoxy-*N*-(2-(3-((4-methoxybenzyl)oxy)-2-methyl-4-oxopyridin-1(4*H*)-yl) ethyl) benzamide (**7d**)

White solid, yield 83%, m.p. 115-117 °C; ^{1}H NMR (400 MHz, CDCl$_3$) δ 9.05 (t, J = 5.6 Hz, 1H), 8.06 (d, J = 8.4 Hz, 2H), 7.25 (d, J = 8.4 Hz, 2H), 7.03 (d, J = 7.6 Hz, 1H), 6.91 (d, J = 8.4 Hz, 2H), 6.82 (d, J = 8.8 Hz, 2H), 6.05 (d, J = 7.2 Hz, 1H), 4.89 (s, 2H), 4.02 (t, J = 4.8 Hz, 2H), 3.82 (s, 3H), 3.78 (s, 3H), 3.61 (q, J = 5.4 Hz, 2H), 2.11 (s, 3H); ^{13}C NMR (100 MHz, CDCl$_3$) δ

173.0, 168.3, 162.4, 159.7, 146.1, 141.9, 139.4, 130.7, 129.7, 129.6, 126.4, 116.6, 113.8, 113.8, 73.0, 55.5, 55.4, 52.9, 40.3, 12.6; ESI-HRMS: m/z calcd for $C_{24}H_{27}N_2O_5$ $[M+H]^+$: 423.1914; found: 423.1891.

2.4.5 2-(Benzyloxy)-*N*-(2-(3-((4-methoxybenzyl)oxy)-2-methyl-4-oxopyridin-1(4*H*) -yl)ethyl) benzamide (**7e**)

White solid, yield 30%, m.p. 95-97 °C; ^{1}H NMR (400 MHz, $CDCl_3$) δ 8.19 (dd, *J* = 8.0, 1.2 Hz, 1H), 8.11 (t, *J* = 6.0 Hz, 1H), 7.47 (t, *J* = 8.4 Hz, 1H), 7.42-7.34 (m, 3H), 7.34-7.29 (m, 4H), 7.11 (t, *J* = 7.6 Hz, 1H), 7.04 (dd, *J* = 8.0, 4.4 Hz, 2H), 6.82 (d, *J* = 8.4 Hz, 2H), 6.33 (d, *J* = 7.6 Hz, 1H), 5.13 (d, *J* = 2.4 Hz, 4H), 3.90 (t, *J* = 6.4 Hz, 2H), 3.78 (s, 3H), 3.45 (q, *J* = 6.0 Hz, 2H), 2.05 (s, 3H); ^{13}C NMR (100 MHz, $CDCl_3$) δ 173.5, 166.2, 159.6, 157.1, 146.3, 140.8, 138.5, 135.5, 133.6, 132.4, 130.8, 130.0, 129.2, 129.1, 127.9, 121.9, 120.8, 117.4, 113.8, 113.0, 72.7, 71.7, 55.4, 52.1, 40.3, 12.6; ESI-HRMS: m/z calcd for $C_{30}H_{31}N_2O_5$ $[M+H]^+$: 499.2227; found: 499.2276.

2.4.6 3-(Benzyloxy)-*N*-(2-(3-((4-methoxybenzyl)oxy)-2-methyl-4-oxopyridin-1(4*H*) -yl)ethyl) benzamide (**7f**)

White solid, yield 80%, m.p. 73-75 °C; ^{1}H NMR (400 MHz, $CDCl_3$) δ 9.34 (t, *J* = 5.6 Hz, 1H), 7.80 (s, 1H), 7.69 (d, *J* = 7.6 Hz, 1H), 7.38 (d, *J* = 7.2 Hz, 2H), 7.35-7.27 (m, 4H), 7.24 (d, *J* = 8.8 Hz, 2H), 7.10 (dd, *J* = 8.0, 2.8 Hz, 1H), 6.97 (d, *J* = 7.6 Hz, 1H), 6.81 (d, *J* = 8.4 Hz, 2H), 6.05 (d, *J* = 7.2 Hz, 1H), 5.10 (s, 2H), 4.87 (s, 2H), 4.00 (t, *J* = 4.8 Hz, 2H), 3.78 (s, 3H), 3.62 (q, *J* = 5.2 Hz, 2H), 2.10 (s, 3H); ^{13}C NMR (100 MHz, $CDCl_3$) δ 172.9, 168.4, 159.7, 159.0, 146.1, 142.0, 139.4, 136.9, 135.4, 130.7, 129.7, 129.5, 128.7, 128.1, 127.7, 120.4, 118.9, 116.5, 113.9, 113.8, 73.0, 70.2, 55.4, 52.8, 40.3, 12.6; ESI-HRMS: m/z calcd for $C_{30}H_{31}N_2O_5$ $[M+H]^+$: 499.2227; found: 499.2253.

2.4.7 4-(Benzyloxy)-*N*-(2-(3-((4-methoxybenzyl)oxy)-2-methyl-4-oxopyridin-1(4*H*) -yl)ethyl) benzamide (**7g**)

White solid, yield 71%, m.p. 124-126 °C; ^{1}H NMR (400 MHz, $CDCl_3$) δ 9.00-8.90 (m, 1H), 8.06 (d, *J* = 3.6 Hz, 2H), 8.04 (t, *J* = 3.6 Hz, 2H), 7.41 (d, *J* = 7.6 Hz, 3H), 7.37 (d, *J* = 7.6 Hz, 1H), 7.33 (d, *J* = 6.8 Hz, 1H), 7.27 (d, *J* = 6.0 Hz, 3H), 7.03 (d, *J* = 7.6 Hz, 1H), 6.99 (d, *J* = 8.4 Hz, 2H), 6.83 (d, *J* = 8.4 Hz, 2H), 6.06 (dd, *J* = 7.6, 3.2 Hz, 1H), 5.07 (s, 2H), 4.90 (d, *J* = 2.4 Hz, 2H), 4.03 (t, *J* = 4.8 Hz, 2H), 3.78 (s, 3H), 3.62 (q, *J* = 5.2 Hz, 2H), 2.12 (s, 3H); ^{13}C NMR (100 MHz, $CDCl_3$) δ 173.0, 168.3, 161.6, 159.7, 146.1, 141.9, 139.4, 136.6, 130.7, 129.8, 129.5, 128.8, 128.2, 127.6, 126.6, 116.6, 114.6, 113.8, 73.0, 70.1, 55.4, 52.9, 40.3, 12.6; ESI-HRMS: m/z calcd for $C_{30}H_{31}N_2O_5$ $[M+H]^+$: 499.2227; found: 499.2228.

2.4.8 4-Ethoxy-*N*-(2-(3-((4-methoxybenzyl)oxy)-2-methyl-4-oxopyridin-1(4*H*)-yl) ethyl) benzamide (**7h**)

White solid, yield 64%, m.p. 97-98 °C; ^{1}H NMR (400 MHz, $CDCl_3$) δ 9.01 (d, *J* = 6.0 Hz, 1H), 8.03 (d, *J* = 8.8 Hz, 2H), 7.25 (d, *J* = 8.4 Hz, 2H), 7.03 (d, *J* = 7.2 Hz, 1H), 6.89 (d, *J* = 8.4 Hz, 2H), 6.82 (d, *J* = 8.4 Hz, 2H), 6.05 (d, *J* = 7.6 Hz, 1H), 4.90 (s, 2H), 4.07-4.01 (m, 4H), 3.78 (s, 3H), 3.61 (q, *J* = 5.2 Hz, 2H), 2.11 (s, 3H), 1.41 (t, *J* = 6.8 Hz, 3H); ^{13}C NMR (100 MHz, $CDCl_3$) δ 173.0, 168.3, 161.9, 159.7, 146.1, 141.9, 139.4, 130.7, 129.7, 129.6, 126.1, 116.6, 114.2, 113.8, 73.0, 63.7, 55.4, 52.8, 40.3, 14.8, 12.6; ESI-HRMS: m/z calcd for $C_{25}H_{29}N_2O_5$ $[M+H]^+$: 437.2071; found: 437.2085.

2.4.9 *N*-(2-(3-((4-methoxybenzyl)oxy)-2-methyl-4-oxopyridin-1(4*H*)-yl)ethyl)-4-propoxybenzamide (**7i**)

White solid, yield 65%, m.p. 92-94 °C; $_1$H NMR (400 MHz, $CDCl_3$) δ 8.92 (t, *J* = 5.6 Hz,

1H), 8.02 (d, *J* = 8.4 Hz, 2H), 7.27 (d, *J* = 8.4 Hz, 2H), 7.04 (d, *J* = 7.6 Hz, 1H), 6.90 (d, *J* = 8.4 Hz, 2H), 6.82 (d, *J* = 8.4 Hz, 2H), 6.06 (d, *J* = 7.2 Hz, 1H), 4.91 (s, 2H), 4.03 (t, *J* = 4.8 Hz, 2H), 3.93 (t, *J* = 6.4 Hz, 2H), 3.79 (s, 3H), 3.62 (q, *J* = 5.2 Hz, 2H), 2.11 (s, 3H), 1.80 (q, *J* = 7.2 Hz, 2H), 1.03 (t, *J* = 7.6 Hz, 3H); ^{13}C NMR (100 MHz, $CDCl_3$) δ 173.0, 168.3, 162.1, 159.7, 146.1, 141.9, 139.4, 130.7, 129.7, 129.6, 126.1, 116.6, 114.3, 113.8, 73.0, 69.7, 55.4, 52.8, 40.3, 22.6, 12.7, 10.6; ESI-HRMS: m/z calcd for $C_{26}H_{31}N_2O_5$ $[M+H]^+$: 451.2227; found: 451.2249.

2.4.10 *N*-(2-(3-((4-methoxybenzyl)oxy)-2-methyl-4-oxopyridin-1(4*H*)-yl)ethyl)-4-(prop-2-yn-1-yloxy)benzamide (**7j**)

White solid, yield 75%, m.p. 112-114 °C; ^{1}H NMR (400 MHz, $CDCl_3$) δ 9.13 (t, *J* = 5.6 Hz, 1H), 8.07 (d, *J* = 8.4 Hz, 2H), 7.25 (d, *J* = 8.8 Hz, 2H), 7.04 (d, *J* = 7.6 Hz, 1H), 6.98 (d, *J* = 8.8 Hz, 2H), 6.82 (d, *J* = 8.4 Hz, 2H), 6.06 (d, *J* = 7.6 Hz, 1H), 4.89 (s, 2H), 4.70 (d, *J* = 2.0 Hz, 2H), 4.03 (d, *J* = 4.4 Hz, 2H), 3.79 (s, 3H), 3.62 (q, *J* = 5.2 Hz, 2H), 2.12 (s, 3H), 1.37 (s, 1H); ^{13}C NMR (100 MHz, $CDCl_3$) δ 172.9, 168.1, 160.3, 159.8, 146.1, 142.0, 139.5, 130.7, 129.7, 129.5, 127.3, 116.6, 114.7, 113.9, 78.2, 76.1, 73.1, 55.9, 55.4, 40.3, 29.8, 12.7; ESI-HRMS: m/z calcd for $C_{26}H_{27}N_2O_5$ $[M+H]^+$: 447.1914; found: 447.1951.

2.4.11 4-(Cyclohexylmethoxy)-*N*-(2-(3-((4-methoxybenzyl)oxy)-2-methyl-4-oxopyridin-1(4*H*)-yl)ethyl)benzamide (**7k**)

White solid, yield 67%, m.p. 105-107 °C; ^{1}H NMR (400 MHz, $CDCl_3$) δ 9.00 (dt, *J* = 21.2, 5.6 Hz, 1H), 8.03 (dd, *J* = 8.8, 2.0 Hz, 2H), 7.25 (d, *J* = 8.4 Hz, 2H), 7.04 (dd, *J* = 7.6, 2.4 Hz, 1H), 6.89 (d, *J* = 8.4 Hz, 2H), 6.82 (d, *J* = 8.4 Hz, 2H), 6.06 (dd, *J* = 7.6, 2.8 Hz, 1H), 4.90 (d, *J* = 2.0 Hz, 2H), 4.02 (t, *J* = 4.8 Hz, 2H), 3.78 (s, 3H), 3.75 (d, *J* = 6.4 Hz, 2H), 3.61 (q, *J* = 5.2 Hz, 2H), 2.11 (s, 3H), 1.88-1.68 (m, 6H), 1.34-1.20 (m, 3H), 1.17-0.96 (m, 2H); ^{13}C NMR (100 MHz, $CDCl_3$) δ 172.9, 168.3, 162.2, 159.7, 146.1, 142.0, 139.4, 130.7, 129.7, 129.6, 126.0, 116.6, 114.3, 113.8, 73.7, 73.0, 55.4, 52.9, 40.3, 37.7, 30.0, 26.6, 25.9, 12.6; ESI-HRMS: m/z calcd for $C_{30}H_{37}N_2O_5$ $[M+H]^+$: 505.2697; found: 505.2718.

2.4.12 4-((3-Fluorobenzyl)oxy)-*N*-(2-(3-((4-methoxybenzyl)oxy)-2-methyl-4-oxopyridin-1(4*H*)-yl)ethyl)benzamide (**7l**)

White solid, yield 70%, m.p. 155-157 °C; ^{1}H NMR (400 MHz, $CDCl_3$) δ 9.12-9.00 (m, 1H), 8.08 (d, *J* = 4.4 Hz, 1H), 8.06 (t, *J* = 4.0 Hz, 1H), 7.33 (q, *J* = 7.6 Hz, 1H), 7.30-7.27 (m, 1H), 7.25 (s, 2H), 7.17 (d, *J* = 7.6 Hz, 1H), 7.13 (d, *J* = 9.6 Hz, 1H), 7.04-7.00 (m, 2H), 6.97 (d, *J* = 8.8 Hz, 2H), 6.82 (d, *J* = 8.0 Hz, 2H), 6.05 (dd, *J* = 8.0, 4.0 Hz, 1H), 5.06 (s, 2H), 4.89 (d, *J* = 3.6 Hz, 2H), 4.03 (t, *J* = 4.8 Hz, 2H), 3.78 (s, 3H), 3.62 (q, *J* = 5.2 Hz, 2H), 2.12 (s, 3H); ^{13}C NMR (100 MHz, $CDCl_3$) δ 173.0, 168.2, 164.3 (d, ^1J = 245.0 Hz), 161.3, 159.7, 146.1, 142.0, 139.4, 139.2 (d, ^3J = 7.5 Hz), 130.7, 130.4 (d, ^3J = 8.3 Hz), 129.8, 129.5, 126.9, 122.84 (d, ^4J = 2.8 Hz), 116.6, 115.2 (d, ^2J = 21.1 Hz), 114.6, 114.44 (d, ^2J = 22.0 Hz), 113.8, 73.0, 69.3, 55.4, 52.9, 40.3, 12.6; ESI-HRMS: m/z calcd for $C_{30}H_{30}FN_2O_5$ $[M+H]^+$: 517.2133; found: 517.2144.

2.4.13 4-((4-Fluorobenzyl)oxy)-*N*-(2-(3-((4-methoxybenzyl)oxy)-2-methyl-4-oxopyridin-1(4*H*)-yl)ethyl)benzamide (**7m**)

White solid, yield 73%, m.p. 126-128 °C; ^{1}H NMR (400 MHz, $CDCl_3$) δ 9.18 (s, 1H), 8.08 (d, *J* = 8.4 Hz, 2H), 7.38 (d, *J* = 6.0 Hz, 1H), 7.36 (d, *J* = 5.6 Hz, 1H), 7.25 (d, *J* = 6.0 Hz, 2H), 7.08-7.01 (m, 3H), 6.97 (d, *J* = 8.4 Hz, 2H), 6.82 (d, *J* = 8.4 Hz, 2H), 6.03 (d, *J* = 7.6 Hz, 1H), 5.02 (s, 2H), 4.88 (s, 2H), 4.02 (t, *J* = 4.8 Hz, 2H), 3.78 (s, 3H), 3.62 (q, *J* = 4.8 Hz, 2H), 2.12 (s, 3H); ^{13}C NMR (100 MHz, $CDCl_3$) δ 173.0, 168.2, 163.9 (d, ^1J = 245.3 Hz), 161.4, 159.7, 146.1, 142.0, 139.4, 132.4 (d, ^4J = 2.9 Hz), 130.7, 129.8, 129.5 (d, ^3J = 7.7 Hz), 126.8, 116.6, 115.8 (d,

^{2}J = 21.4 Hz), 114.6, 113.8, 106.7, 73.0, 69.5, 55.4, 52.8, 40.3, 12.6; ESI-HRMS: m/z calcd for $C_{30}H_{30}FN_2O_5$ [M+H]$^+$: 517.2133; found: 517.2150.

2.4.14 4-((3-Chlorobenzyl)oxy)-*N*-(2-(3-((4-methoxybenzyl)oxy)-2-methyl-4-oxopyridin-1(4*H*)-yl)ethyl)benzamide (**7n**)

White solid, yield 60%, m.p. 128-130 ºC; ^{1}H NMR (400 MHz, $CDCl_3$) δ 9.17 (s, 1H), 8.08 (d, *J* = 8.4 Hz, 2H), 7.41 (s, 1H), 7.30-7.26 (m, 4H), 7.25 (s, 1H), 7.02 (d, *J* = 7.6 Hz, 1H), 6.97 (d, *J* = 8.8 Hz, 2H), 6.82 (d, *J* = 8.4 Hz, 2H), 6.04 (d, *J* = 7.2 Hz, 1H), 5.03 (s, 2H), 4.88 (s, 2H), 4.03 (t, *J* = 4.0 Hz, 2H), 3.78 (s, 3H), 3.62 (q, *J* = 5.2 Hz, 2H), 2.12 (s, 3H).; ^{13}C NMR (100 MHz, $CDCl_3$) δ 173.0, 168.2, 161.2, 159.7, 146.1, 142.0, 139.4, 138.7, 134.7, 130.7, 130.1, 129.9, 129.5, 128.4, 127.5, 126.9, 125.5, 116.6, 114.6, 113.8, 73.0, 69.2, 55.4, 52.9, 40.3, 12.6; ESI-HRMS: m/z calcd for $C_{30}H_{30}ClN_2O_5$ [M+H]$^+$: 533.1838; found: 533.1836.

2.4.15 4-((4-Chlorobenzyl)oxy)-*N*-(2-(3-((4-methoxybenzyl)oxy)-2-methyl-4-oxopyridin-1(4*H*)-yl)ethyl)benzamide (**7o**)

White solid, yield 77%, m.p. 125-127 ºC; ^{1}H NMR (400 MHz, $CDCl_3$) δ 9.13 (s, 1H), 8.07 (d, *J* = 8.4 Hz, 2H), 7.34 (s, 4H), 7.27 (s, 1H), 7.25 (s, 1H), 7.02 (d, *J* = 7.6 Hz, 1H), 6.96 (d, *J* = 8.4 Hz, 2H), 6.82 (d, *J* = 8.0 Hz, 2H), 6.03 (d, *J* = 6.4 Hz, 1H), 5.03 (s, 2H), 4.88 (s, 2H), 4.03 (t, *J* = 4.4 Hz, 2H), 3.78 (s, 3H), 3.62 (q, *J* = 5.2 Hz, 2H), 2.11 (s, 3H).; ^{13}C NMR (100 MHz, $CDCl_3$) δ 173.0, 168.2, 161.3, 159.7, 146.1, 141.9, 139.4, 135.1, 134.1, 130.7, 129.8, 129.5, 128.9, 128.9, 126.9, 116.6, 114.6, 113.8, 73.0, 69.3, 55.4, 53.6, 40.3, 12.6; ESI-HRMS: m/z calcd for $C_{30}H_{30}ClN_2O_5$ [M+H]$^+$: 533.1838; found: 533.1838.

2.4.16 4-((2,5-Difluorobenzyl)oxy)-*N*-(2-(3-((4-methoxybenzyl)oxy)-2-methyl-4-oxopyridin-1(4*H*)-yl)ethyl)benzamide (**7p**)

White solid, yield 68%, m.p. 144-146 ºC; ^{1}H NMR (400 MHz, $CDCl_3$) δ 9.15 (t, *J* = 5.2 Hz, 1H), 8.10 (d, *J* = 8.8 Hz, 2H), 7.27 (s, 1H), 7.25 (d, *J* = 4.8 Hz, 2H), 7.23-7.18 (m, 1H), 7.07-7.03 (m, 1H), 7.02 (d, *J* = 4.4 Hz, 1H), 6.99 (d, *J* = 8.8 Hz, 2H), 6.82 (d, *J* = 8.8 Hz, 2H), 6.05 (d, *J* = 7.6 Hz, 1H), 5.11 (s, 2H), 4.89 (s, 2H), 4.03 (t, *J* = 4.4 Hz, 2H), 3.78 (s, 3H), 3.62 (q, *J* = 5.2 Hz, 2H), 2.12 (s, 3H); ^{13}C NMR (100 MHz, $CDCl_3$) δ 173.0, 168.1, 161.0, 160.2 (dd, ^{1}J = 241.3 Hz, ^{4}J = 2.0 Hz), 159.7, 157.4 (dd, ^{1}J = 241.2 Hz, ^{4}J = 2.2 Hz), 146.1, 142.0, 139.4, 130.7, 129.9, 129.5, 127.1, 125.8 (d, ^{3}J = 7.9 Hz), 125.6 (d, ^{3}J = 7.9 Hz), 116.7 (d, ^{2}J = 23.9 Hz), 116.6 (d, ^{2}J = 23.9 Hz), 116.6, 116.3 (d, ^{3}J = 8.6 Hz), 116.0 (d, ^{2}J = 28.4 Hz), 115.9, 115.7 (d, ^{2}J = 29.4 Hz), 114.5, 113.8, 73.0, 63.3 (d, ^{4}J = 4.3 Hz), 55.3, 52.9, 40.3, 12.6; ESI-HRMS: m/z calcd for $C_{30}H_{29}F_2N_2O_5$ [M+H]$^+$: 535.2039; found: 535.2053.

2.4.17 4-((3,5-Difluorobenzyl)oxy)-*N*-(2-(3-((4-methoxybenzyl)oxy)-2-methyl-4-oxopyridin-1(4*H*)-yl)ethyl)benzamide (**7q**)

White solid, yield 66%, m.p. 130-132 ºC; ^{1}H NMR (400 MHz, $CDCl_3$) δ 9.27 (t, *J* = 5.6 Hz, 1H), 8.14 (d, *J* = 8.8 Hz, 2H), 7.30 (d, *J* = 7.2 Hz, 2H), 7.07 (d, *J* = 7.6 Hz, 1H), 7.01-6.97 (m, 4H), 6.87 (d, *J* = 8.4 Hz, 2H), 6.79 (t, *J* = 9.2 Hz, 1H), 6.07 (d, *J* = 7.6 Hz, 1H), 5.08 (s, 2H), 4.92 (s, 2H), 4.07 (t, *J* = 4.8 Hz, 2H), 3.83 (s, 3H), 3.67 (q, *J* = 5.2 Hz, 2H), 2.17 (s, 3H); ^{13}C NMR (100 MHz, $CDCl_3$) δ 173.0, 168.1, 164.6 (dd, ^{1}J = 247.9 Hz, ^{3}J = 12.5 Hz), 160.9, 159.8, 146.1, 142.0, 140.7 (t, ^{3}J = 9.0 Hz), 139.43, 130.7, 129.9, 129.5, 127.2, 116.6, 114.6, 113.8, 110.0 (dd, ^{2}J = 25.8 Hz, ^{3}J = 7.2 Hz), 103.5 (t, ^{2}J = 25.2 Hz), 73.0, 68.7, 55.4, 52.9, 40.3, 12.6; ESI-HRMS: m/z calcd for $C_{30}H_{29}F_2N_2O_5$ [M+H]$^+$: 535.2039; found: 535.2021.

2.4.18 *N*-(2-(3-((4-methoxybenzyl)oxy)-2-methyl-4-oxopyridin-1(4*H*)-yl)ethyl)-4-((4-(trifluoromethyl)benzyl)oxy)benzamide (**7r**)

White solid, yield 62%, m.p. 141-143 ºC; ^{1}H NMR (400 MHz, $CDCl_3$) δ 9.12 (t, J = 5.6 Hz, 1H), 8.08 (d, J = 8.4 Hz, 2H), 7.63 (d, J = 8.0 Hz, 2H), 7.52 (d, J = 8.0 Hz, 2H), 7.25 (d, J = 5.6 Hz, 2H), 7.03 (d, J = 7.6 Hz, 1H), 6.97 (d, J = 8.8 Hz, 2H), 6.82 (d, J = 8.4 Hz, 2H), 6.05 (d, J = 7.2 Hz, 1H), 5.12 (s, 2H), 4.88 (s, 2H), 4.03 (t, J = 4.4 Hz, 2H), 3.78 (s, 3H), 3.62 (q, J = 5.2 Hz, 2H), 2.12 (s, 3H); ^{13}C NMR (100 MHz, $CDCl_3$) δ 173.0, 168.1, 161.2, 159.8, 146.1, 142.0, 140.7, 139.5, 130.7, 130.6 (q, ^{2}J = 32.3 Hz), 129.9, 129.5, 127.5, 127.1, 125.7 (q, ^{3}J = 3.7 Hz), 125.5 (d, ^{1}J = 270.6 Hz), 116.6, 114.6, 113.9, 73.1, 69.2, 55.4, 52.9, 40.3, 12.6; ESI-HRMS: m/z calcd for $C_{31}H_{30}F_3N_2O_5$ $[M+H]^+$: 567.2101; found: 567.2114.

2.4.19 *N*-(2-(3-((4-methoxybenzyl)oxy)-2-methyl-4-oxopyridin-1(4*H*)-yl)ethyl)-4-((3-methylbenzyl)oxy)benzamide (**7s**)

White solid, yield 57%, m.p. 121-123 ºC; ^{1}H NMR (400 MHz, $CDCl_3$) δ 9.02 (d, J = 5.6 Hz, 1H), 8.06 (d, J = 8.4 Hz, 2H), 7.28 (d, J = 3.2 Hz, 2H), 7.25 (d, J = 2.4 Hz, 1H), 7.23-7.18 (m, 2H), 7.14 (d, J = 7.6 Hz, 1H), 7.03 (d, J = 7.6 Hz, 1H), 6.99 (d, J = 8.4 Hz, 2H), 6.82 (d, J = 8.0 Hz, 2H), 6.05 (d, J = 7.2 Hz, 1H), 5.03 (s, 2H), 4.90 (s, 2H), 4.03 (t, J = 4.4 Hz, 2H), 3.78 (s, 3H), 3.62 (q, J = 5.2 Hz, 2H), 2.36 (s, 3H), 2.12 (s, 3H); ^{13}C NMR (100 MHz, $CDCl_3$) δ 173.0, 168.3, 161.7, 159.7, 146.1, 141.9, 139.4, 138.5, 136.5, 130.7, 129.7, 129.6, 129.0, 128.7, 128.4, 126.6, 124.7, 116.6, 114.6, 113.8, 73.0, 70.3, 55.4, 52.9, 40.3, 21.5, 12.6; ESI-HRMS: m/z calcd for $C_{31}H_{33}N_2O_5$ $[M+H]^+$: 513.2384; found: 513.2391.

2.4.20 *N*-(2-(3-((4-methoxybenzyl)oxy)-2-methyl-4-oxopyridin-1(4*H*)-yl)ethyl)-4-((4-methylbenzyl)oxy)benzamide (**7t**)

White solid, yield 70%, m.p. 115-117 ºC; ^{1}H NMR (400 MHz, $CDCl_3$) δ 9.03-8.87 (m, 1H), 8.02 (dd, J = 9.2, 2.8 Hz, 2H), 7.29 (d, J = 8.0 Hz, 2H), 7.25 (d, J = 4.4 Hz, 2H), 7.18 (d, J = 7.6 Hz, 2H), 7.04 (d, J = 7.2 Hz, 1H), 6.97 (d, J = 8.0 Hz, 2H), 6.81 (d, J = 8.0 Hz, 2H), 6.06 (dd, J = 7.2, 3.6 Hz, 1H), 5.02 (s, 2H), 4.90 (d, J = 3.6 Hz, 2H), 4.02 (t, J = 5.2 Hz, 2H), 3.78 (s, 3H), 3.61 (q, J = 5.2 Hz, 2H), 2.35 (s, 3H), 2.11 (s, 3H); ^{13}C NMR (100 MHz, $CDCl_3$) δ 173.1, 168.3, 161.7, 159.7, 146.1, 141.9, 139.4, 138.1, 133.5, 130.7, 129.7, 129.6, 129.4, 127.8, 126.5, 116.6, 114.7, 113.8, 73.0, 70.1, 55.4, 52.84, 40.3, 21.3, 12.6; ESI-HRMS: m/z calcd for $C_{31}H_{33}N_2O_5$ $[M+H]^+$: 513.2384; found: 513.2347.

2.4.21 4-((4-Isopropylbenzyl)oxy)-*N*-(2-(3-((4-methoxybenzyl)oxy)-2-methyl-4-oxopyridin-1(4*H*)-yl)ethyl)benzamide (**7u**)

White solid, yield 72%, m.p. 120-122 ºC; ^{1}H NMR (400 MHz, $CDCl_3$) δ 9.00 (d, J = 5.6 Hz, 1H), 8.05 (d, J = 8.8 Hz, 2H), 7.34 (d, J = 8.0 Hz, 2H), 7.27 (d, J = 5.2 Hz, 2H), 7.23 (d, J = 8.4 Hz, 2H), 7.04 (d, J = 7.6 Hz, 1H), 6.99 (d, J = 8.8 Hz, 2H), 6.82 (d, J = 8.4 Hz, 2H), 6.06 (d, J = 7.6 Hz, 1H), 5.03 (s, 2H), 4.90 (s, 2H), 4.03 (t, J = 4.4 Hz, 2H), 3.78 (s, 3H), 3.62 (q, J = 5.6 Hz, 2H), 2.97-2.84 (m, 1H), 2.12 (s, 3H), 1.26 (s, 3H), 1.24 (s, 3H); ^{13}C NMR (100 MHz, $CDCl_3$) δ 173.0, 168.3, 161.8, 159.7, 149.1, 146.1, 141.9, 139.4, 133.9, 130.7, 129.7, 129.6, 127.9, 126.9, 126.5, 116.6, 114.6, 113.8, 73.0, 70.2, 55.4, 52.9, 40.3, 34.0, 24.1, 12.7; ESI-HRMS: m/z calcd for $C_{33}H_{37}N_2O_5$ $[M+H]^+$: 541.2697; found: 541.2710.

2.4.22 4-((4-(*tert*-Butyl)benzyl)oxy)-*N*-(2-(3-((4-methoxybenzyl)oxy)-2-methyl-4-oxopyridin-1(4*H*)-yl)ethyl)benzamide (**7v**)

White solid, yield 74%, m.p. 140-142 ºC; ^{1}H NMR (400 MHz, $CDCl_3$) δ 8.97 (d, J = 19.6 Hz, 1H), 8.05 (d, J = 8.4 Hz, 2H), 7.41 (d, J = 8.0 Hz, 2H), 7.35 (d, J = 8.0 Hz, 2H), 7.27 (d, J = 6.8 Hz, 2H), 7.04 (d, J = 7.2 Hz, 1H), 6.99 (d, J = 8.4 Hz, 2H), 6.82 (d, J = 8.4 Hz, 2H), 6.06 (d, J = 7.6 Hz, 1H), 5.04 (s, 2H), 4.91 (s, 2H), 4.03 (t, J = 5.2 Hz, 2H), 3.78 (s, 3H), 3.62 (q, J = 5.2 Hz,

2H), 2.12 (s, 3H), 1.32 (s, 9H); ^{13}C NMR (100 MHz, $CDCl_3$) δ 173.0, 168.3, 161.8, 159.8, 151.4, 146.1, 141.9, 139.4, 133.5, 130.8, 129.7, 129.6, 127.6, 126.5, 125.7, 116.7, 114.6, 113.8, 73.0, 70.1, 55.4, 52.9, 40.3, 34.7, 31.5, 12.7; ESI-HRMS: m/z calcd for $C_{34}H_{39}N_2O_5$ $[M+H]^+$: 555.2853; found: 555.2865.

2.4.23 *N*-(2-(3-((4-methoxybenzyl)oxy)-2-methyl-4-oxopyridin-1(4*H*)-yl)ethyl) picolinamide (**10a**)

White solid, yield 88%, m.p. 126-128 °C; ^{1}H NMR (400 MHz, $CDCl_3$) δ 8.51 (d, *J* = 4.4 Hz, 1H), 8.29 (t, *J* = 6.4 Hz, 1H), 8.14 (d, *J* = 8.0 Hz, 1H), 7.85 (t, *J* = 7.2 Hz, 1H), 7.44 (dd, *J* = 6.4, 4.8 Hz, 1H), 7.30 (d, *J* = 8.4 Hz, 2H), 7.17 (d, *J* = 7.6 Hz, 1H), 6.83 (d, *J* = 8.4 Hz, 2H), 6.34 (d, *J* = 7.6 Hz, 1H), 5.12 (s, 2H), 4.02 (t, *J* = 6.4 Hz, 2H), 3.77 (s, 3H), 3.62 (q, *J* = 6.4 Hz, 2H), 2.13 (s, 3H); ^{13}C NMR (100 MHz, $CDCl_3$) δ 173.7, 165.4, 159.6, 149.1, 148.5, 146.2, 140.9, 138.6, 137.6, 130.9, 129.9, 126.8, 122.3, 117.5, 113.7, 72.7, 55.4, 52.3, 39.8, 12.6; ESI-HRMS: m/z calcd for $C_{22}H_{24}N_3O_4$ $[M+H]^+$: 394.1761; found: 394.1756.

2.4.24 *N*-(2-(3-((4-methoxybenzyl)oxy)-2-methyl-4-oxopyridin-1(4*H*)-yl)ethyl) nicotinamide (**10b**)

White solid, yield 93%, m.p. 105-107 °C; ^{1}H NMR (400 MHz, $CDCl_3$) δ 9.87 (t, *J* = 6.0 Hz, 1H), 9.30 (s, 1H), 8.68 (d, *J* = 4.0 Hz, 1H), 8.45 (d, *J* = 8.0 Hz, 1H), 7.35 (dd, *J* = 8.0, 4.8 Hz, 1H), 7.23 (d, *J* = 8.4 Hz, 2H), 7.03 (d, *J* = 7.6 Hz, 1H), 6.82 (d, *J* = 8.4 Hz, 2H), 6.05 (d, *J* = 7.2 Hz, 1H), 4.83 (s, 2H), 4.01 (t, *J* = 4.4 Hz, 2H), 3.78 (s, 3H), 3.62 (q, *J* = 5.2 Hz, 2H), 2.11 (s, 3H); ^{13}C NMR (100 MHz, $CDCl_3$) δ 172.9, 167.0, 159.8, 152.3, 149.6, 146.1, 142.1, 139.6, 135.7, 130.7, 129.7, 129.2, 123.4, 116.5, 113.9, 73.1, 55.4, 53.0, 40.0, 12.6; ESI-HRMS: m/z calcd for $C_{22}H_{24}N_3O_4$ $[M+H]^+$: 394.1761; found: 394.1758.

2.4.25 *N*-(2-(3-((4-methoxybenzyl)oxy)-2-methyl-4-oxopyridin-1(4*H*)-yl)ethyl) isonicotinamide (**10c**)

White solid, yield 95%, m.p. 154-156 °C; ^{1}H NMR (400 MHz, $CDCl_3$) δ 10.00 (t, *J* = 5.2 Hz, 1H), 8.72 (d, *J* = 4.0 Hz, 2H), 8.03 (d, *J* = 4.0 Hz, 2H), 7.23 (d, *J* = 7.2 Hz, 2H), 7.01 (d, *J* = 7.2 Hz, 1H), 6.82 (d, *J* = 7.2 Hz, 2H), 6.05 (d, *J* = 7.6 Hz, 1H), 4.82 (s, 2H), 4.02 (t, *J* = 4.6 Hz, 2H), 3.78 (s, 3H), 3.62 (q, J = 5.2 Hz, 2H), 2.12 (s, 3H); ^{13}C NMR (100 MHz, $CDCl_3$) δ 172.9, 166.8, 159.9, 150.6, 146.1, 142.2, 141.0, 139.6, 130.7, 129.2, 121.9, 116.5, 113.9, 73.2, 55.4, 52.9, 40.2, 12.6; ESI-HRMS: m/z calcd for $C_{22}H_{24}N_3O_4$ $[M+H]^+$: 394.1761; found: 394.1754.

2.5 General synthetic procedures for 8a-y and 11a-c

A solution of **7a-v** or **10a-c** (0.5 mmol) in anhydrous DCM (15 mL) was cooled to -78 °C under nitrogen and BCl_3 (1 M in DCM, 1-1.5 mL) was slowly added dropwise. The mixture was stirred from -78 °C to room temperature for 12 h and then quenched with methanol (15 mL). The solvent was removed under vacuum and the residue was purified by recrystallization from methanol/ether to afford white solids **8a-v** and **11a-c**.

A solution of **7b-d** (0.5 mmol) in anhydrous DCM (15 mL) was cooled to -48 °C under nitrogen and BBr_3 (0.376 g, 1.5 mmol) was slowly added dropwise. The mixture was stirred from -48 °C to room temperature for 12 h and then quenched with methanol (15 mL). The solvent was removed under vacuum and the residue was purified by recrystallization from methanol/ether to afford white solids **8w-y**.

2.5.1 *N*-(2-(3-hydroxy-2-methyl-4-oxopyridin-1(4*H*)-yl)ethyl)benzamide (**8a**)

White solid, yield 97%, m.p. 211-213 °C; ^{1}H NMR (400 MHz, DMSO-d_6) δ 8.81 (t, *J* = 6.0

Hz, 1H), 8.13 (d, *J* = 6.8 Hz, 1H), 7.77 (d, *J* = 7.2 Hz, 2H), 7.53 (t, *J* = 7.2 Hz, 1H), 7.46 (t, *J* = 7.6 Hz, 2H), 7.24 (d, *J* = 6.8 Hz, 1H), 4.52 (t, *J* = 5.6 Hz, 2H), 3.69 (q, *J* = 6.0 Hz, 2H), 2.58 (s, 3H); ^{13}C NMR (100 MHz, DMSO-d_6) δ 166.8, 158.8, 142.9, 141.7, 138.7, 133.7, 131.5, 128.4, 127.1, 110.4, 55.3, 38.6, 12.6; ESI-HRMS: m/z calcd for $C_{15}H_{17}N_2O_3$ [M+H]$^+$: 273.1234; found: 273.1228.

2.5.2 *N*-(2-(3-hydroxy-2-methyl-4-oxopyridin-1(4*H*)-yl)ethyl)-2-methoxy benzamide (**8b**)

White solid, yield 93%, m.p. 130-132 °C; ^{1}H NMR (400 MHz, DMSO-d_6) δ 8.38 (t, *J* = 5.6 Hz, 1H), 8.11 (t, *J* = 6.2 Hz, 1H), 7.64 (d, *J* = 7.6 Hz, 1H), 7.49 (t, *J* = 7.6 Hz, 1H), 7.28 (dd, *J* = 24.0, 6.8 Hz, 1H), 7.15 (d, *J* = 8.4 Hz, 1H), 7.03 (t, *J* = 7.2 Hz, 1H), 4.54 (t, *J* = 5.2 Hz, 2H), 3.86 (s, 3H), 3.73 (q, *J* = 5.6 Hz, 2H), 2.61 (s, 3H); ^{13}C NMR (100 MHz, DMSO-d_6) δ 165.7, 158.9, 156.9, 142.8, 141.7, 138.7, 132.4, 130.2, 122.6, 120.4, 112.0, 110.4, 55.8, 55.2, 38.7, 12.6; ESI-HRMS: m/z calcd for $C_{16}H_{19}N_2O_4$ [M+H]$^+$: 303.1339; found: 303.1336.

2.5.3 *N*-(2-(3-hydroxy-2-methyl-4-oxopyridin-1(4*H*)-yl)ethyl)-3-methoxy benzamide (**8c**)

White solid, yield 96%, m.p. 218-220 °C; ^{1}H NMR (400 MHz, DMSO-d_6) δ 8.86 (d, *J* = 9.2 Hz,1H), 8.16 (t, *J* = 7.2 Hz, 1H), 7.39-7.36 (m, 2H), 7.33-7.26 (m, 2H), 7.09 (dd, *J* = 4.4, 1.8 Hz, 1H), 4.53 (t, *J* = 5.6 Hz, 2H), 3.79 (s, 3H), 3.68 (q, *J* = 5.6 Hz, 2H), 2.58 (s, 3H); ^{13}C NMR (100 MHz, DMSO-d_6) δ 166.5, 159.2, 158.8, 142.9, 141.8, 138.7, 135.2, 129.5, 119.3, 117.3, 112.4, 110.4, 55.3, 55.2, 38.6, 12.6; ESI-HRMS: m/z calcd for $C_{16}H_{19}N_2O_4$ [M+H]$^+$: 303.1339; found: 303.1336.

2.5.4 *N*-(2-(3-hydroxy-2-methyl-4-oxopyridin-1(4*H*)-yl)ethyl)-4-methoxy benzamide (**8d**)

White solid, yield 91%, m.p. 241-243 °C; ^{1}H NMR (400 MHz, DMSO-d_6) δ 8.71 (t, *J* = 5.6 Hz, 1H), 8.14 (d, *J* = 6.8 Hz, 1H), 7.77 (d, *J* = 8.8 Hz, 2H), 7.27 (d, *J* = 6.8 Hz, 1H), 6.98 (d, *J* = 8.8 Hz, 2H), 4.52 (t, *J* = 6.0 Hz, 2H), 3.80 (s, 3H), 3.67 (q, *J* = 6.0 Hz, 2H), 2.57 (s, 3H); ^{13}C NMR (100 MHz, DMSO-d_6) δ 166.2, 161.7, 158.7, 142.8, 141.8, 138.7, 129.0, 125.9, 113.6, 110.4, 55.4, 55.3, 38.6, 12.6; ESI-HRMS: m/z calcd for $C_{16}H_{19}N_2O_4$ [M+H]$^+$: 303.1339; found: 303.1343.

2.5.5 2-(Benzyloxy)-*N*-(2-(3-hydroxy-2-methyl-4-oxopyridin-1(4*H*)-yl)ethyl) benzamide (**8e**)

White solid, yield 78%, m.p. 180-182 °C; ^{1}H NMR (400 MHz, DMSO-d_6) δ 8.43 (t, *J* = 6.0 Hz, 1H), 7.98 (d, *J* = 6.8 Hz, 1H), 7.57 (d, *J* = 7.6 Hz, 1H), 7.45-7.37 (m, 5H), 7.32 (t, *J* = 6.8 Hz, 1H), 7.17 (d, *J* = 6.4 Hz, 1H), 7.14 (d, *J* = 8.4 Hz, 1H), 7.00 (t, *J* = 7.2 Hz, 1H), 5.26 (s, 2H), 4.46 (t, *J* = 6.0 Hz, 2H), 3.69 (q, *J* = 6.0 Hz, 2H), 2.55 (s, 3H); ^{13}C NMR (100 MHz, DMSO-d_6) δ 166.0, 158.9, 155.6, 142.9, 141.5, 138.5, 136.7, 132.1, 130.0, 128.5, 127.9, 127.3, 123.5, 120.7, 113.5, 110.4, 69.7, 55.2, 38.7, 12.5; ESI-HRMS: m/z calcd for $C_{22}H_{23}N_2O_4$ [M+H]$^+$: 379.1652; found: 379.1673.

2.5.6 3-(Benzyloxy)-*N*-(2-(3-hydroxy-2-methyl-4-oxopyridin-1(4*H*)-yl)ethyl) benzamide (**8f**)

White solid, yield 97%, m.p. 178-180 °C; ^{1}H NMR (400 MHz, DMSO-d_6) δ 8.88 (t, *J* = 5.6 Hz, 1H), 8.15 (d, *J* = 7.2 Hz, 1H), 7.46 (d, *J* = 7.6 Hz, 3H), 7.42-7.31 (m, 5H), 7.28 (d, *J* = 7.2 Hz, 1H), 7.19-7.15 (m, 1H), 5.15 (s, 2H), 4.53 (t, *J* = 5.6 Hz, 2H), 3.69 (q, *J* = 6.0 Hz, 2H), 2.58 (s, 3H); ^{13}C NMR (100 MHz, DMSO-d_6) δ 166.5, 158.8, 158.3, 142.9, 141.7, 138.7, 136.8, 135.2, 129.6, 128.4, 127.9, 127.8, 119.6, 118.0, 113.4, 110.4, 69.4, 55.2, 38.6, 12.6; ESI-HRMS: m/z calcd for $C_{22}H_{23}N_2O_4$ [M+H]$^+$: 379.1652; found: 379.1672.

2.5.7 4-(Benzyloxy)-*N*-(2-(3-hydroxy-2-methyl-4-oxopyridin-1(4*H*)-yl)ethyl) benzamide (**8g**)

White solid, yield 89%, m.p. 246-248 °C; ^{1}H NMR (400 MHz, DMSO-d_6) δ 8.68 (t, *J* = 5.6 Hz, 1H), 8.12 (d, *J* = 7.2 Hz, 1H), 7.76 (d, *J* = 8.4 Hz, 2H), 7.45 (d, *J* = 6.8 Hz, 2H), 7.40 (t, *J* =

7.2 Hz, 2H), 7.33 (t, *J* = 7.0 Hz, 1H), 7.25 (d, *J* = 7.2 Hz, 1H), 7.07 (d, *J* = 8.8 Hz, 2H), 5.16 (s, 2H), 4.51 (t, *J* = 5.6 Hz, 2H), 3.66 (q, *J* = 5.6 Hz, 2H), 2.57 (s, 3H); ^{13}C NMR (100 MHz, DMSO-d_6) δ 166.2, 160.8, 158.8, 142.9, 141.7, 138.7, 136.7, 129.0, 128.5, 128.0, 127.8, 126.1, 114.4, 110.4, 69.4, 55.3, 38.6, 12.6; ESI-HRMS: m/z calcd for $C_{22}H_{23}N_2O_4$ $[M+H]^+$: 379.1652; found: 379.1648.

2.5.8 4-Ethoxy-*N*-(2-(3-hydroxy-2-methyl-4-oxopyridin-1(4*H*)-yl)ethyl)benzamide (**8h**)

White solid, yield 93%, m.p. 246-248 °C; ^{1}H NMR (400 MHz, DMSO-d_6) δ 8.70 (t, *J* = 5.2 Hz, 1H), 8.14 (d, *J* = 6.8 Hz, 1H), 7.75 (d, *J* = 8.4 Hz, 2H), 7.28 (d, *J* = 6.8 Hz, 1H), 6.96 (d, *J* = 8.4 Hz, 2H), 4.52 (t, *J* = 6.0 Hz, 2H), 4.07 (q, *J* = 7.2 Hz, 2H), 3.66 (q, *J* = 6.0 Hz, 2H), 2.57 (s, 3H), 1.32 (t, *J* = 6.8 Hz, 3H); ^{13}C NMR (100 MHz, DMSO-d_6) δ 166.2, 161.0, 158.7, 142.8, 141.7, 138.7, 129.0, 125.7, 114.0, 110.4, 63.3, 55.3, 38.6, 14.5, 12.6; ESI-HRMS: m/z calcd for $C_{17}H_{21}N_2O_4$ $[M+H]^+$: 317.1496; found: 317.1499.

2.5.9 *N*-(2-(3-hydroxy-2-methyl-4-oxopyridin-1(4*H*)-yl)ethyl)-4-propoxybenzamide (**8i**)

White solid, yield 97%, m.p. 214-243 °C; ^{1}H NMR (400 MHz, DMSO-d_6) δ 8.67 (t, *J* = 5.6 Hz, 1H), 8.13 (d, *J* = 6.8 Hz, 1H), 7.75 (d, *J* = 8.8 Hz, 2H), 7.26 (d, *J* = 6.8 Hz, 1H), 6.97 (d, *J* = 8.4 Hz, 2H), 4.51 (t, *J* = 6.0 Hz, 2H), 3.97 (t, *J* = 6.4 Hz, 2H), 3.66 (q, *J* = 6.0 Hz, 2H), 2.57 (s, 3H), 1.78-1.68 (m, 2H), 0.97 (t, *J* = 7.6 Hz, 3H); ^{13}C NMR (100 MHz, DMSO-d_6) δ 166.2, 161.2, 158.8, 142.8, 141.7, 138.7, 129.0, 125.7, 114.0, 110.4, 69.1, 55.3, 38.6, 21.9, 12.6, 10.3; ESI-HRMS: m/z calcd for $C_{18}H_{23}N_2O_4$ $[M+H]^+$: 331.1652; found: 331.1664.

2.5.10 *N*-(2-(3-hydroxy-2-methyl-4-oxopyridin-1(4*H*)-yl)ethyl)-4-(prop-2-yn- 1-yloxy)benzamide (**8j**)

White solid, yield 85%, m.p. 208-210 °C; ^{1}H NMR (400 MHz, DMSO-d_6) δ 8.73 (t, *J* = 6.0 Hz, 1H), 8.14 (d, *J* = 6.8 Hz, 1H), 7.77 (d, *J* = 8.8 Hz, 2H), 7.27 (d, *J* = 8.0 Hz, 1H), 7.03 (d, *J* = 8.8 Hz, 2H), 4.86 (s, 2H), 4.52 (t, *J* = 6.0 Hz, 2H), 3.67 (q, *J* = 6.0 Hz, 2H), 3.61 (s, 1H), 2.57 (s, 3H); ^{13}C NMR (100 MHz, DMSO-d_6) δ 166.2, 159.6, 158.8, 142.8, 141.7, 138.7, 128.9, 126.6, 114.4, 110.4, 78.9, 78.5, 55.6, 55.3, 38.6, 12.6; ESI-HRMS: m/z calcd for $C_{18}H_{19}N_2O_4$ $[M+H]^+$: 327.1339; found: 327.1355.

2.5.11 4-(Cyclohexylmethoxy)-*N*-(2-(3-hydroxy-2-methyl-4-oxopyridin-1(4*H*)-yl) ethyl)benzamide (**8k**)

White solid, yield 95%, m.p. 274-276 °C; ^{1}H NMR (400 MHz, DMSO-d_6) δ 8.66 (t, *J* = 5.6 Hz, 1H), 8.12 (d, *J* = 7.2 Hz, 1H), 7.74 (d, *J* = 8.8 Hz, 2H), 7.26 (d, *J* = 6.8 Hz, 1H), 6.96 (d, *J* = 8.4 Hz, 2H), 4.51 (t, *J* = 5.2 Hz, 2H), 3.82 (d, *J* = 6.4 Hz, 2H), 3.66 (q, *J* = 6.0 Hz, 2H), 2.57 (s, 3H), 1.79 (d, *J* = 12.8 Hz, 2H), 1.70 (d, *J* = 12.8 Hz, 3H), 1.64 (d, *J* = 11.6 Hz, 1H), 1.22 (h, *J* = 12.0 Hz, 3H), 1.03 (q, *J* = 11.2 Hz, 2H); ^{13}C NMR (100 MHz, DMSO-d_6) δ 166.2, 161.4, 158.8, 142.8, 141.7, 138.6, 129.0, 125.7, 114.0, 110.4, 72.8, 55.3, 38.6, 37.0, 29.2, 26.0, 25.2, 12.6; ESI-HRMS: m/z calcd for $C_{22}H_{29}N_2O_4$ $[M+H]^+$: 385.2122; found: 385.2135.

2.5.12 4-((3-Fluorobenzyl)oxy)-*N*-(2-(3-hydroxy-2-methyl-4-oxopyridin-1(4*H*)-yl) ethyl)benzamide (**8l**)

White solid, yield 91%, m.p. 236-238 °C; ^{1}H NMR (400 MHz, DMSO-d_6) δ 8.66 (t, *J* = 3.2 Hz, 1H), 8.12 (d, *J* = 6.8 Hz, 1H), 7.76 (d, *J* = 8.4 Hz, 2H), 7.44 (td, *J* = 8.0, 6.0 Hz, 1H), 7.30 (s, 1H), 7.28 (d, *J* = 4.4 Hz, 1H), 7.26-7.14 (m, 1H), 7.17 (td, *J* = 8.4, 2.4 Hz, 1H), 7.07 (d, *J* = 8.4 Hz, 2H), 5.19 (s, 2H), 4.51 (t, *J* = 6.0 Hz, 2H), 3.66 (q, *J* = 5.6 Hz, 2H), 2.57 (s, 3H); ^{13}C NMR (100 MHz, DMSO-d_6) δ 166.2, 163.4 (d, ^1J = 242.4 Hz), 160.6, 158.7, 142.8, 141.7, 139.7 (d, ^3J = 7.3 Hz), 138.7, 130.5 (d, ^3J = 8.2 Hz), 129.0, 126.3, 123.6 (d, ^4J = 2.8 Hz), 114.8 (d, ^2J = 20.7 Hz),

114.4, 114.2, 110.4, 68.5, 55.3, 38.6, 12.6; ESI-HRMS: m/z calcd for $C_{22}H_{22}FN_2O_4$ $[M+H]^+$: 397.1558; found: 397.1555.

2.5.13 4-((4-Fluorobenzyl)oxy)-*N*-(2-(3-hydroxy-2-methyl-4-oxopyridin-1(4*H*)-yl) ethyl)benzamide (**8m**)

White solid, yield 95%, m.p. 254-256 °C; ^{1}H NMR (400 MHz, DMSO-*d$_6$*) δ 8.72 (t, J = 5.6 Hz, 1H), 8.14 (d, J = 6.8 Hz, 1H), 7.77 (d, J = 8.8 Hz, 2H), 7.52 (d, J = 6.0 Hz, 1H), 7.50 (d, J = 5.6 Hz, 1H), 7.28 (d, J = 6.8 Hz, 1H), 7.22 (t, J = 8.8 Hz, 2H), 7.06 (d, J = 8.8 Hz, 2H), 5.14 (s, 2H), 4.52 (t, J = 6.0 Hz, 2H), 3.67 (q, J = 5.6 Hz, 2H), 2.57 (s, 3H); ^{13}C NMR (100 MHz, DMSO-*d$_6$*) δ 166.2, 163.0 (d, ^1J = 242.6 Hz), 160.7, 158.8, 142.8, 141.7, 138.7, 132.9 (d, ^4J = 3.1 Hz), 130.1 (d, ^3J = 8.3 Hz), 129.0, 126.2, 115.4 (d, ^2J = 21.3 Hz), 114.4, 110.4, 68.7, 55.3, 38.6, 12.6; ESI-HRMS: m/z calcd for $C_{22}H_{22}FN_2O_4$ $[M+H]^+$: 397.1558; found: 397.1531.

2.5.14 4-((3-Chlorobenzyl)oxy)-*N*-(2-(3-hydroxy-2-methyl-4-oxopyridin-1(4*H*)-yl) ethyl)benzamide (**8n**)

White solid, yield 95%, m.p. 236-238 °C; ^{1}H NMR (400 MHz, DMSO-*d$_6$*) δ 8.72 (t, J = 5.6 Hz, 1H), 8.14 (d, J = 6.8 Hz, 1H), 7.77 (d, J = 8.4 Hz, 2H), 7.52 (s, 1H), 7.44-7.39 (m, 3H), 7.28 (d, J = 6.8 Hz, 1H), 7.07 (d, J = 8.4 Hz, 2H), 5.18 (s, 2H), 4.52 (t, J = 5.2 Hz, 2H), 3.67 (q, J = 6.0 Hz, 2H), 2.57 (s, 3H); ^{13}C NMR (100 MHz, DMSO-*d$_6$*) δ 166.2, 160.6, 158.8, 142.8, 141.7, 139.3, 138.7, 133.1, 130.4, 129.0, 127.9, 127.4, 126.3, 126.3, 114.4, 110.4, 68.4, 55.3, 38.6, 12.6; ESI-HRMS: m/z calcd for $C_{22}H_{22}ClN_2O_4$ $[M+H]^+$: 413.1263; found: 413.1240.

2.5.15 4-((4-Chlorobenzyl)oxy)-*N*-(2-(3-hydroxy-2-methyl-4-oxopyridin-1(4*H*)-yl) ethyl)benzamide (**8o**)

White solid, yield 93%, m.p. 256-258 °C; ^{1}H NMR (400 MHz, DMSO-*d$_6$*) δ 8.80 (t, J = 5.6 Hz, 1H), 8.16 (d, J = 6.4 Hz, 1H), 7.78 (d, J = 8.4 Hz,2H), 7.48 (d, J = 8.4 Hz, 2H), 7.45 (d, J = 8.4 Hz, 2H), 7.31 (d, J = 6.4 Hz, 1H), 7.05 (d, J = 8.4 Hz, 2H), 5.16 (s, 2H), 4.52 (t, J = 5.2 Hz, 2H), 3.67 (q, J = 5.6 Hz, 2H), 2.57 (s, 3H); ^{13}C NMR (100 MHz, DMSO-*d$_6$*) δ 166.1, 160.6, 158.8, 142.8, 141.6, 138.6, 135.7, 132.5, 129.6, 129.0, 128.5, 126.2, 114.4, 110.4, 68.5, 55.2, 38.6, 12.6; ESI-HRMS: m/z calcd for $C_{22}H_{22}ClN_2O_4$ $[M+H]^+$: 413.1263; found: 413.1228.

2.5.16 4-((2,5-Difluorobenzyl)oxy)-*N*-(2-(3-hydroxy-2-methyl-4-oxopyridin-1(4*H*) -yl)ethyl)benzamide (**8p**)

White solid, yield 96%, m.p. 236-238 °C; ^{1}H NMR (400 MHz, DMSO-*d$_6$*) δ 8.73 (t, J = 5.6 Hz, 1H), 8.14 (d, J = 6.8 Hz, 1H), 7.79 (d, J = 8.8 Hz, 2H), 7.46-7.40 (m, 1H), 7.36-7.30 (m, 1H), 7.30-7.24 (m, 2H), 7.10 (d, J = 8.8 Hz, 2H), 5.19 (s, 2H), 4.52 (t, J = 5.6 Hz, 2H), 3.67 (q, J = 5.6 Hz, 2H), 2.58 (s, 3H); ^{13}C NMR (100 MHz, DMSO-*d$_6$*) δ 166.2, 160.4, 159.3 (dd, ^1J = 239.2 Hz, ^4J = 1.5 Hz), 158.8, 157.6 (dd, ^1J = 241.0 Hz, ^4J = 2.0 Hz), 142.9, 141.7, 138.7, 129.1, 126.5, 125.6 (d, ^3J = 8.1 Hz), 125.5 (d, ^3J = 8.2 Hz), 117.2 (d, ^2J = 24.2 Hz), 117.1 (d, ^2J = 24.2 Hz), 116.9, 116.8 (d, ^2J = 23.7 Hz), 116.7 (d, ^4J = 3.7 Hz), 114.4, 110.4, 63.3, 55.3, 38.6, 12.6; ESI-HRMS: m/z calcd for $C_{22}H_{21}F_2N_2O_4$ $[M+H]^+$: 415.1464; found: 415.1419.

2.5.17 4-((3,5-Difluorobenzyl)oxy)-*N*-(2-(3-hydroxy-2-methyl-4-oxopyridin-1(4*H*) -yl)ethyl)benzamide (**8q**)

White solid, yield 88%, m.p. 250-252 °C; ^{1}H NMR (400 MHz, DMSO-*d$_6$*) δ 8.74 (t, J = 5.2 Hz, 1H), 8.14 (d, J = 6.8 Hz, 1H), 7.78 (d, J = 8.8 Hz, 2H), 7.28 (d, J = 7.2 Hz, 1H), 7.22-7.17 (m, 3H), 7.07 (d, J = 8.8 Hz, 2H), 5.20 (s, 2H), 4.52 (t, J = 5.6 Hz, 2H), 3.67 (q, J = 6.0 Hz, 2H), 2.57 (s, 3H); ^{13}C NMR (100 MHz, DMSO-*d$_6$*) δ 166.2, 163.7 (dd, ^1J = 245.0 Hz, ^3J = 13.2 Hz), 160.4, 158.8, 142.8, 141.7, 141.4 (t, ^3J = 9.4 Hz), 138.7, 129.1, 126.5, 114.4, 110.6, 110.5 (dd, ^2J = 25.4

Hz, ^{3}J = 6.2 Hz), 103.3 (t, ^{2}J = 25.5 Hz), 68.0, 55.3, 38.6, 12.6; ESI-HRMS: m/z calcd for $C_{22}H_{21}F_{2}N_{2}O_{4}$ [M+H]$^+$: 415.1464; found: 415.1463.

2.5.18 *N*-(2-(3-hydroxy-2-methyl-4-oxopyridin-1(4*H*)-yl)ethyl)-4-((4-(trifluoro methyl)benzyl)oxy)benzamide (**8r**)

White solid, yield 98%, m.p. 268-270 °C; ^{1}H NMR (400 MHz, DMSO-*d6*) δ 8.73 (t, *J* = 5.2 Hz, 1H), 8.14 (d, *J* = 6.8 Hz, 1H), 7.78 (d, *J* = 6.8 Hz, 2H), 7.76 (d, *J* = 5.6 Hz, 2H), 7.67 (d, *J* = 8.0 Hz, 2H), 7.28 (d, *J* = 7.2 Hz, 1H), 7.08 (d, *J* = 8.4 Hz, 2H), 5.29 (s, 2H), 4.52 (t, *J* = 6.0 Hz, 2H), 3.67 (q, *J* = 5.6 Hz, 2H), 2.57 (s, 3H); ^{13}C NMR (100 MHz, DMSO-*d6*) δ 166.2, 160.5, 158.8, 142.8, 141.7, 141.6, 138.7, 129.1, 128.56 (d, ^{2}J = 31.6 Hz), 128.1, 126.4, 125.6 (d, ^{1}J = 270.5 Hz), 125.3 (q, ^{3}J = 4.0 Hz), 114.4, 110.4, 68.4, 55.3, 38.6, 12.6; ESI-HRMS: m/z calcd for $C_{23}H_{22}F_{3}N_{2}O_{4}$ [M+H]$^+$: 447.1526; found: 447.1488.

2.5.19 *N*-(2-(3-hydroxy-2-methyl-4-oxopyridin-1(4*H*)-yl)ethyl)-4-((3-methylbenzyl) oxy)benzamide (**8s**)

White solid, yield 94%, m.p. 239-241 °C; ^{1}H NMR (400 MHz, DMSO-*d6*) δ 8.73-8.65 (m, 1H), 8.15-8.11 (m, 1H), 7.76 (d, *J* = 6.8 Hz, 2H), 7.30-7.22 (m, 4H), 7.14 (d, *J* = 7.2 Hz, 1H), 7.06 (d, *J* = 8.4 Hz, 2H), 5.11 (s, 2H), 4.51 (t, *J* = 5.6 Hz, 2H), 3.67 (q, *J* = 5.6 Hz, 2H), 2.57 (s, 3H), 2.31 (s, 3H); ^{13}C NMR (100 MHz, DMSO-*d6*) δ 166.2, 160.9, 158.8, 142.8, 141.7, 138.7, 137.6, 136.6, 129.0, 128.6, 128.4, 128.3, 126.1, 124.9, 114.4, 110.4, 69.4, 55.3, 38.6, 21.0, 12.6; ESI-HRMS: m/z calcd for $C_{23}H_{25}N_{2}O_{4}$ [M+H]$^+$: 393.1809; found: 393.1772.

2.5.20 *N*-(2-(3-hydroxy-2-methyl-4-oxopyridin-1(4*H*)-yl)ethyl)-4-((4-methylbenzyl) oxy)benzamide (**8t**)

White solid, yield 80%, m.p. 245-247 °C; ^{1}H NMR (400 MHz, DMSO-*d6*) δ 8.67 (t, *J* = 6.8 Hz, 1H), 8.10 (d, *J* = 6.8 Hz, 1H), 7.74 (d, *J* = 8.8 Hz, 2H), 7.33 (d, *J* = 8.0 Hz, 2H), 7.23 (s, 1H), 7.20 (d, *J* = 8.0 Hz, 2H), 7.04 (d, *J* = 8.8 Hz, 2H), 5.10 (s, 2H), 4.50 (t, *J* = 5.6 Hz, 2H), 3.66 (q, *J* = 6.0 Hz, 2H), 2.56 (s, 3H), 2.30 (s, 3H); ^{13}C NMR (100 MHz, DMSO-*d6*) δ 166.2, 160.8, 158.8, 142.8, 141.6, 138.6, 137.2, 133.6, 129.0, 129.0, 127.9, 126.0, 114.4, 110.4, 69.3, 55.2, 38.6, 20.8, 12.6; ESI-HRMS: m/z calcd for $C_{23}H_{25}N_{2}O_{4}$ [M+H]$^+$: 393.1809; found: 393.1792.

2.5.21 *N*-(2-(3-hydroxy-2-methyl-4-oxopyridin-1(4*H*)-yl)ethyl)-4-((4-isopropyl benzyl)oxy)benzamide (**8u**)

White solid, yield 88%, m.p. 247-249 °C; ^{1}H NMR (400 MHz, DMSO-*d6*) δ 8.63 (t, *J* = 4.8 Hz, 1H), 8.10 (d, *J* = 6.8 Hz, 1H), 7.74 (d, *J* = 8.8 Hz, 2H), 7.37 (d, *J* = 8.0 Hz, 2H), 7.26 (d, *J* = 8.0 Hz, 2H), 7.22 (d, *J* = 6.8 Hz, 1H), 7.06 (d, *J* = 8.4 Hz, 2H), 5.11 (s, 2H), 4.50 (t, *J* = 5.6 Hz, 2H), 3.66 (q, *J* = 5.6 Hz, 2H), 2.89 (hept, *J* = 6.8 Hz, 1H), 2.57 (s, 3H), 1.21 (s, 3H), 1.19 (s, 3H); ^{13}C NMR (100 MHz, DMSO-*d6*) δ 166.2, 160.9, 158.8, 148.2, 142.9, 141.6, 138.7, 134.0, 129.0, 128.0, 126.3, 126.0, 114.4, 110.4, 69.3, 55.3, 38.6, 33.2, 23.8, 12.6; ESI-HRMS: m/z calcd for $C_{25}H_{29}N_{2}O_{4}$ [M+H]$^+$: 421.2122; found: 421.2080.

2.5.22 4-((4-(*tert*-Butyl)benzyl)oxy)-*N*-(2-(3-hydroxy-2-methyl-4-oxopyridin-1(4*H*) -yl)ethyl)benzamide (**8v**)

White solid, yield 83%, m.p. 245-247 °C; ^{1}H NMR (400 MHz, DMSO-*d6*) δ 8.72 (t, *J* = 6.0 Hz, 1H), 8.14 (d, *J* = 6.8 Hz, 1H), 7.77 (d, *J* = 8.4 Hz, 2H), 7.41 (d, *J* = 8.4 Hz, 2H), 7.37 (d, *J* = 8.0 Hz, 2H), 7.27 (d, *J* = 6.8 Hz, 1H), 7.05 (d, *J* = 8.4 Hz, 2H), 5.11 (s, 2H), 4.52 (t, *J* = 6.0 Hz, 2H), 3.67 (q, *J* = 5.6 Hz, 2H), 2.57 (s, 3H), 1.27 (s, 9H); ^{13}C NMR (100 MHz, DMSO-*d6*) δ 166.2, 160.9, 158.8, 150.4, 142.8, 141.7, 138.7, 133.6, 129.0, 127.7, 126.0, 125.2, 114.4, 110.4, 69.2, 55.3, 38.6, 34.3, 31.1, 12.6; ESI-HRMS: m/z calcd for $C_{26}H_{31}N_{2}O_{4}$ [M+H]$^+$: 435.2278; found:

435.2242.

2.5.23 2-Hydroxy-*N*-(2-(3-hydroxy-2-methyl-4-oxopyridin-1(4*H*)-yl)ethyl) benzamide (**8w**)

White solid, yield 80%, m.p. 156-158 ºC; ^{1}H NMR (400 MHz, DMSO-*d$_6$*) *δ* 11.93 (s, 1H), 8.92 (t, *J* = 6.4 Hz, 1H), 8.12-8.08 (m, 1H), 7.73 (d, *J* = 6.8 Hz, 1H), 7.40 (t, *J* = 7.6 Hz, 1H), 7.06 (t, *J* = 6.8 Hz, 1H), 6.92-6.86 (m, 2H), 4.55 (t, *J* = 5.6 Hz, 2H), 3.73 (q, *J* = 6.0 Hz, 2H), 2.59 (s, 3H); ^{13}C NMR (100 MHz, DMSO-*d$_6$*) *δ* 169.0, 159.3, 158.7, 142.9, 141.9, 138.8, 133.8, 128.1, 118.8, 117.3, 115.4, 110.5, 55.2, 38.4, 12.6; ESI-HRMS: m/z calcd for $C_{15}H_{17}N_2O_4$ $[M+H]^+$: 289.1183; found: 289.1182.

2.5.24 3-Hydroxy-*N*-(2-(3-hydroxy-2-methyl-4-oxopyridin-1(4*H*)-yl)ethyl) benzamide (**8x**)

White solid, yield 93%, m.p. 144-146 ºC; ^{1}H NMR (400 MHz, DMSO-*d$_6$*) δ 9.68 (s, 1H), 8.57 (t, *J* = 5.6 Hz, 1H), 8.10 (d, *J* = 6.8 Hz, 1H), 7.23 (t, *J* = 8.0 Hz, 1H), 7.16 (d, *J* = 7.6 Hz, 1H), 7.13-7.09 (m, 2H), 6.92 (d, *J* = 8.0 Hz, 1H), 4.52 (t, *J* = 5.6 Hz, 2H), 3.66 (q, *J* = 5.6 Hz, 2H), 2.58 (s, 3H); ^{13}C NMR (100 MHz, DMSO-*d$_6$*) *δ* 166.9, 158.5, 157.4, 142.9, 142.0, 138.8, 135.2, 129.4, 118.4, 117.6, 114.1, 110.4, 55.4, 38.6, 12.7; ESI-HRMS: m/z calcd for $C_{15}H_{17}N_2O_4$ $[M+H]^+$: 289.1183; found: 289.1189.

2.5.25 4-Hydroxy-*N*-(2-(3-hydroxy-2-methyl-4-oxopyridin-1(4*H*)-yl)ethyl) benzamide (**8y**)

White solid, yield 89%, m.p. 163-165 ºC; ^{1}H NMR (400 MHz, DMSO-*d$_6$*) *δ* 10.03 (s, 1H), 8.45 (t, *J* = 5.2 Hz, 1H), 8.11 (d, *J* = 6.8 Hz, 1H), 7.63 (d, *J* = 8.4 Hz, 2H), 7.09 (d, *J* = 6.8 Hz, 1H), 6.79 (d, *J* = 8.4 Hz, 2H), 4.51 (t, *J* = 6.0 Hz, 2H), 3.65 (q, *J* = 6.0 Hz, 2H), 2.57 (s, 3H); ^{13}C NMR (100 MHz, DMSO-*d$_6$*) *δ* 166.5, 160.4, 158.5, 142.8, 142.0, 138.8, 129.7, 124.4, 114.9, 110.4, 55.5, 38.5, 12.6; ESI-HRMS: m/z calcd for $C_{15}H_{17}N_2O_4$ $[M+H]^+$: 289.1183; found: 289.1183.

2.5.26 *N*-(2-(3-hydroxy-2-methyl-4-oxopyridin-1(4*H*)-yl)ethyl)picolinamide (**11a**)

White solid, yield 97%, m.p. 219-221 ºC; ^{1}H NMR (400 MHz, DMSO-*d$_6$*) *δ* 9.12 (t, *J* = 5.6 Hz, 1H), 8.64 (d, *J* = 4.4 Hz, 1H), 8.c08 (d, *J* = 7.2 Hz, 1H), 8.02-7.96 (m, 2H), 7.62 (t, *J* = 5.2 Hz, 1H), 7.29 (d, *J* = 6.8 Hz, 1H), 4.54 (t, *J* = 5.6 Hz, 2H), 3.73 (q, *J* = 6.0 Hz, 2H), 2.57 (s, 3H); ^{13}C NMR (100 MHz, DMSO-*d$_6$*) *δ* 164.4, 158.6, 149.2, 148.4, 142.8, 142.1, 138.6, 138.1, 126.9, 122.1, 110.5, 55.4, 38.5, 12.7; ESI-HRMS: m/z calcd for $C_{14}H_{16}N_3O_3$ $[M+H]^+$: 274.1186; found: 274.1188.

2.5.27 *N*-(2-(3-hydroxy-2-methyl-4-oxopyridin-1(4*H*)-yl)ethyl)nicotinamide (**11b**)

White solid, yield 99%, m.p. 266-268 ºC; ^{1}H NMR (400 MHz, DMSO-*d$_6$*) *δ* 9.70 (t, *J* = 6.0 Hz, 1H), 9.22 (d, *J* = 2.0 Hz, 1H), 8.95 (dd, *J* = 5.6, 1.6 Hz, 1H), 8.72 (d, *J* = 8.0 Hz, 1H), 8.25 (d, *J* = 6.8 Hz, 1H), 7.96 (dd, *J* = 8.0, 5.2 Hz, 1H), 7.33 (d, *J* = 6.8 Hz, 1H), 4.58 (t, *J* = 5.6 Hz, 2H), 3.75 (q, *J* = 5.6 Hz, 2H), 2.60 (s, 3H); ^{13}C NMR (100 MHz, DMSO-*d$_6$*) *δ* 163.2, 158.7, 146.6, 143.5, 142.9, 141.9, 141.1, 138.8, 131.2, 126.0, 110.5, 55.1, 38.7, 12.7; ESI-HRMS: m/z calcd for $C_{14}H_{16}N_3O_3$ $[M+H]^+$: 274.1186; found: 274.1186.

2.5.28 *N*-(2-(3-hydroxy-2-methyl-4-oxopyridin-1(4*H*)-yl)ethyl)isonicotinamide (**11c**)

White solid, yield 93%, m.p. 280-282 ºC; ^{1}H NMR (400 MHz, DMSO-*d$_6$*) *δ* 9.84 (t, *J* = 5.6 Hz, 1H), 8.99 (d, *J* = 6.0 Hz, 2H), 8.26 (d, *J* = 7.2 Hz, 1H), 8.22 (d, *J* = 6.0 Hz, 2H), 7.33 (d, *J* = 6.8 Hz, 1H), 4.59 (t, *J* = 6.0 Hz, 2H), 3.76 (q, *J* = 6.0 Hz, 2H), 2.60 (s, 3H); ^{13}C NMR (100 MHz, DMSO-*d$_6$*) *δ* 163.5, 158.7, 146.0, 144.9, 142.9, 141.9, 138.8, 123.9, 110.5, 55.0, 38.8, 12.7; ESI-HRMS: m/z calcd for $C_{14}H_{16}N_3O_3$ $[M+H]^+$: 274.1186; found: 274.1187.

3. The determination of pK_a and iron (III) affinity

The pK_a and iron (III) affinity were determined by an automatic titration system based on

spectrophotometry (an autoburette, a Mettler Toledo pH meter and a luminescence 759s UV-Vis spectrophotometer) as previously described.[9] The test compound concentration was 20 mM in DMSO. Protonation equilibria was performed at KCl (0.1 M) over the pH range 2.0-10.5 in the 200-400 nm spectral range. Complex formation studies were performed at KCl (0.1 M):DMSO = 9:1 (*v*/*v*) (log β_1) over the pH range 0.9-2.1 and KCl (0.1 M):DMSO = 1.5:1 (*v*/*v*) (log β_2 and log β_3) over the pH range 2.0-9.5 in the 400-800 nm spectral range. The data were analyzed using HypSpec2014 program.[10,11]

4. Calculation of pFe^{3+}

The values of pFe^{3+} were calculated by HySS software according to the results of pK_{a1}, pK_{a2}, log β_1, log β_2, and log β_3. The basic parameters were set as follows: FeOH = -2.563, $Fe(OH)_2$ = -6.205, $Fe(OH)_3$ = -15.100, $Fe_2(OH)_2$ = -2.843, $Fe_3(OH)_4$ = -6.059, $Fe(OH)_4$ = -21.883.[12]

5. Human MAO inhibitory assay

Human MAO-A and MAO-B Inhibitor Screening Kit were purchased from Sigma-Aldrich and BioVision and stored at -20 °C. MAO-A and MAO-B enzyme was pre-aliquoted with 25 μL and 22 μL MAO-A/B Assay Buffer, respectively, and stored at -80 °C. This assay was performed according to the previously reported procedures.[9] In brief, testing inhibitors, inhibitor control or Assay Buffer (10 μL) were incubated with MAO-A/B enzyme solution (50 μL) at 37 °C for 10 min. Then substrate solution (40 μL) was added and quantified in a multi-detection microplate fluorescence reader (excitation, 535 nm; emission, 587 nm).

6. PAMPA-BBB assay

The PAMPA-BBB assay was performed to predict the BBB permeation of compound **8g**. The porcine brain lipid (PBL) was purchased from Avanti Polar Lipids. The donor microplates (PVDF membrane, pore size 0.45 μM) and the acceptor microplates were obtained from the Millipore. The filter surface of the donor microplate was first impregnated with 4 μL of porcine brain lipid (20 mg in 1 mL dodecane). The test compounds (5 mg/mL) were dissolved in DMSO and diluted to 25 μg/mL with PBS and 150 μL of the solution was filled in the donor well (V_d). The acceptor microplate was filled with 300 μL of phosphate buffer saline (PBS) (V_a). Then the donor microplate was carefully placed on the acceptor microplate to form a sandwich and incubated for 6 h at 25 °C. After incubation, the donor plate was removed and the concentration of the test compounds in the acceptor wells was determined using a multi-detection microplate fluorescence reader. Each sample was analyzed at maximum absorption wavelength, in eight wells, and at least in three independent runs. The standard concentration-absorbance curve for each compound was shown in **Table S1**. The results are expressed as mean ± SEM. P_e was calculated using the following equation:

$$P_e = \{C \times -\ln (1 - \frac{n_{acceptor}}{n_{total}})\}, \text{ where } C = (V_d \times \frac{V_a}{Area \times time \times (V_d + V_a)})$$

7. Molecular modeling studies

Molecular modeling was performed in Discovery Studio 4.0 software (version 4.0, BIOVIA,

USA) using the CDOCKER program. The crystal structure of MAO-B (PDB entry 2V5Z) with cocrystallized ligand (safinamide) and FAD co-factor was selected as the receptor model and all the parameters were set to their default values.[13] Chain B was deleted and all computations were performed in chain A. All ligands and other crystallized water molecules were deleted from the protein mode except the FAD co-factor and three highly conserved water molecules in the active site HOH 1155, 1170, 1351 (MAO-B, A-chain).

8. Intracellular ROS detection

Reactive Oxygen Species Assay Kit was purchased from KeyGEN BioTECH (KGT010-1) and stored at -20 °C. Neural pheochromocytoma-derived PC12 Cells (1×10^6 cell/ mL) were incubated for 24 h and further incubated with **8g** (10 μM) for 2 h, then $A\beta_{1-42}$ was added and incubated for 24 h. Cells were washed with PBS twice, incubated with DCFH-DA (10 μM) at 37 °C for 20 min, and then washed with PBS thrice. Fluorescence intensity was analyzed by flow cytometry (Becton-Dickinson FACS Calibur) (excitation, 488 nm; emission, 530 nm) and the fluorescence images were recorded by inverted biological microscope (Becton-Dickinson IX51).

9. Cognitive and memory assays *in vivo*

The adult female ICR mice (8-10 weeks old, weight 20-25 g) were obtained from the Zhejiang Academy of Medical Sciences (Hangzhou, China). Pargyline and Scopolamine hydrobromide were purchased from Aladdin Chemical Co., Ltd. (N159008, S107418). The test agents were prepared as clear injections, consisting of 10% DMSO, 20% (2-hydroxypropyl)-*β*-cyclodextrin and PBS. The mice were trained to find the platform (10 cm diameter) in the opaque circular pool (120 cm diameter, 60 cm height) filled with water (40 cm, depth), which was described in detail in our previous study.[1] All mice received at least one training session daily in four quadrants for four consecutive days before testing of finding the platform within 120 s and recording relevant data by the ANY-maze Video Tracking System.

The mice were randomly divided into four groups: (i) control group (PBS of 20% (2-hydroxypropyl)-*β*-cyclodextrin), (ii) scopolamine group (15 mg/kg), (iii) compound **8g** (15 mg/kg) + scopolamine group, (iv) pargyline (15 mg/kg) + scopolamine group. Mice in each group were intraperitoneally injected with indicated compounds or solvent 30 min before intraperitoneal injection with scopolamine or PBS once a day for 15 consecutive days. The Morris water maze test was performed at the last 5 days.

10. ^{1}H and ^{13}C NMR spectra of compounds 8 and 11

N-(2-(3-hydroxy-2-methyl-4-oxopyridin-1(4*H*)-yl)ethyl)benzamide (**8a**)

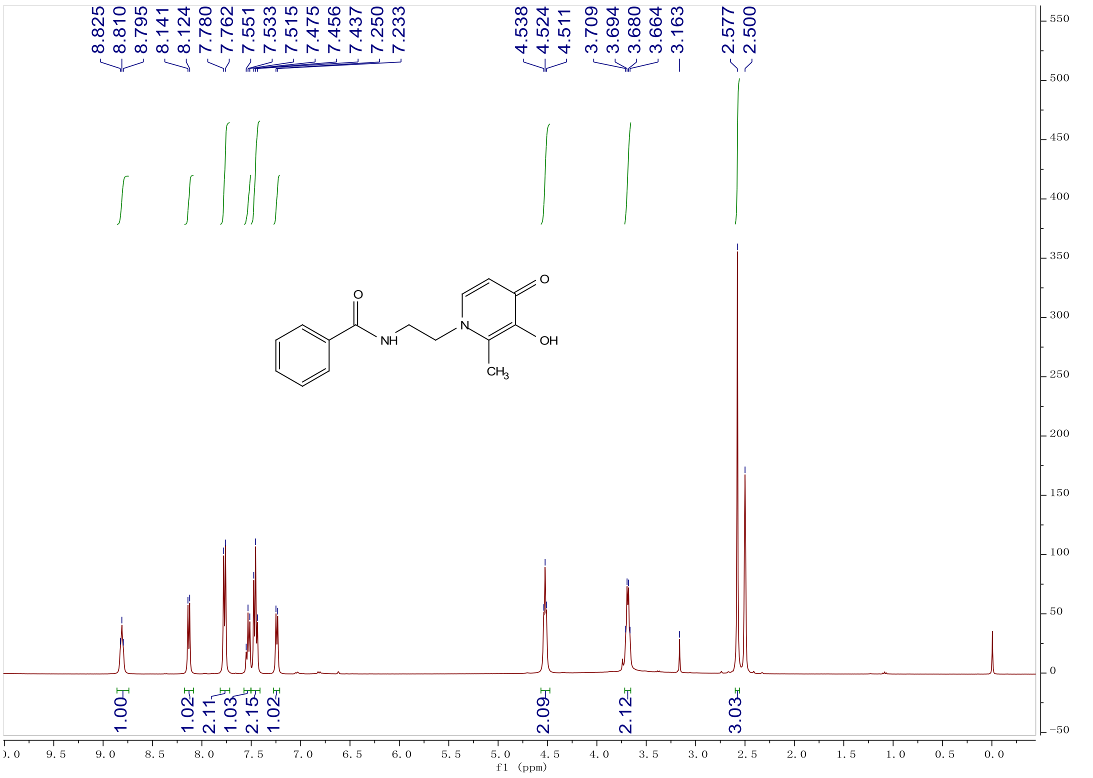

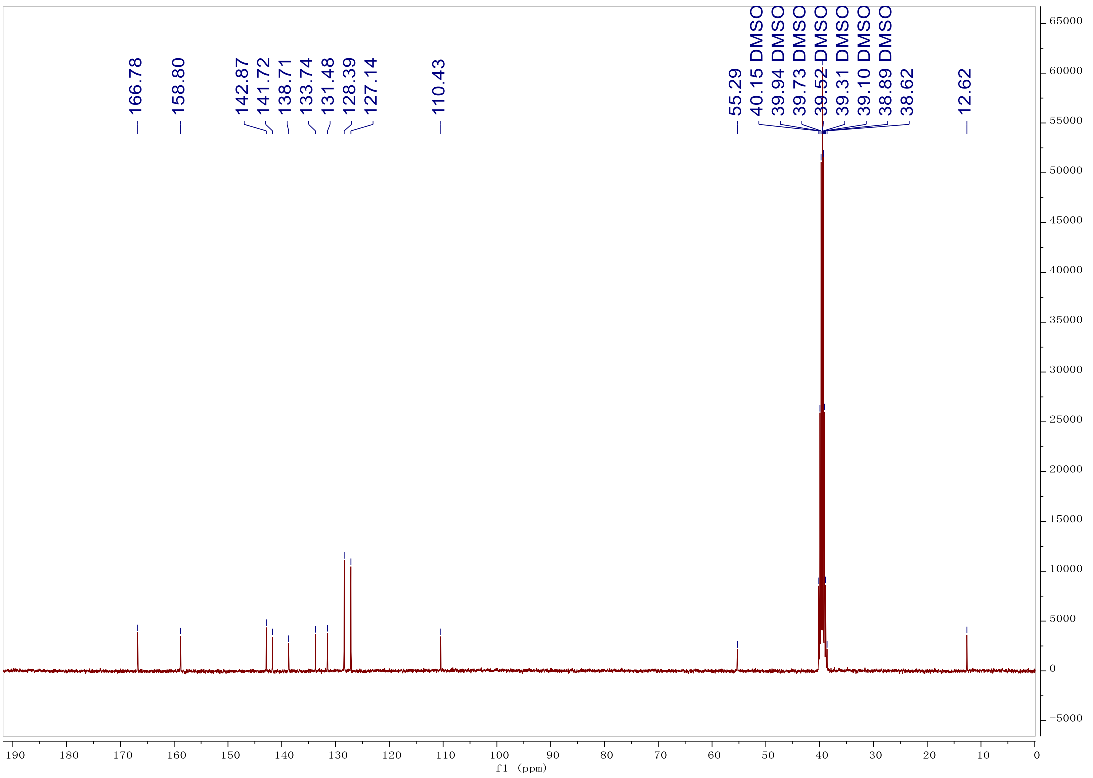

N-(2-(3-hydroxy-2-methyl-4-oxopyridin-1(4*H*)-yl)ethyl)-2-methoxy benzamide (**8b**)

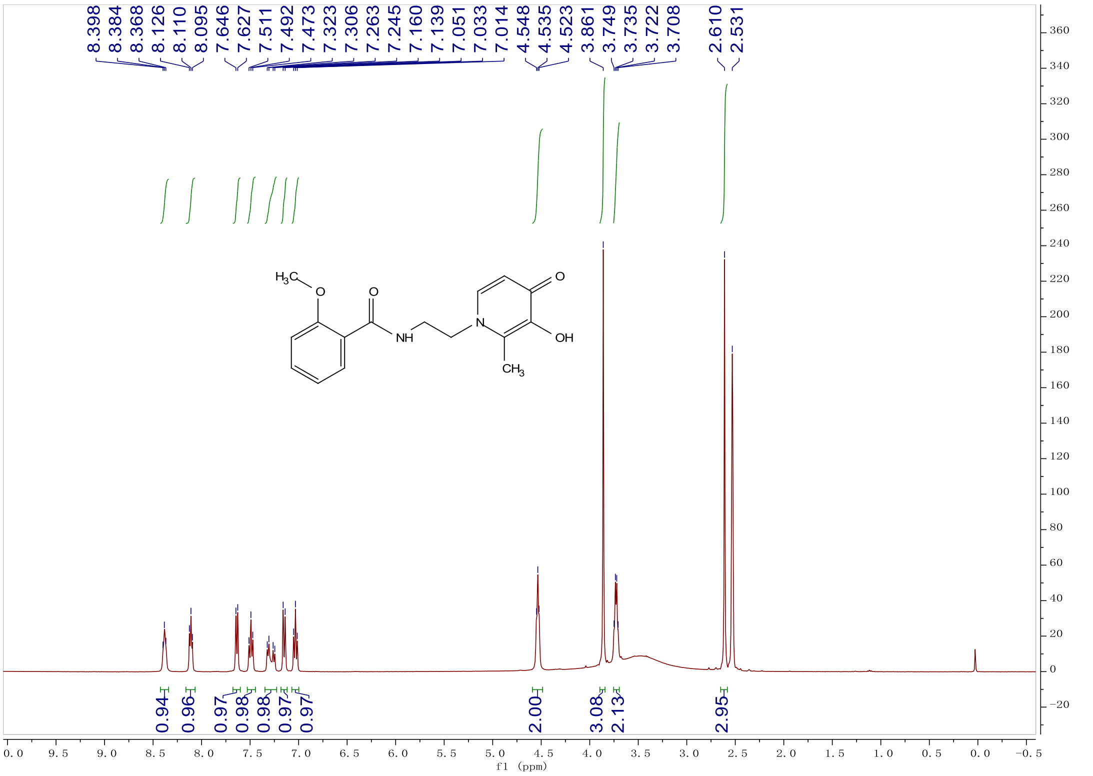

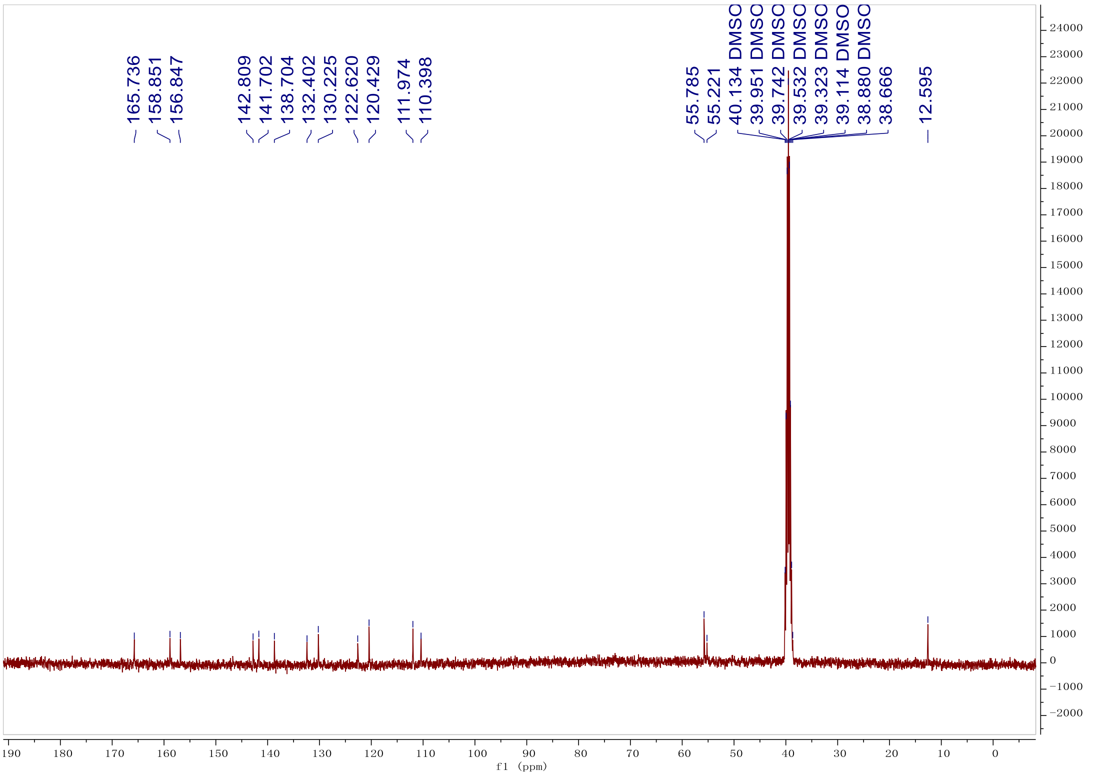

N-(2-(3-hydroxy-2-methyl-4-oxopyridin-1(4*H*)-yl)ethyl)-3-methoxy benzamide (**8c**)

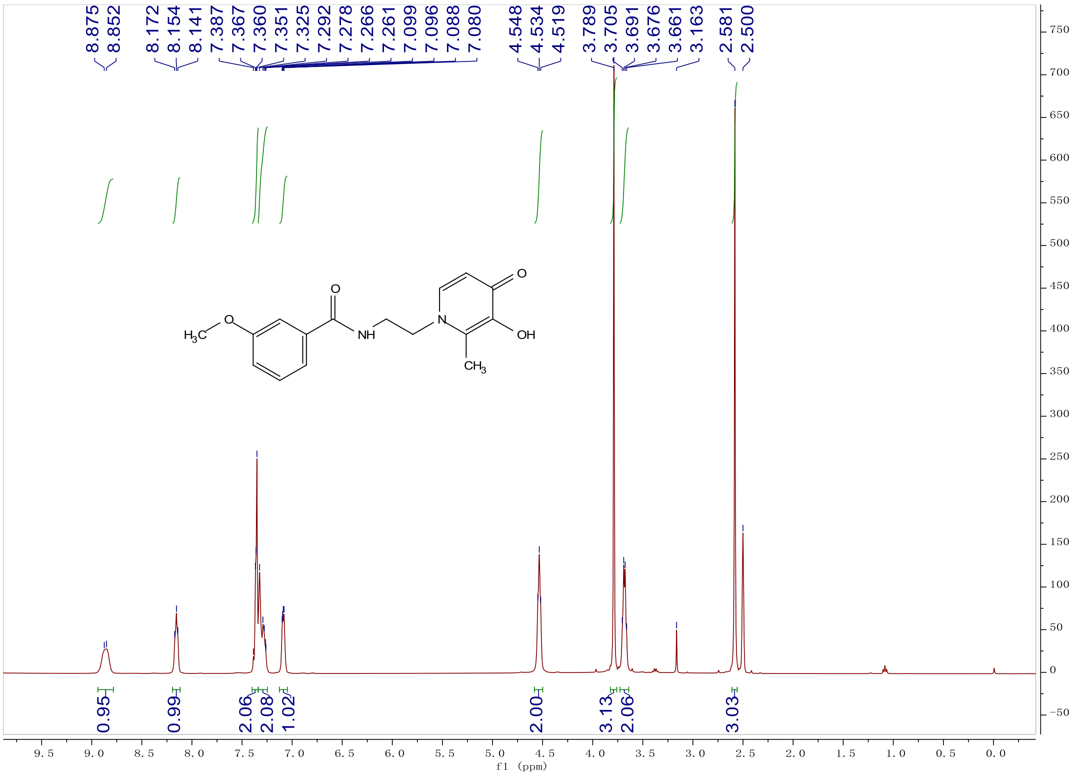

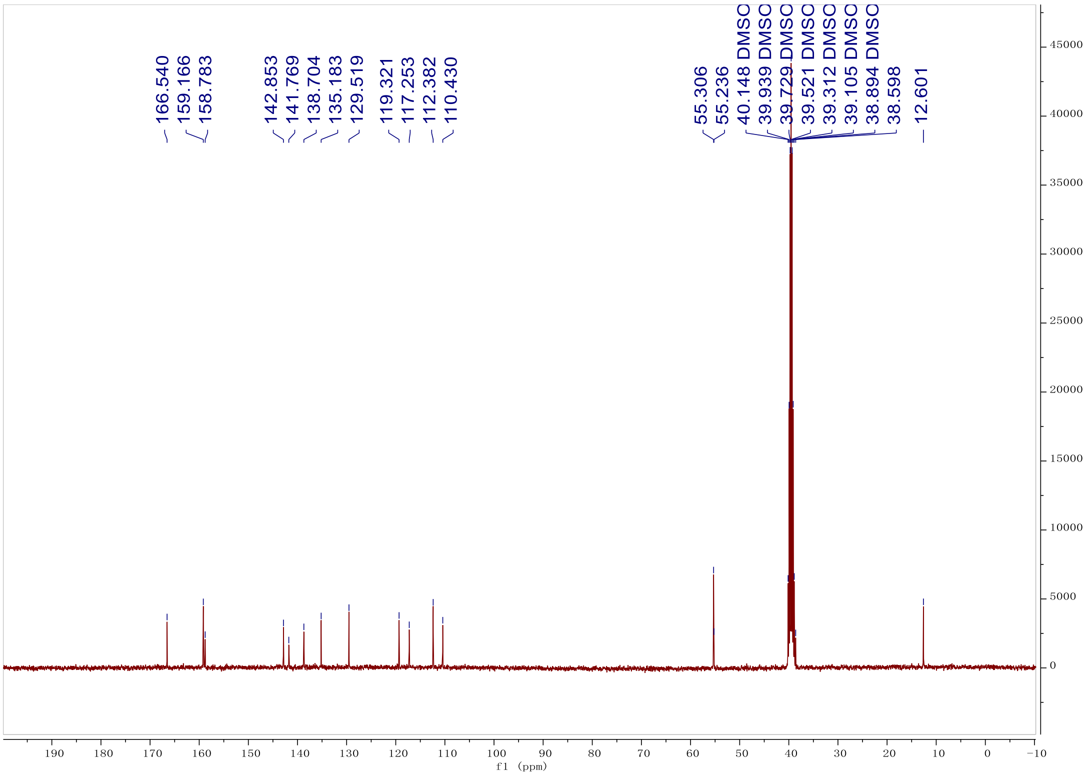

N-(2-(3-hydroxy-2-methyl-4-oxopyridin-1(4*H*)-yl)ethyl)-4-methoxy benzamide (**8d**)

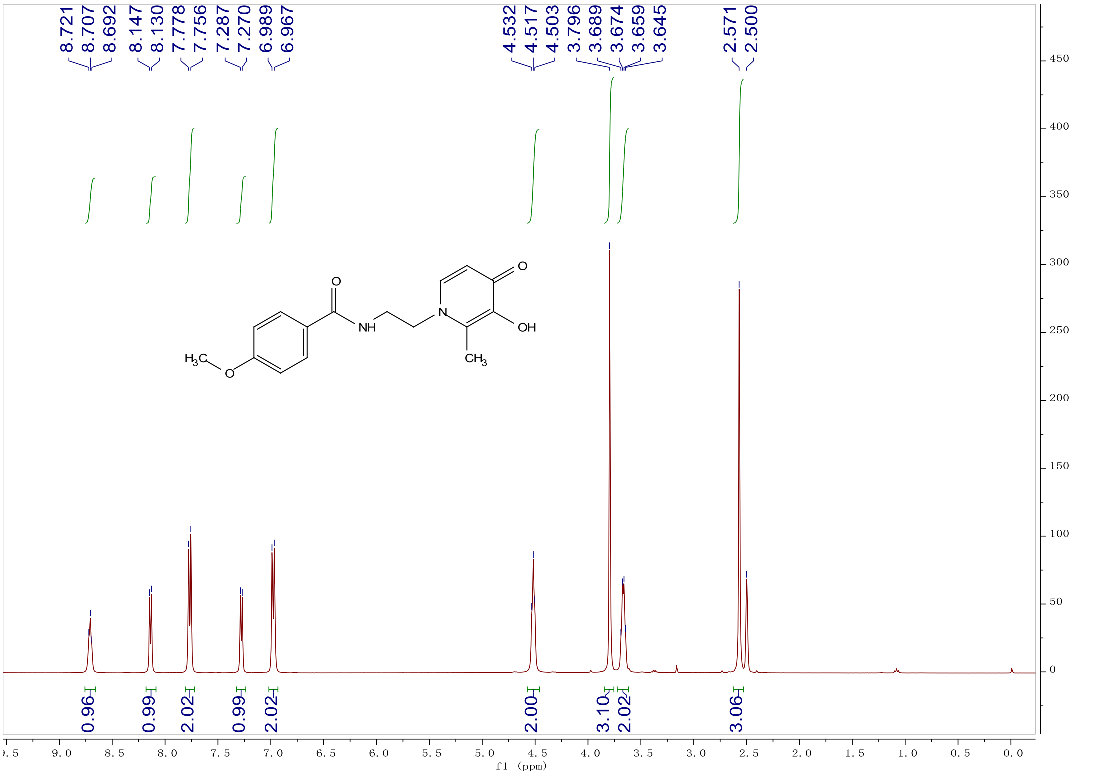

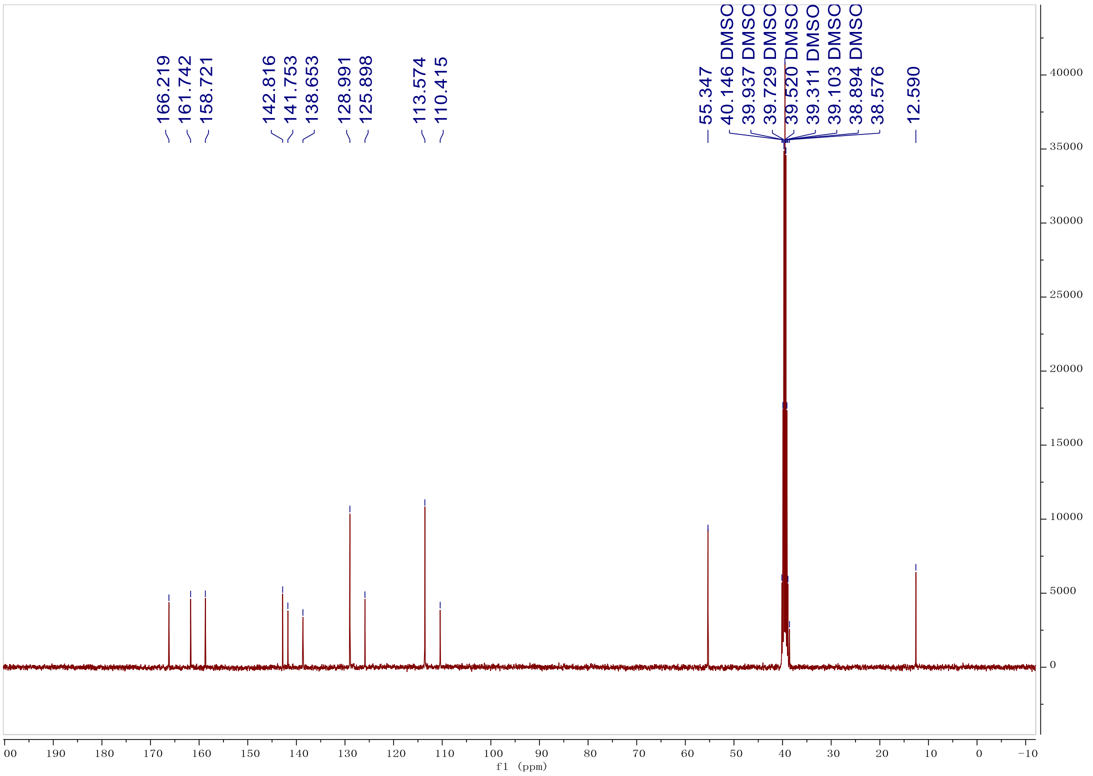

2-(Benzyloxy)-*N*-(2-(3-hydroxy-2-methyl-4-oxopyridin-1(4*H*)-yl)ethyl) benzamide (**8e**)

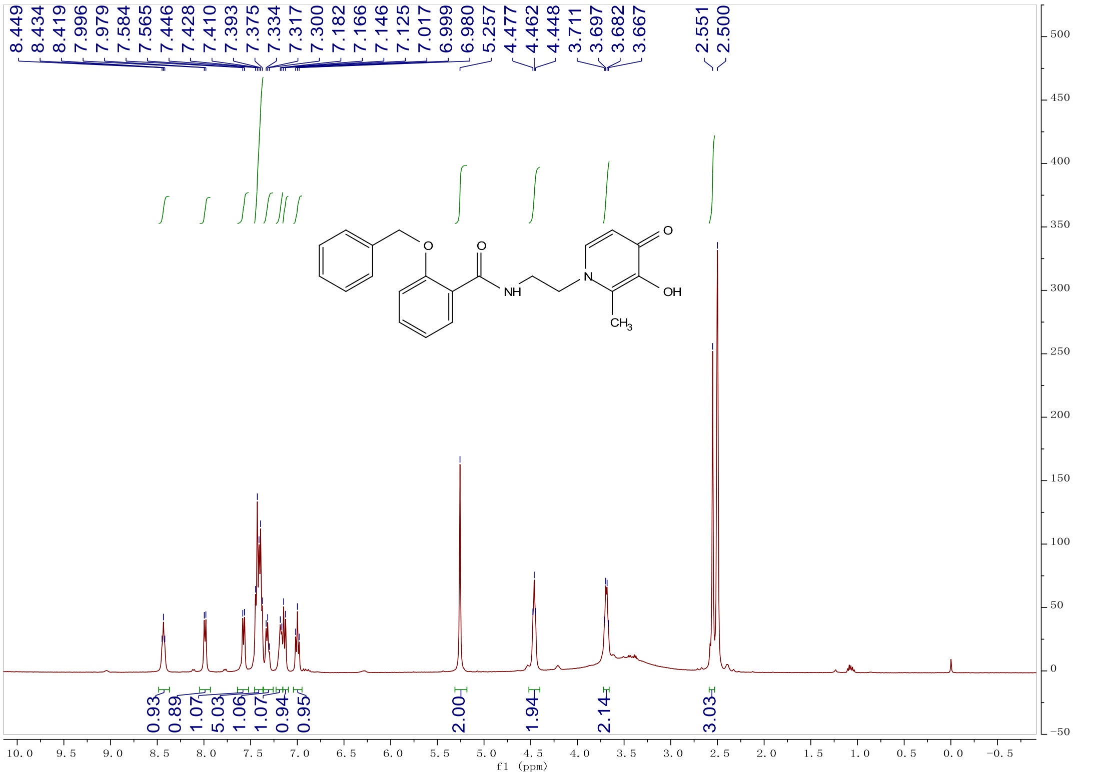

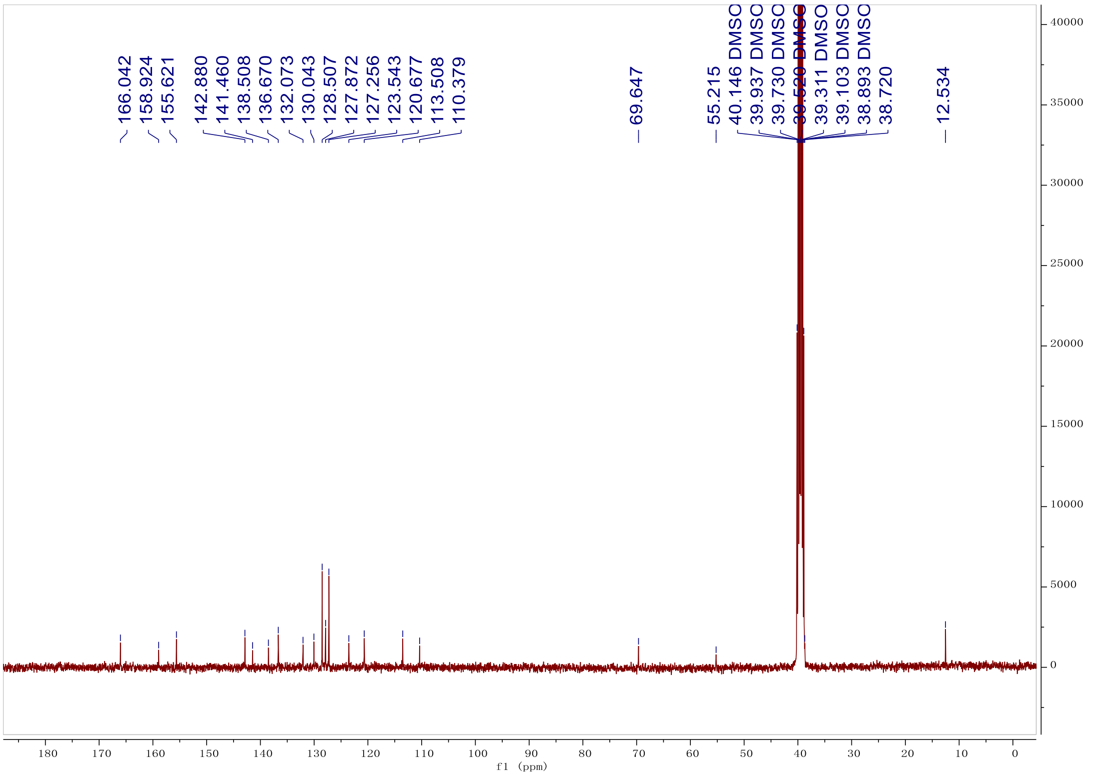

3-(Benzyloxy)-*N*-(2-(3-hydroxy-2-methyl-4-oxopyridin-1(4*H*)-yl)ethyl) benzamide (**8f**)

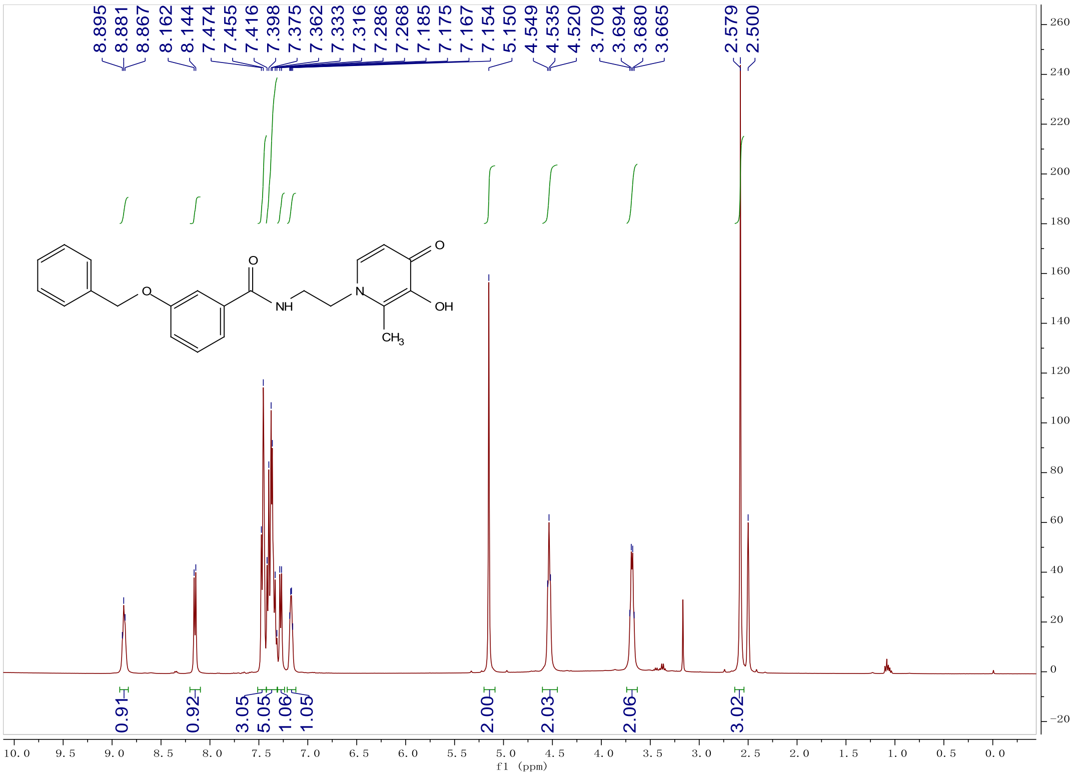

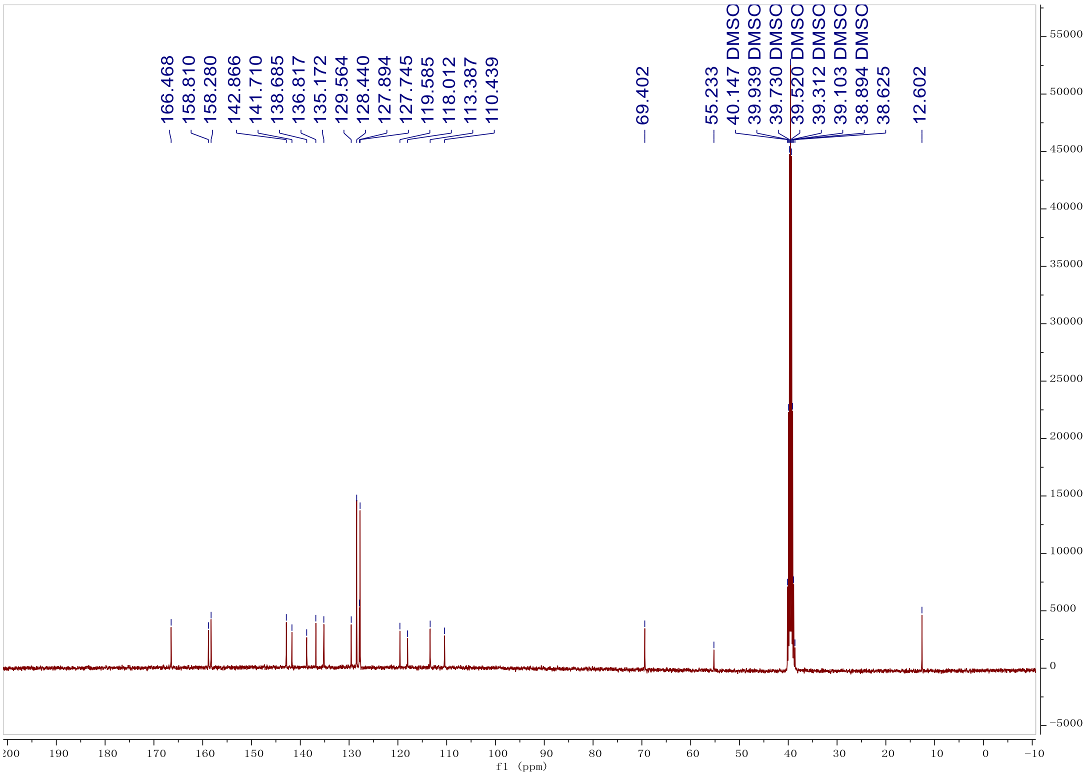

4-(Benzyloxy)-*N*-(2-(3-hydroxy-2-methyl-4-oxopyridin-1(4*H*)-yl)ethyl) benzamide (**8g**)

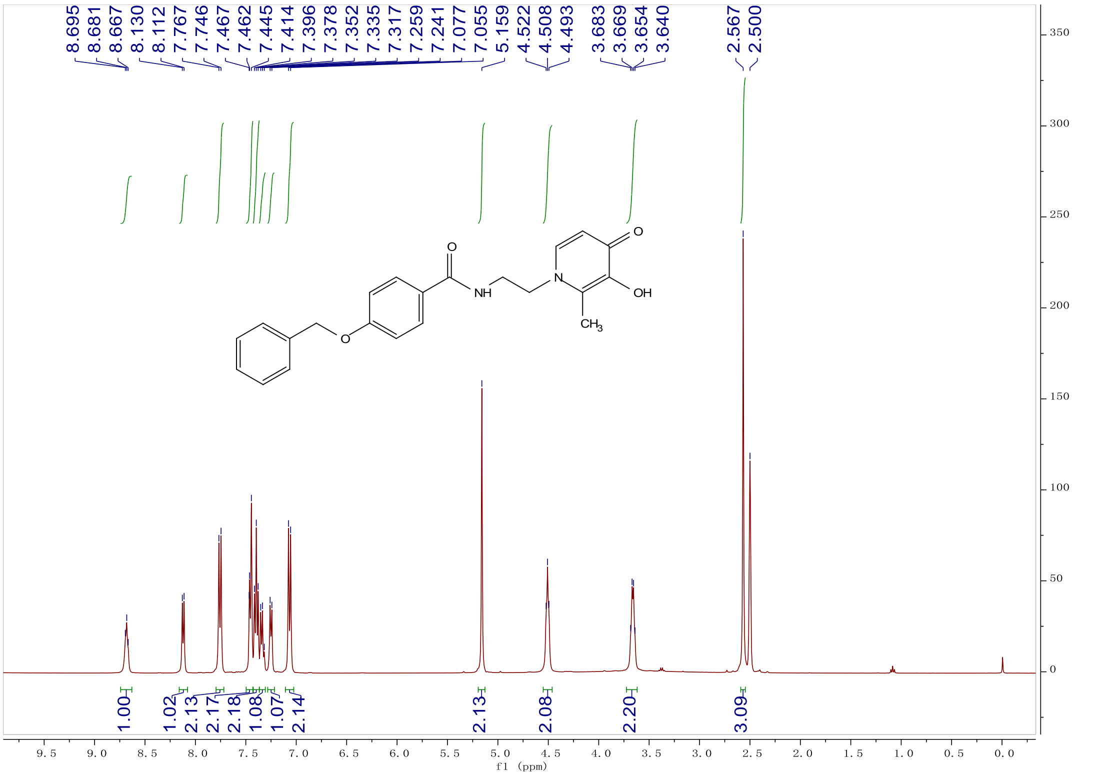

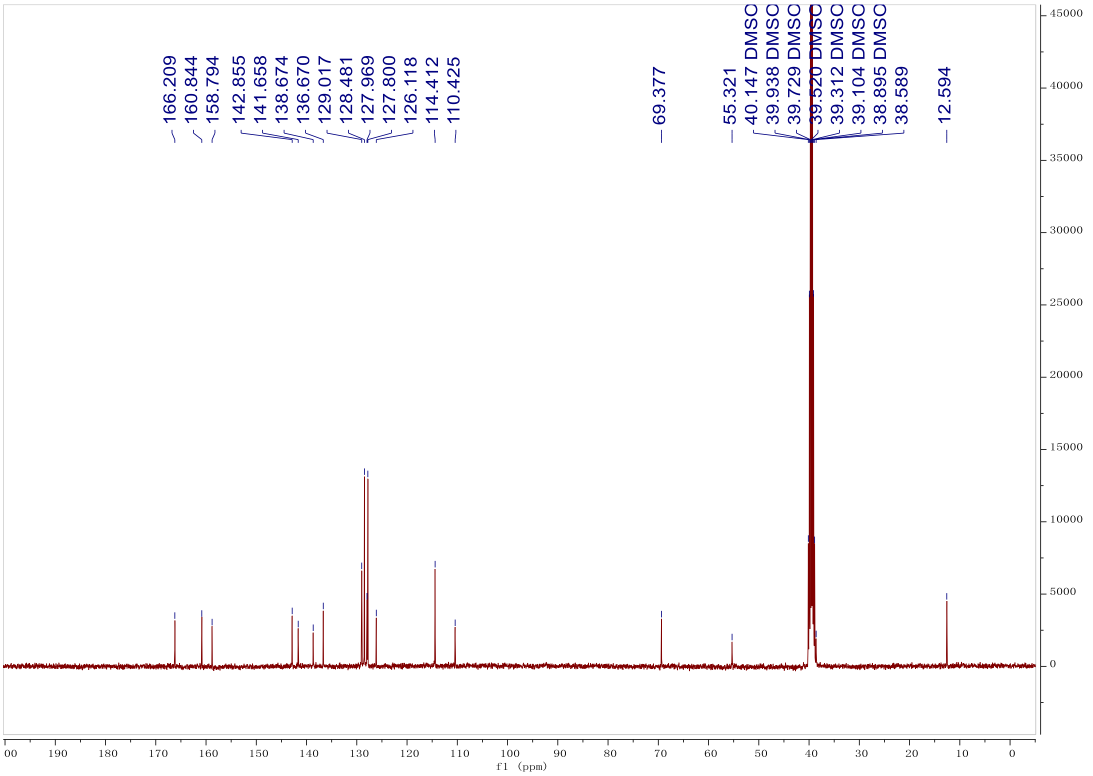

4-Ethoxy-*N*-(2-(3-hydroxy-2-methyl-4-oxopyridin-1(4*H*)-yl)ethyl)benzamide (**8h**)

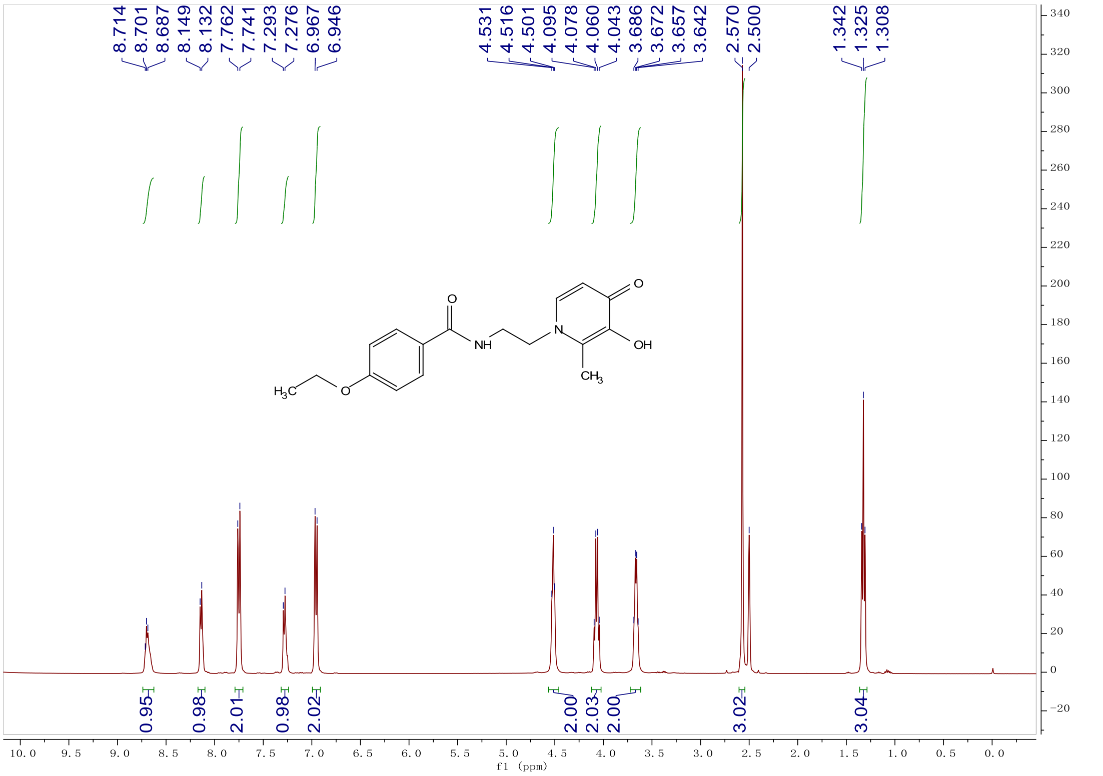

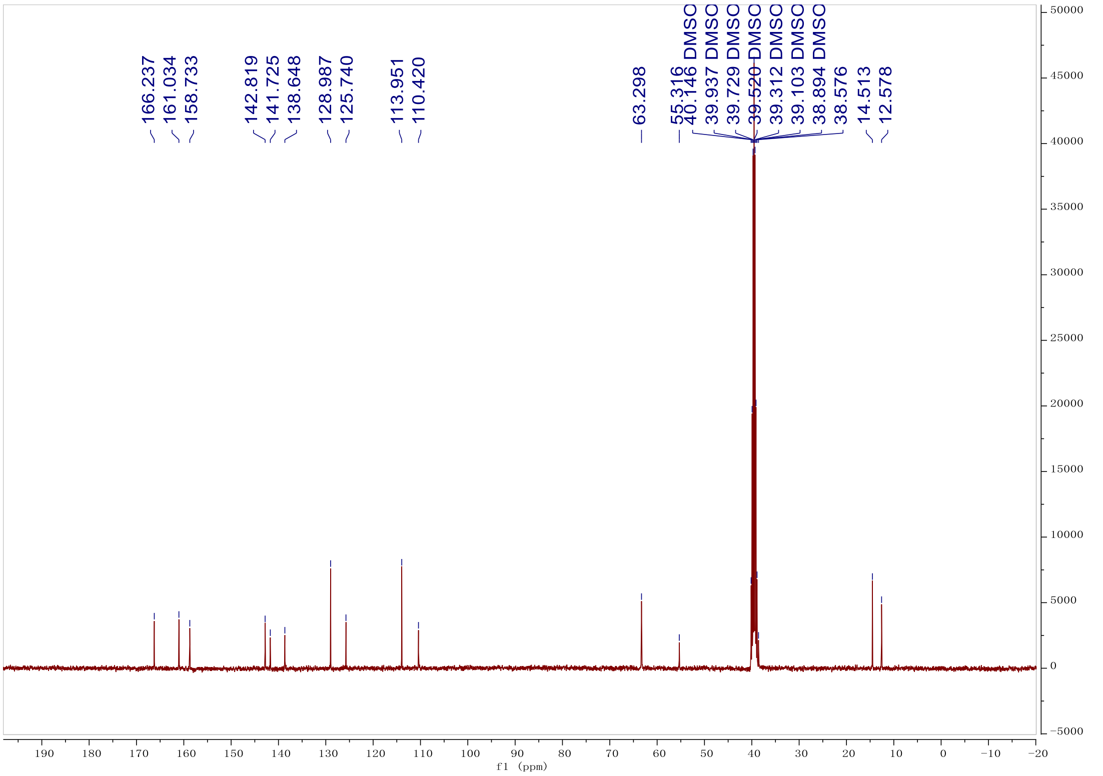

N-(2-(3-hydroxy-2-methyl-4-oxopyridin-1(4*H*)-yl)ethyl)-4-propoxybenzamide (**8i**)

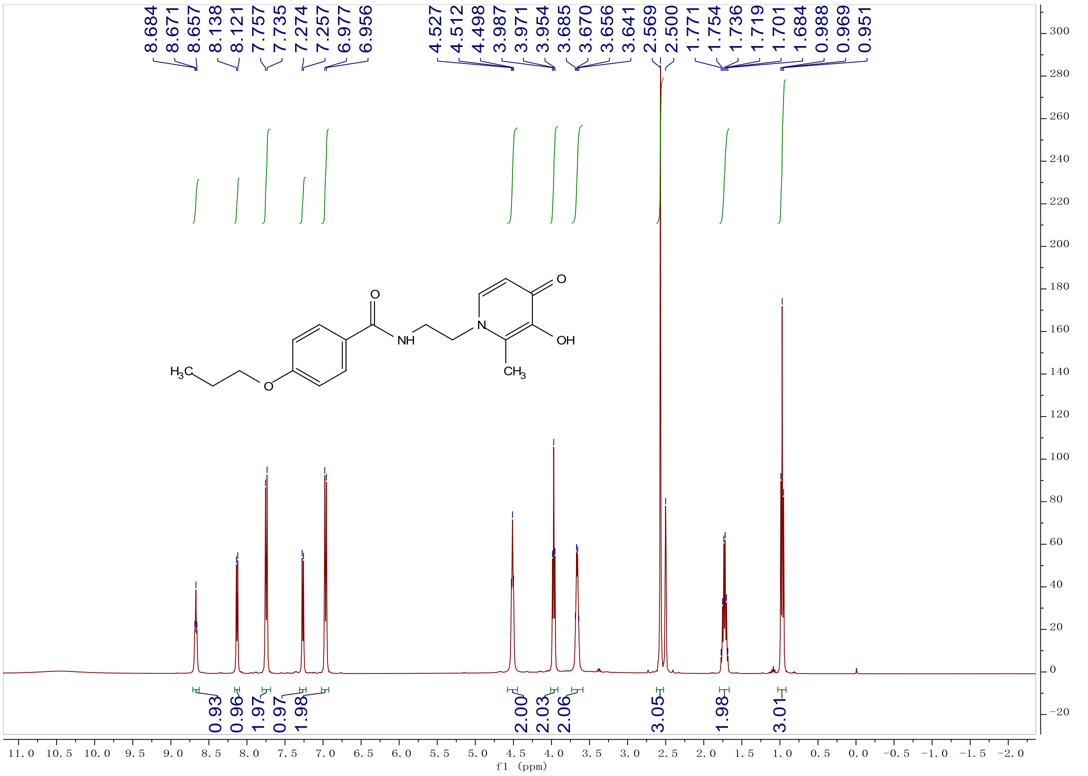

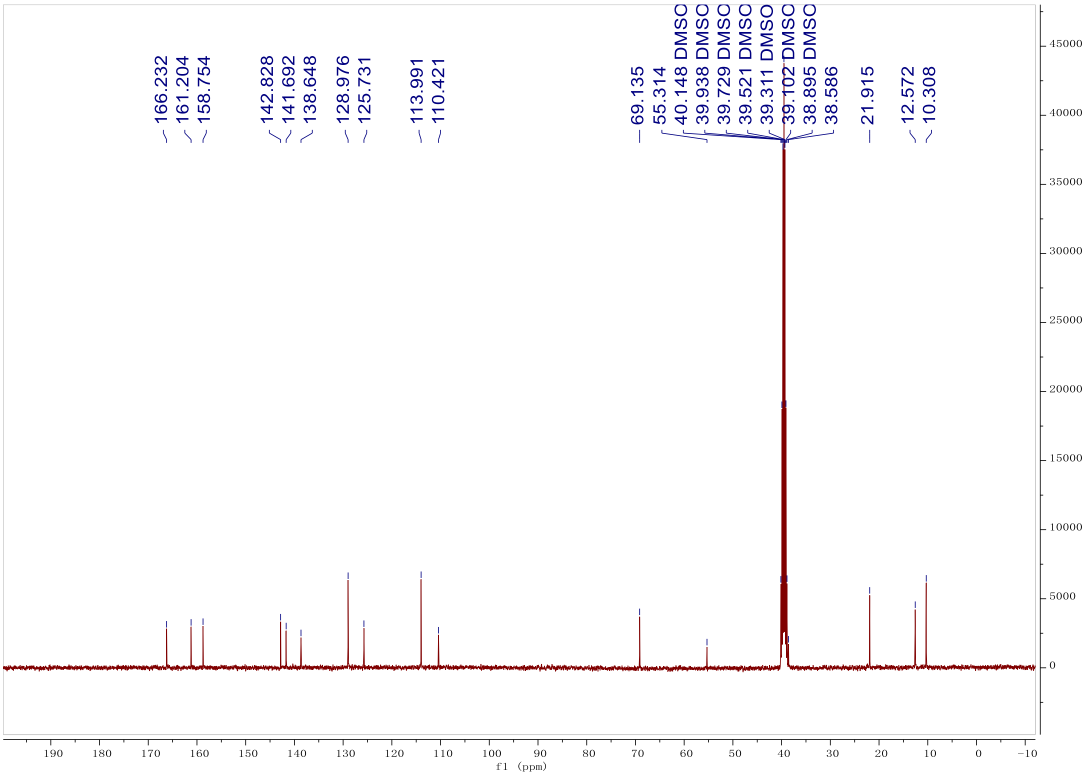

N-(2-(3-hydroxy-2-methyl-4-oxopyridin-1(4*H*)-yl)ethyl)-4-(prop-2-yn- 1-yloxy)benzamide (**8j**)

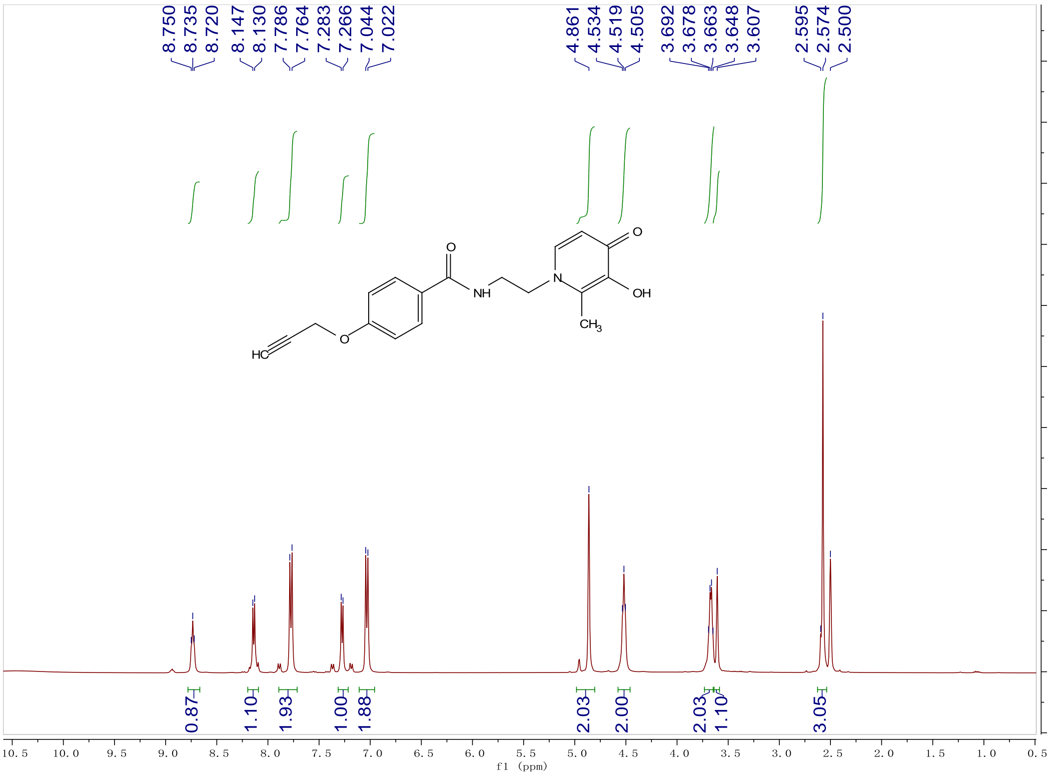

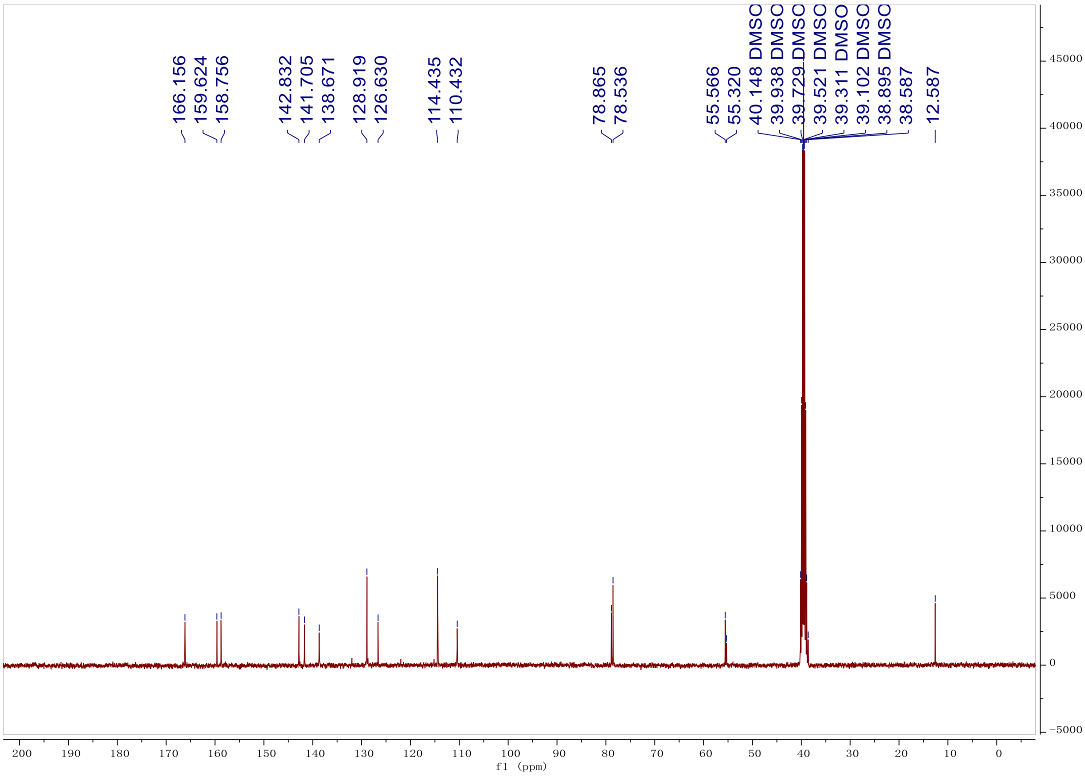

4-(Cyclohexylmethoxy)-*N*-(2-(3-hydroxy-2-methyl-4-oxopyridin-1(4*H*)-yl) ethyl)benzamide (**8k**)

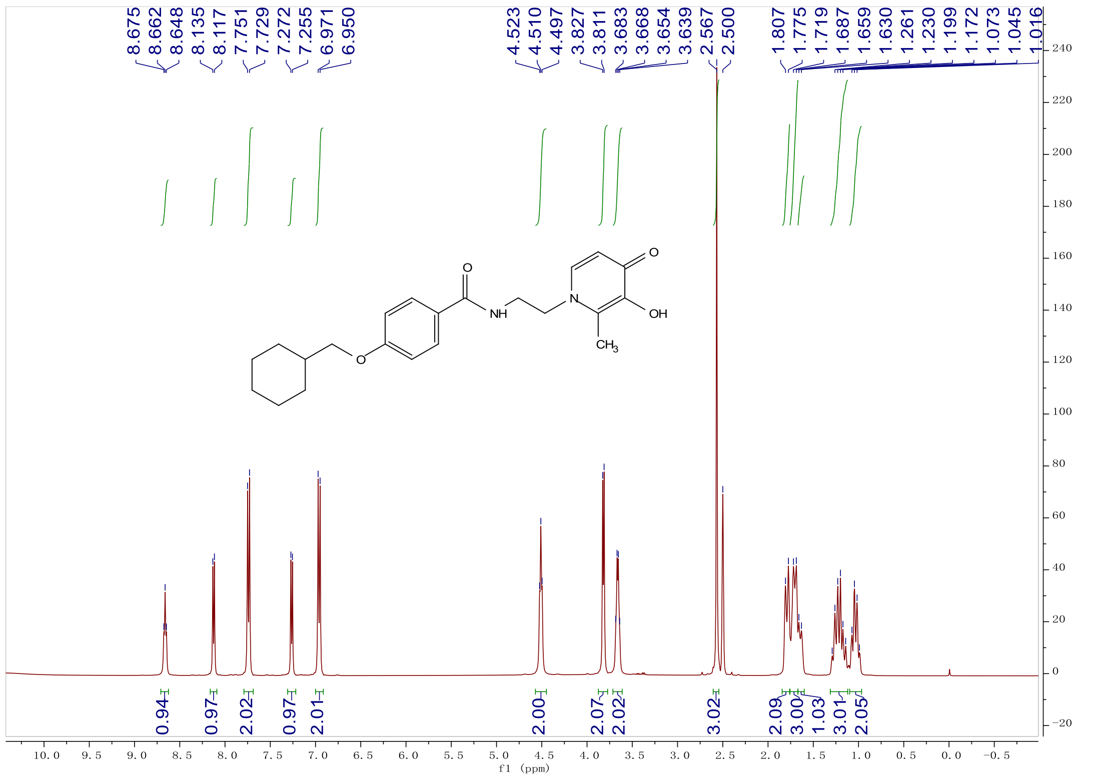

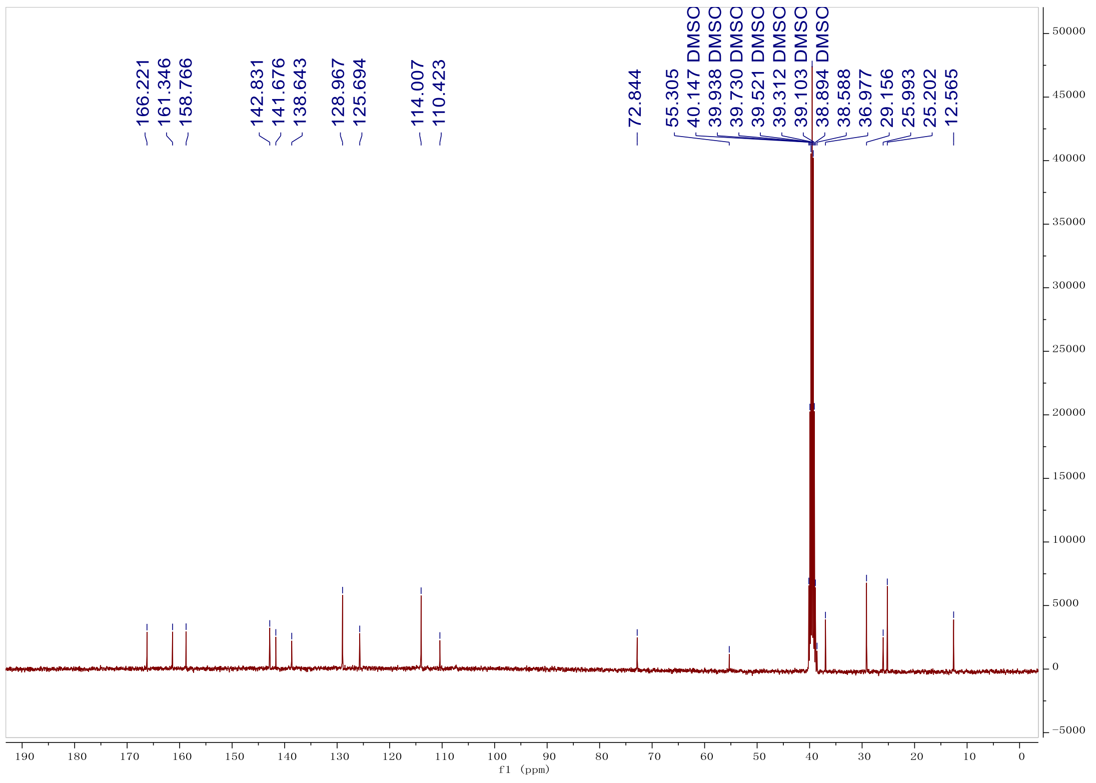

4-((3-Fluorobenzyl)oxy)-*N*-(2-(3-hydroxy-2-methyl-4-oxopyridin-1(4*H*)-yl) ethyl)benzamide (**8l**)

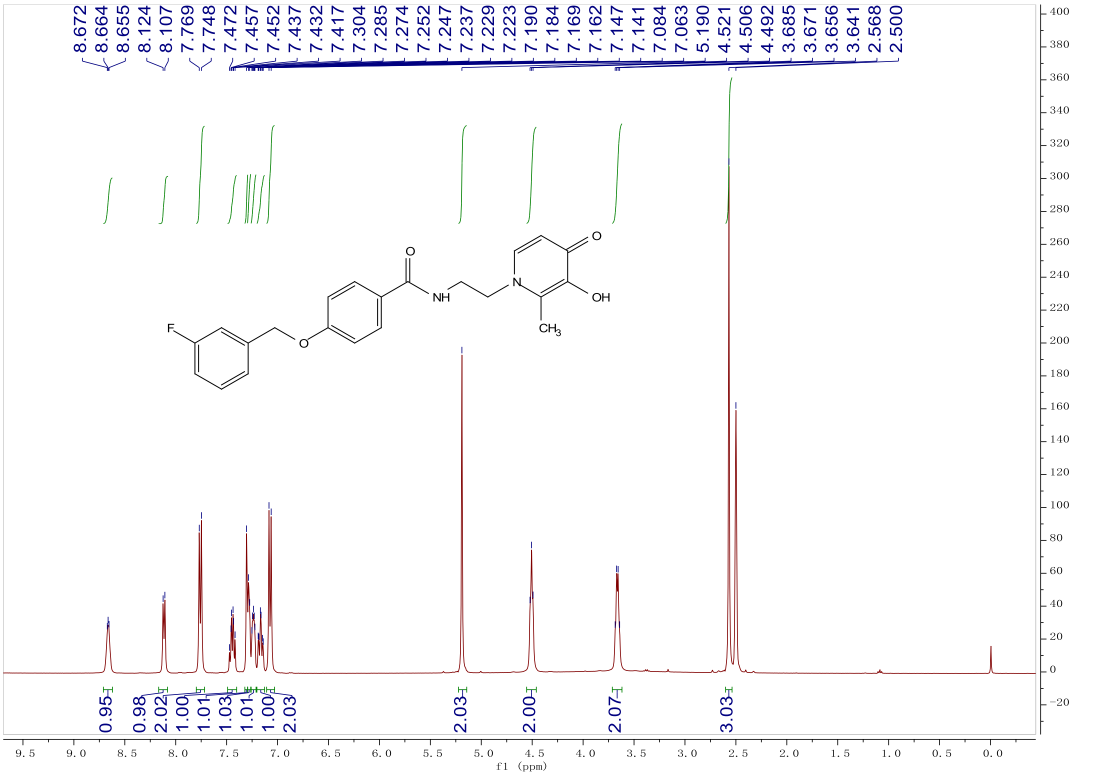

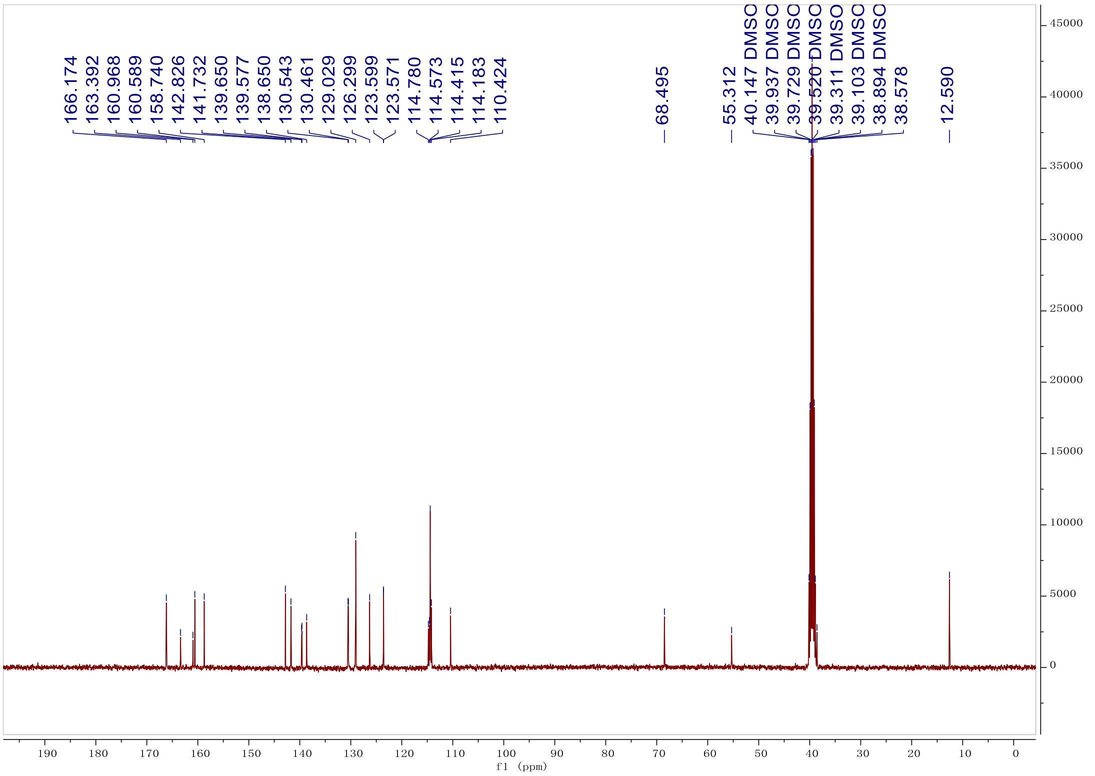

4-((4-Fluorobenzyl)oxy)-*N*-(2-(3-hydroxy-2-methyl-4-oxopyridin-1(4*H*)-yl) ethyl)benzamide (**8m**)

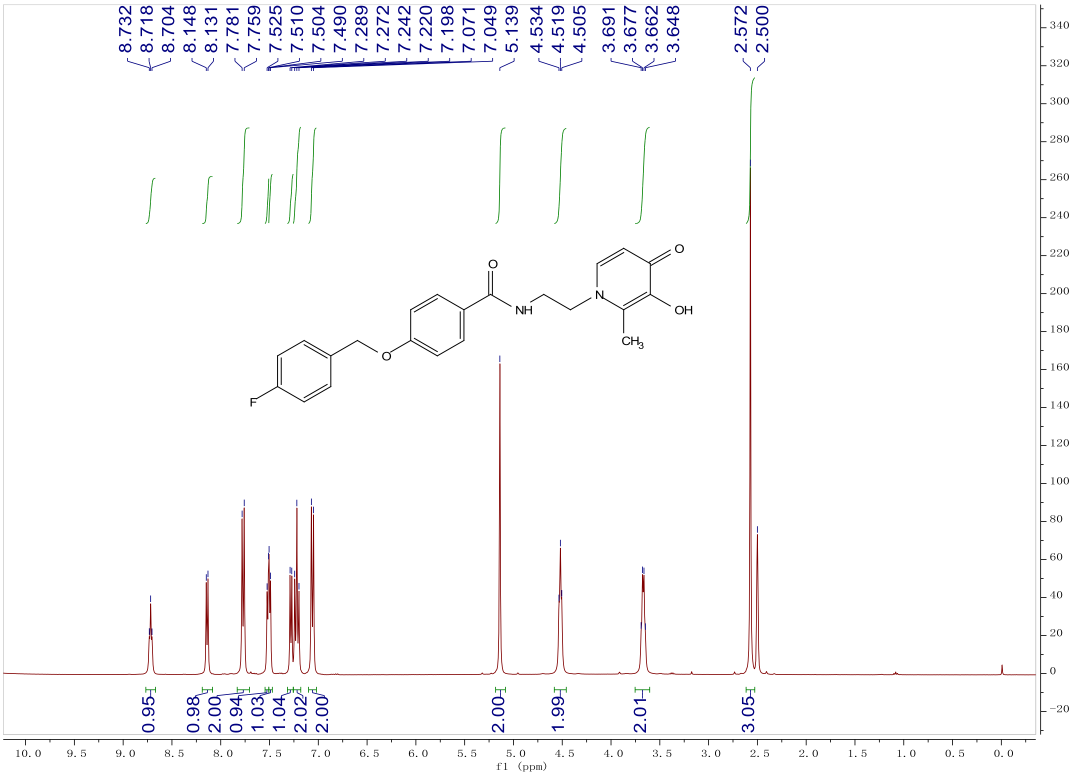

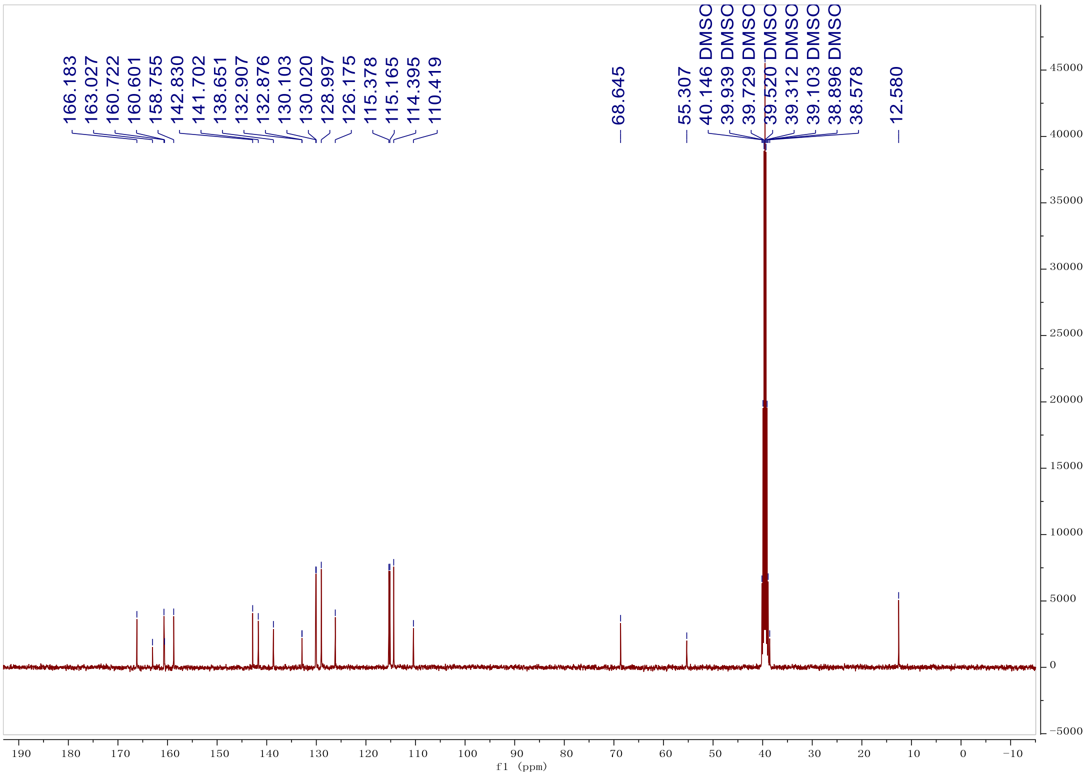

4-((3-Chlorobenzyl)oxy)-*N*-(2-(3-hydroxy-2-methyl-4-oxopyridin-1(4*H*)-yl) ethyl)benzamide (**8n**)

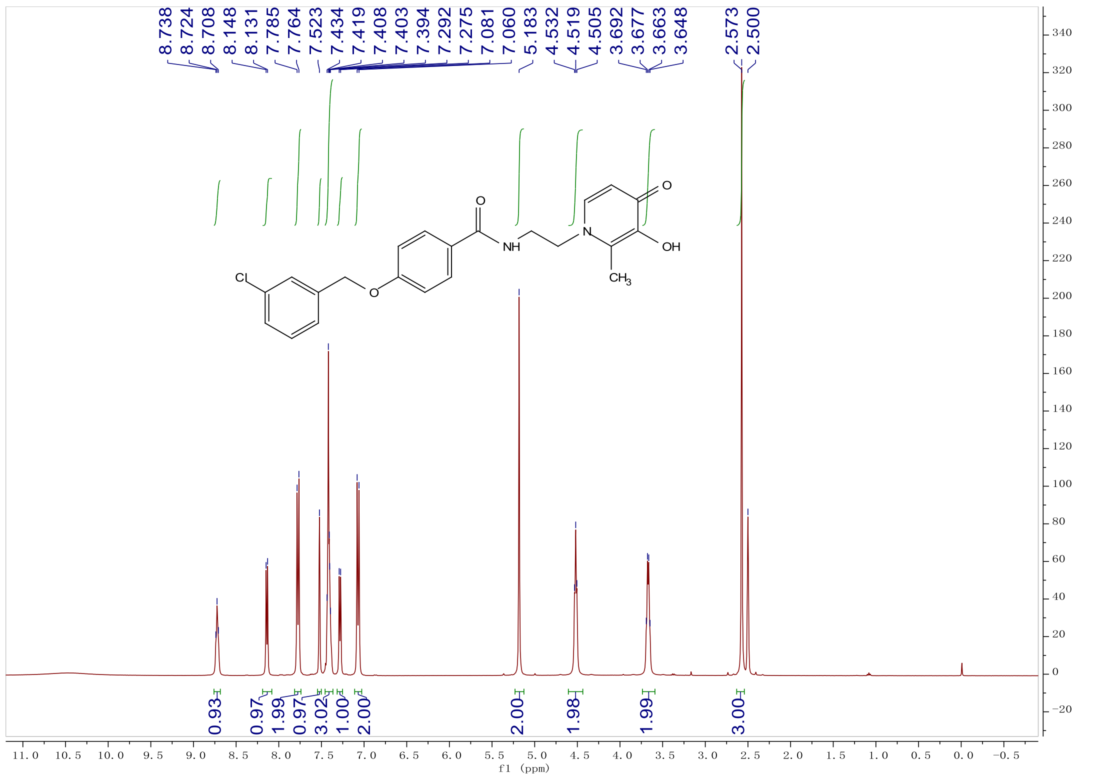

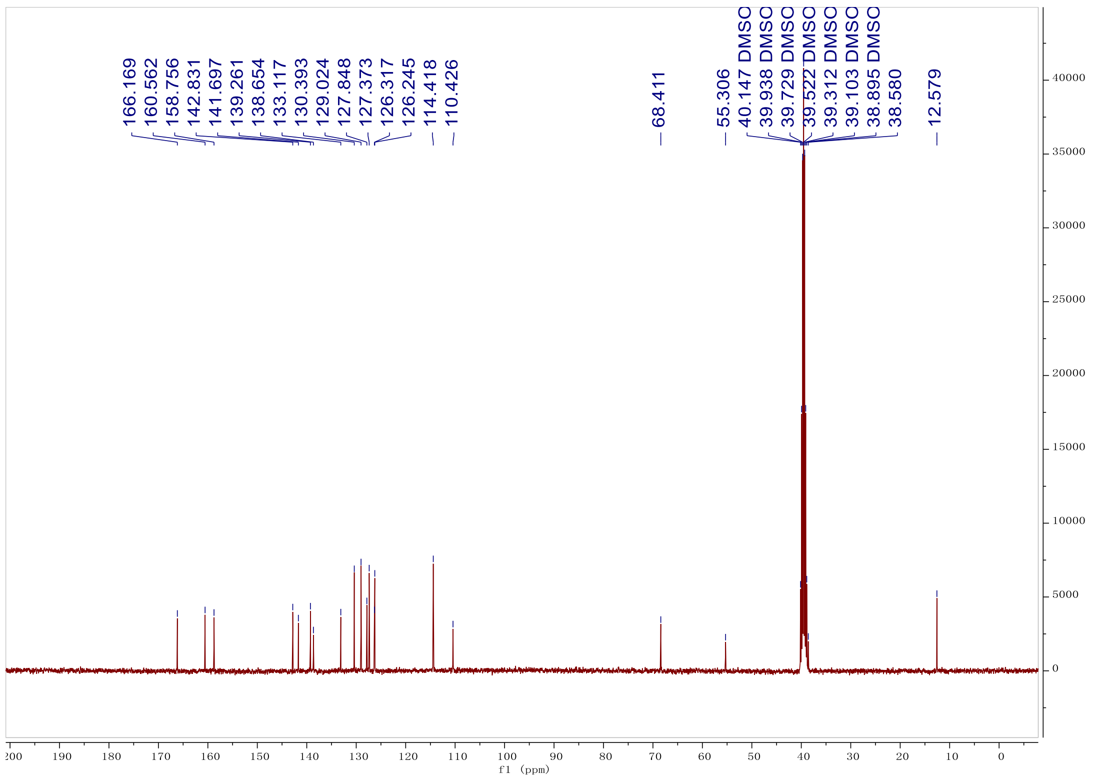

4-((4-Chlorobenzyl)oxy)-*N*-(2-(3-hydroxy-2-methyl-4-oxopyridin-1(4*H*)-yl) ethyl)benzamide (**8o**)

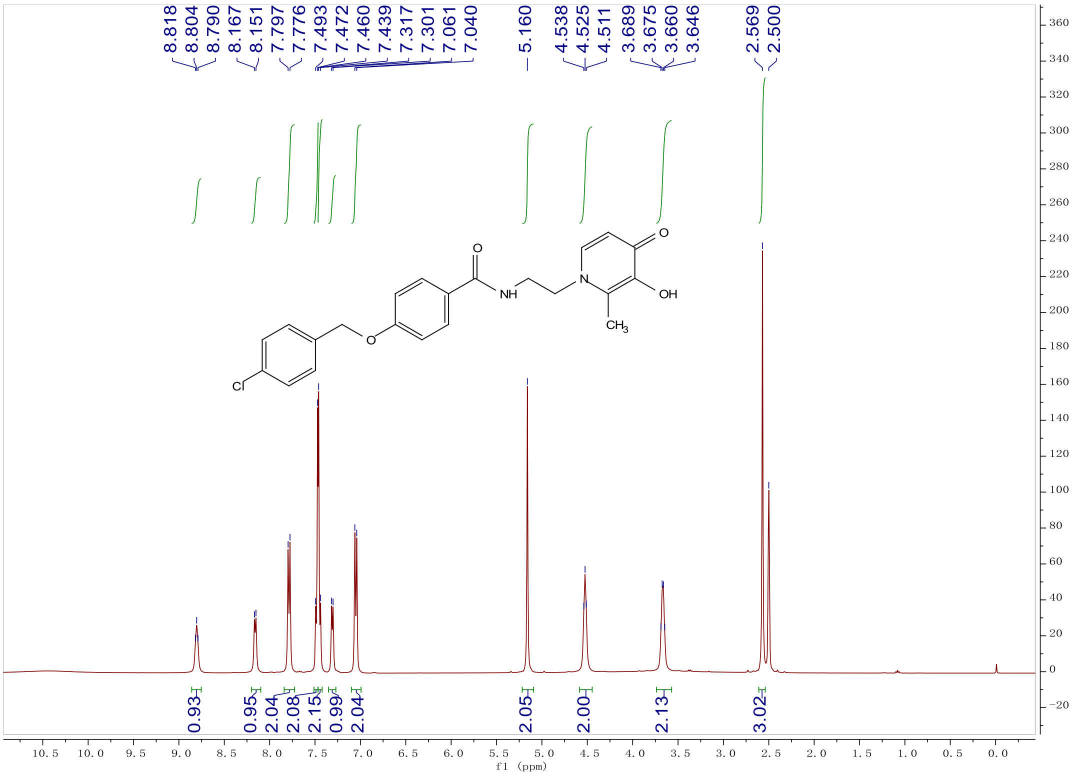

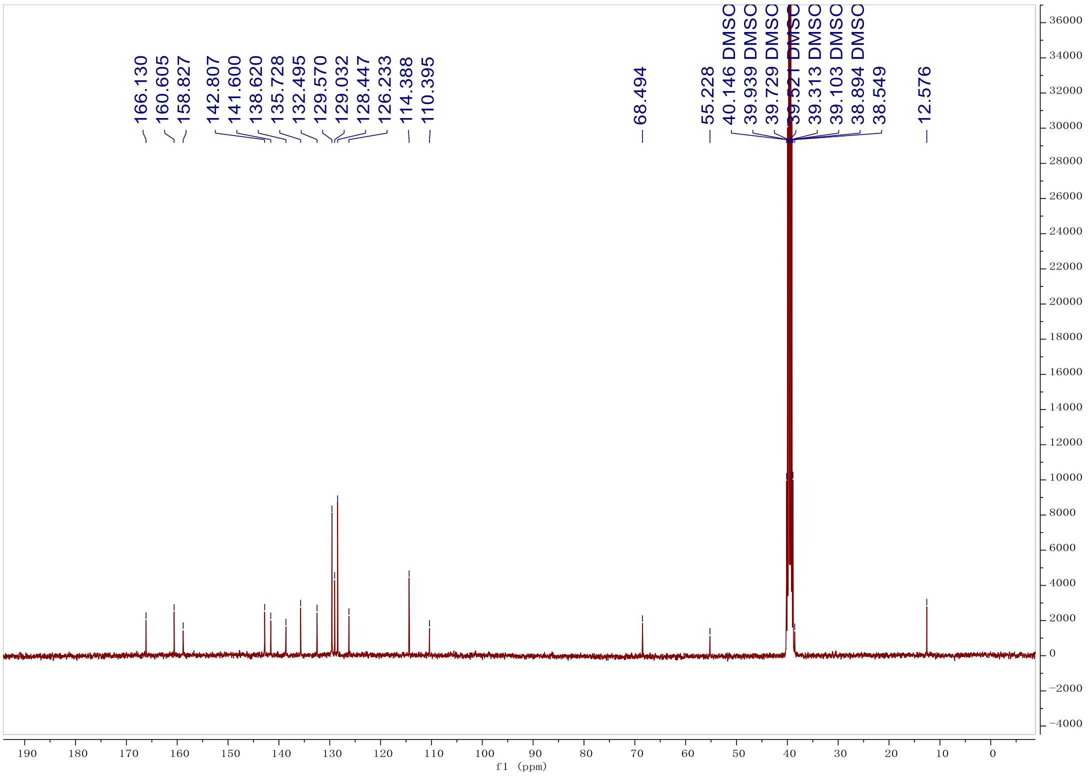

4-((2,5-Difluorobenzyl)oxy)-*N*-(2-(3-hydroxy-2-methyl-4-oxopyridin-1(4*H*) -yl)ethyl)benzamide (**8p**)

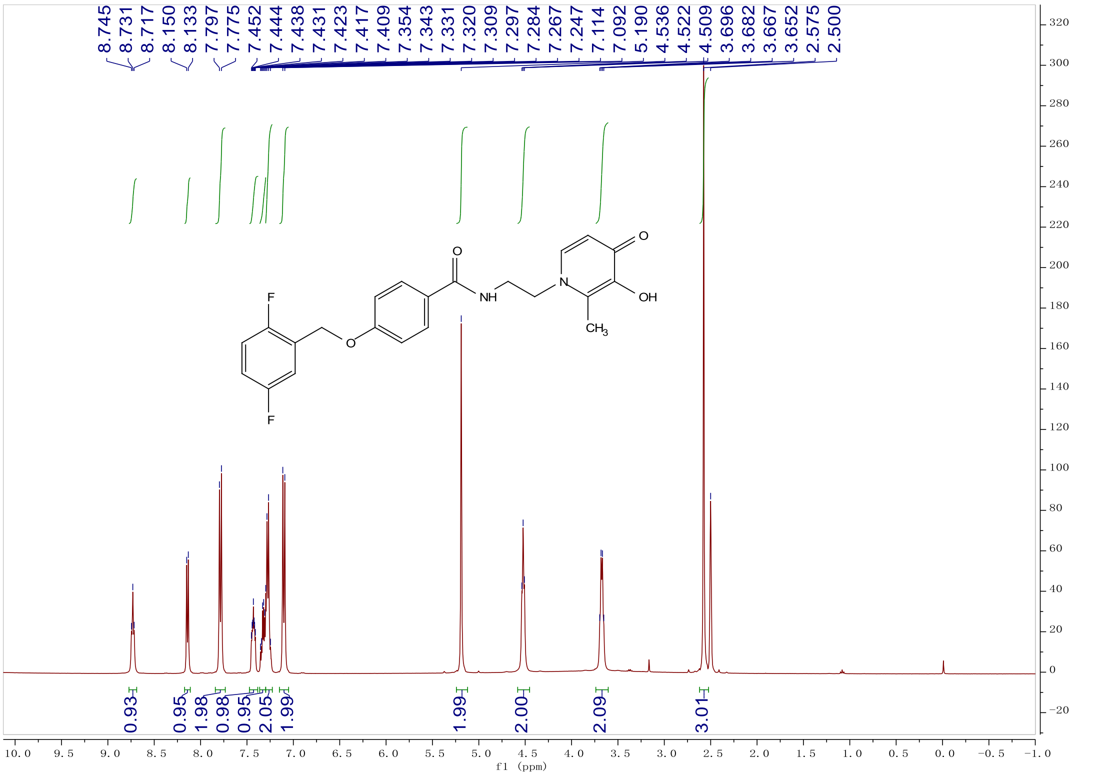

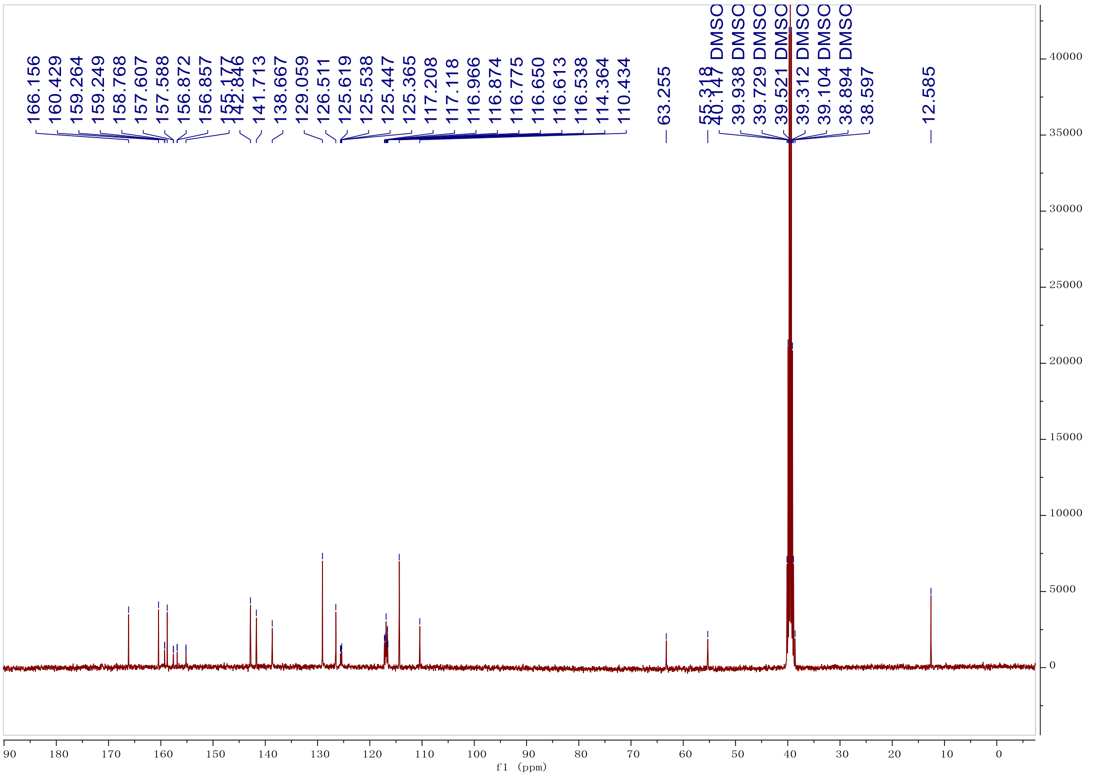

4-((3,5-Difluorobenzyl)oxy)-*N*-(2-(3-hydroxy-2-methyl-4-oxopyridin-1(4*H*) -yl)ethyl)benzamide (**8q**)

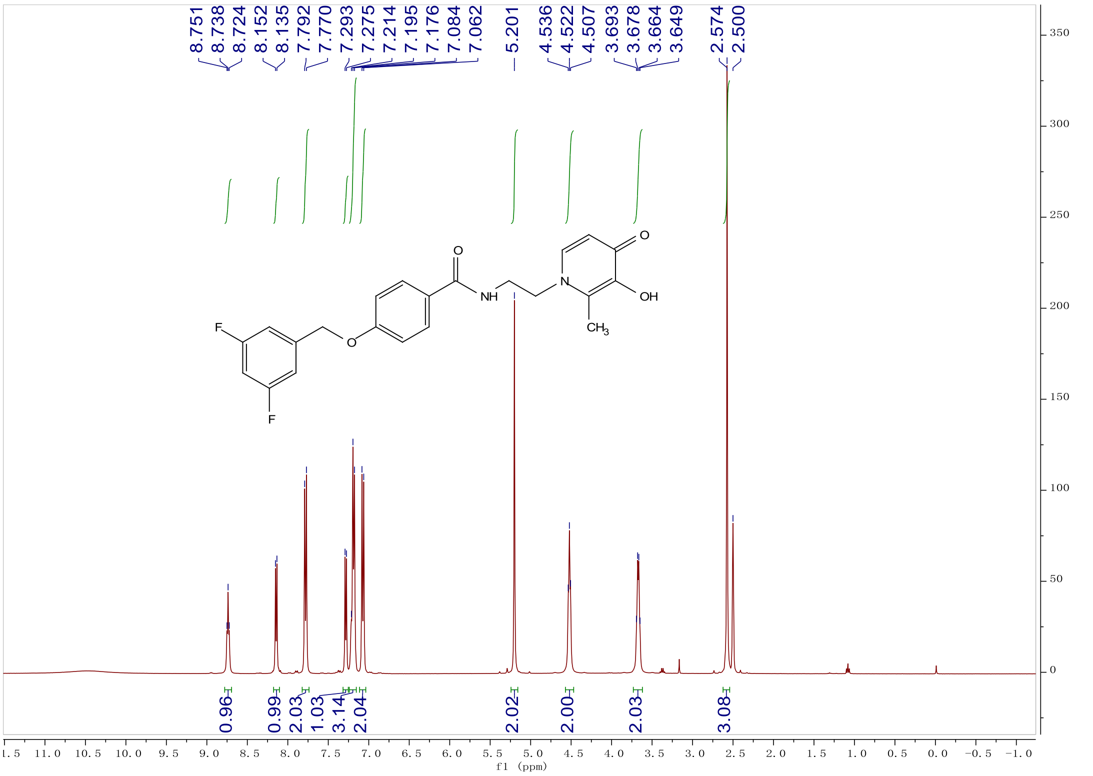

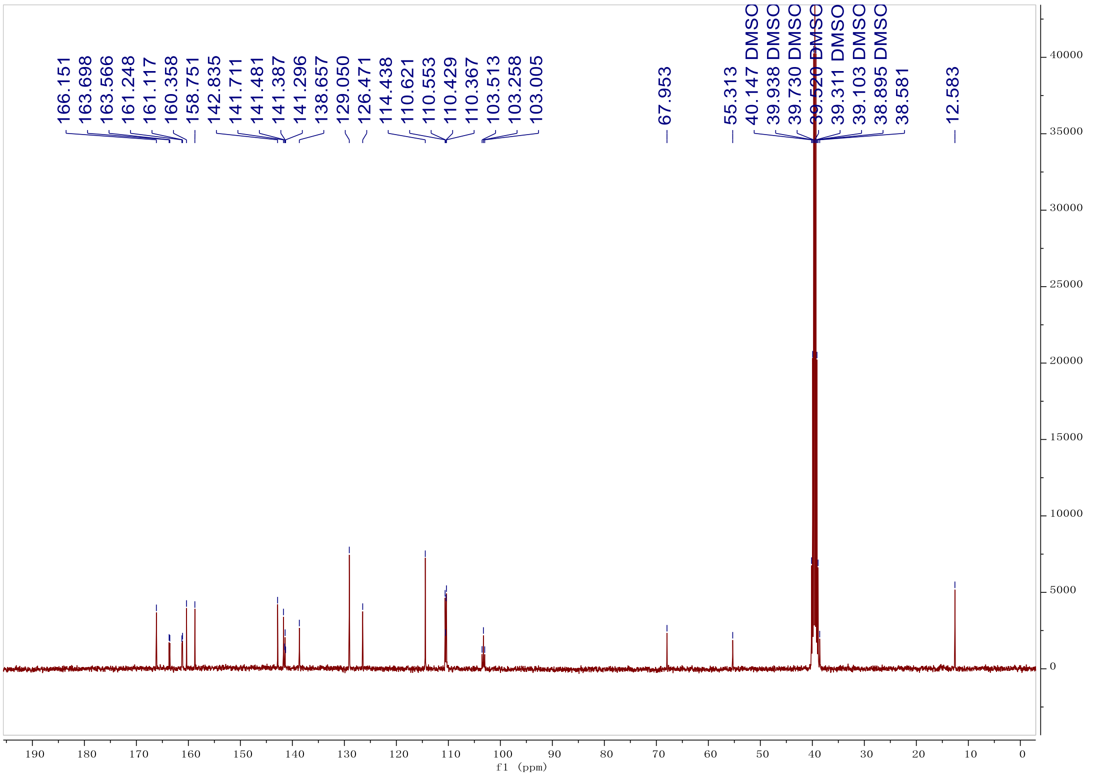

N-(2-(3-hydroxy-2-methyl-4-oxopyridin-1(4*H*)-yl)ethyl)-4-((4-(trifluoromethyl)benzyl)oxy)benzamide (**8r**)

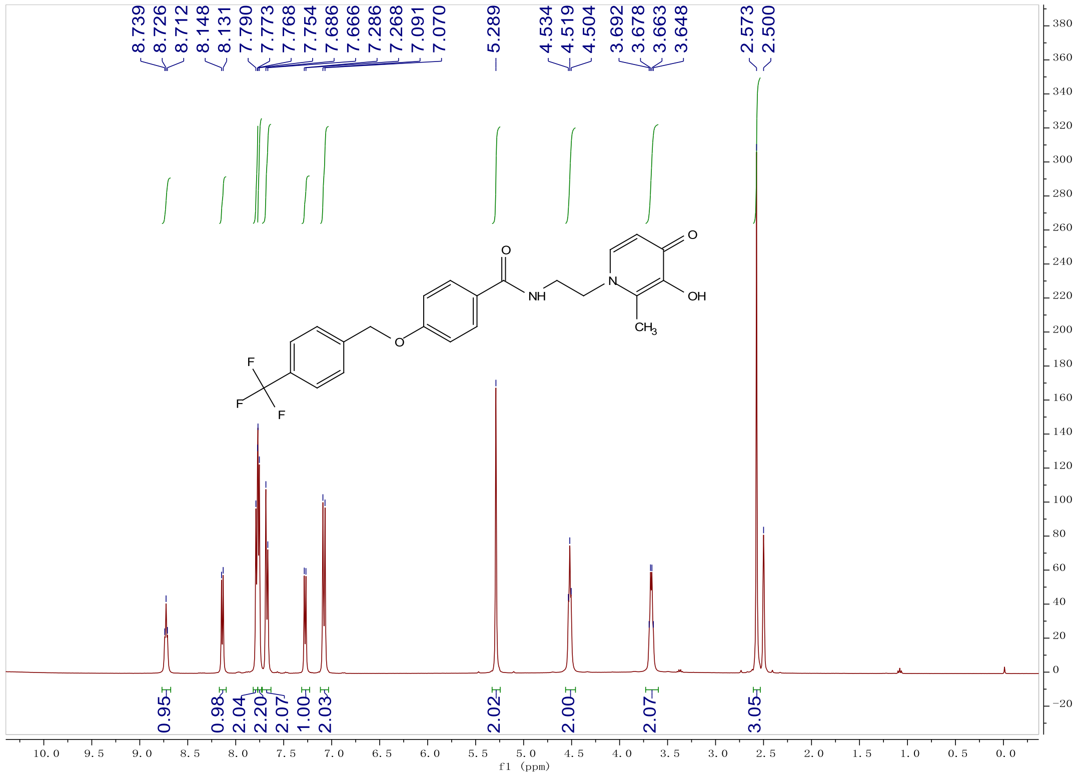

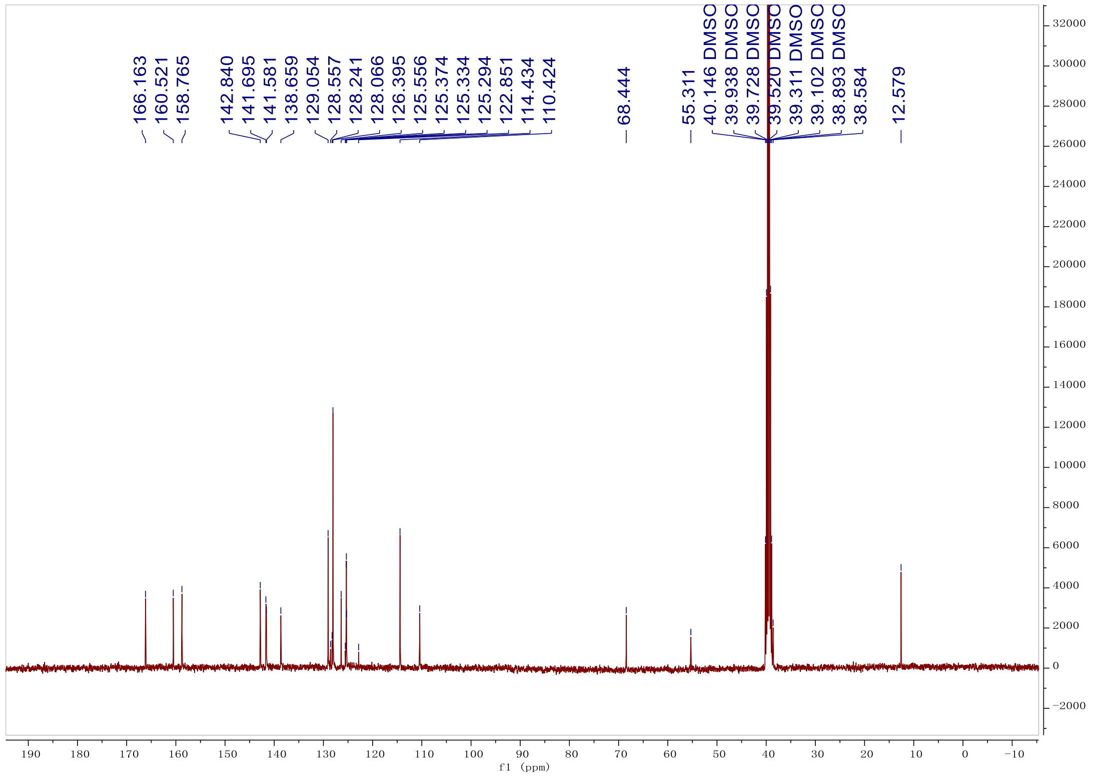

N-(2-(3-hydroxy-2-methyl-4-oxopyridin-1(4*H*)-yl)ethyl)-4-((3-methylbenzyl) oxy)benzamide (**8s**)

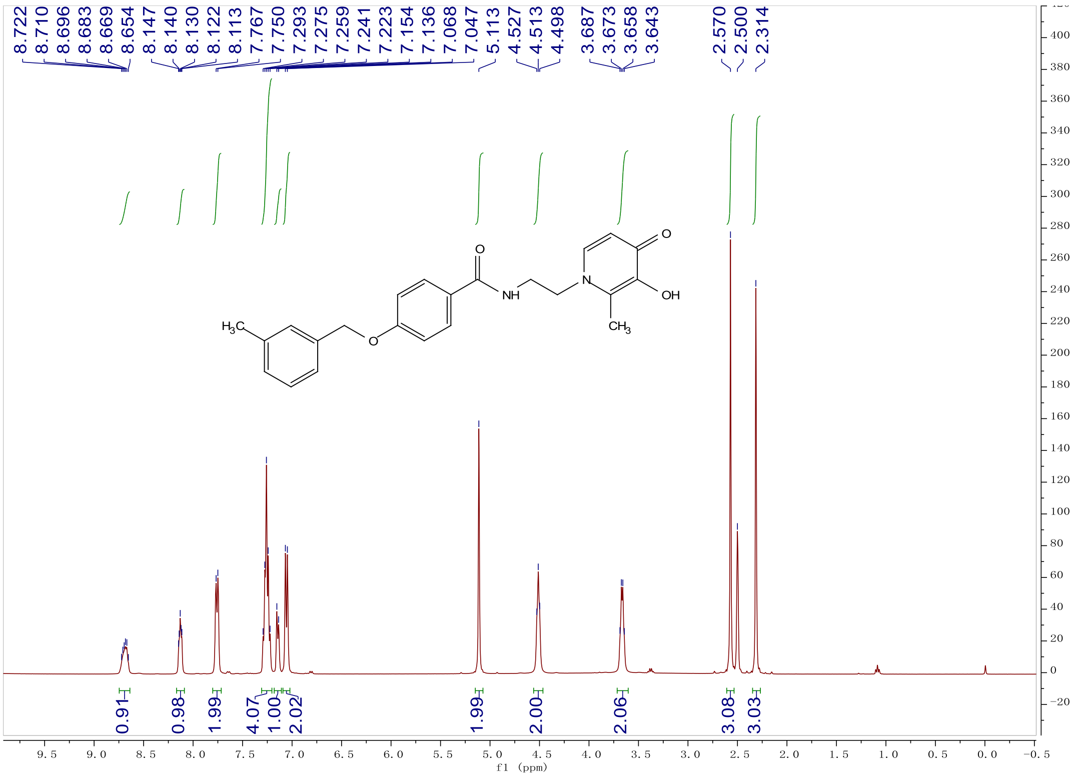

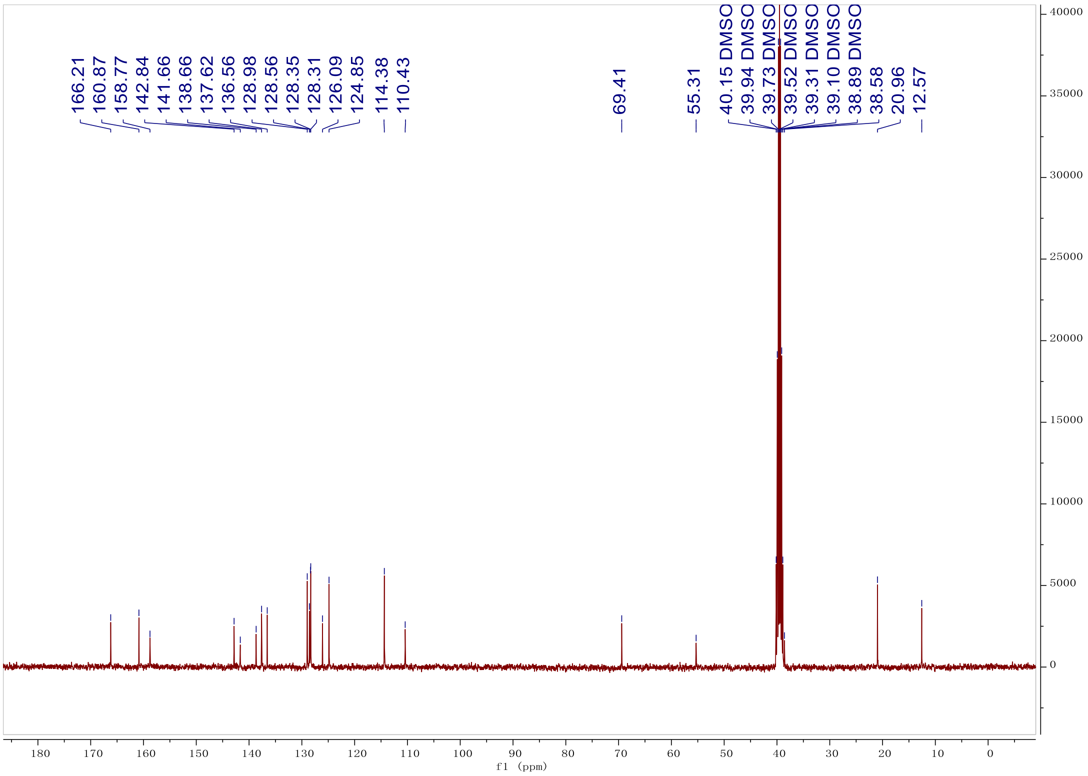

N-(2-(3-hydroxy-2-methyl-4-oxopyridin-1(4*H*)-yl)ethyl)-4-((4-methylbenzyl) oxy)benzamide (**8t**)

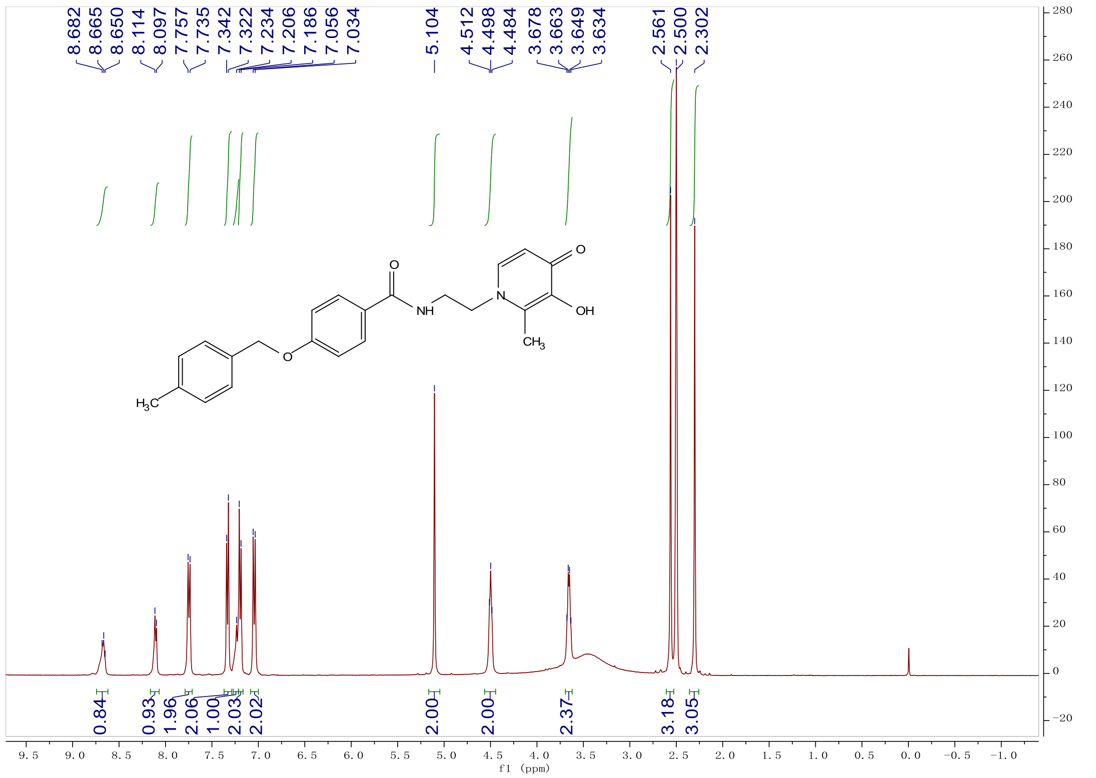

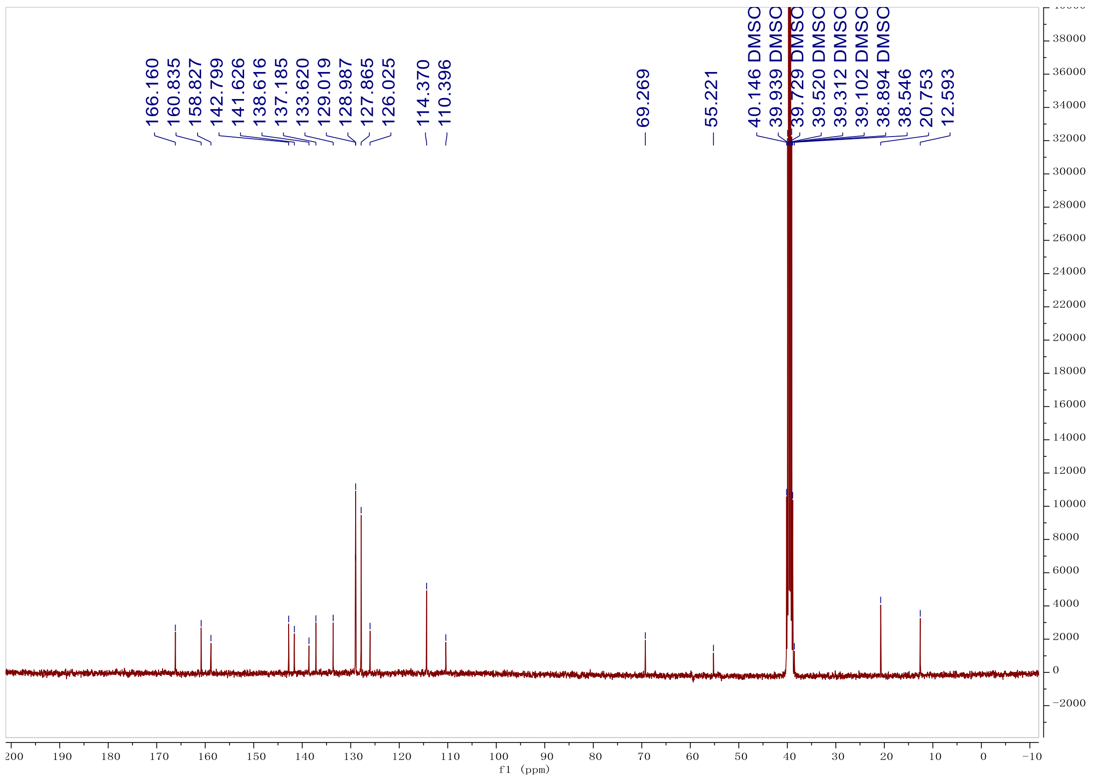

N-(2-(3-hydroxy-2-methyl-4-oxopyridin-1(4*H*)-yl)ethyl)-4-((4-isopropyl benzyl)oxy)benzamide (**8u**)

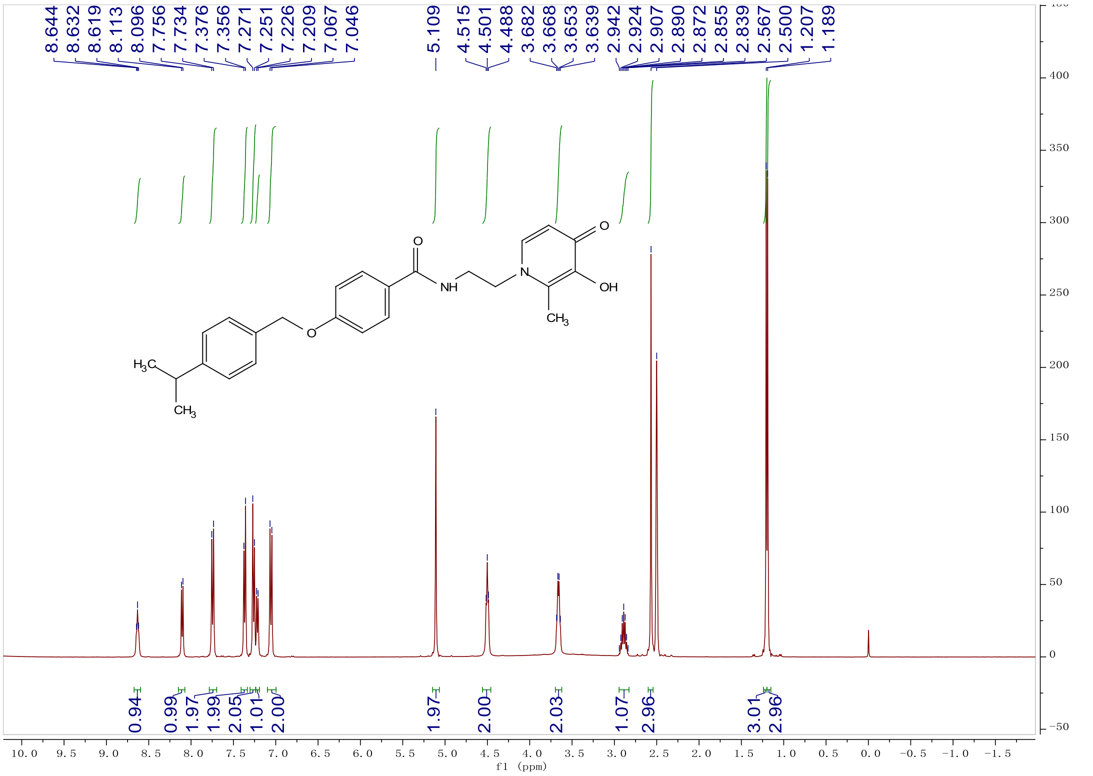

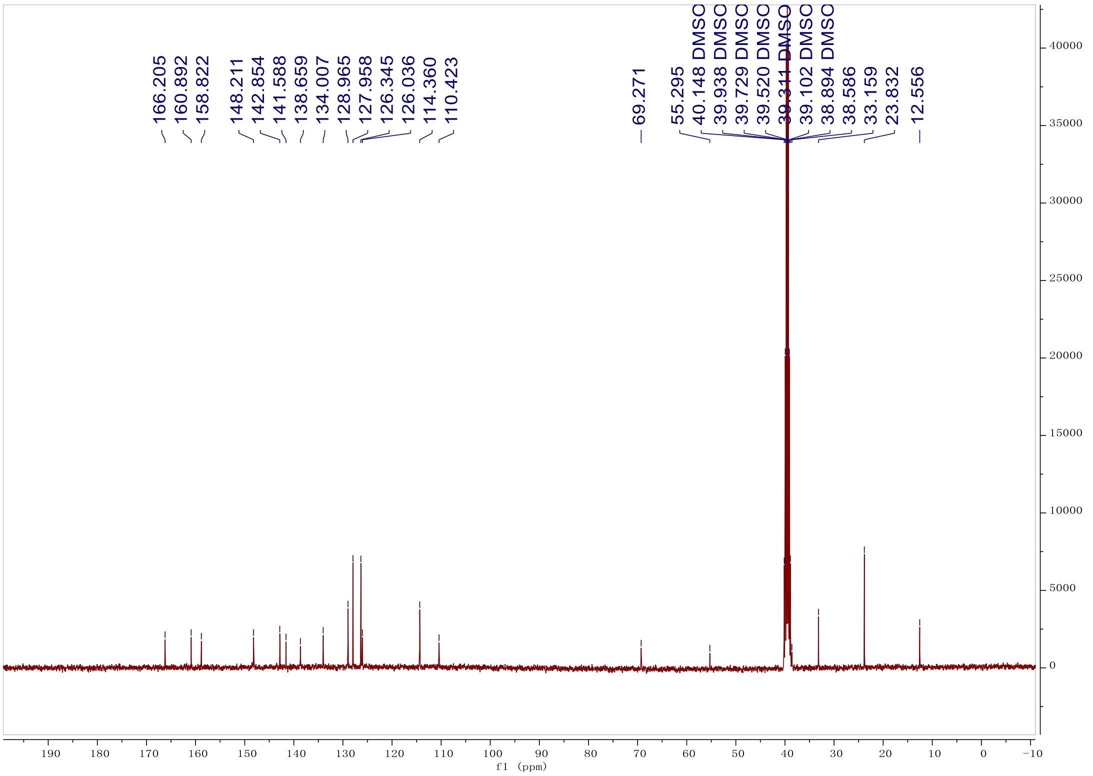

4-((4-(*tert*-Butyl)benzyl)oxy)-*N*-(2-(3-hydroxy-2-methyl-4-oxopyridin-1(4*H*) -yl)ethyl)benzamide (**8v**)

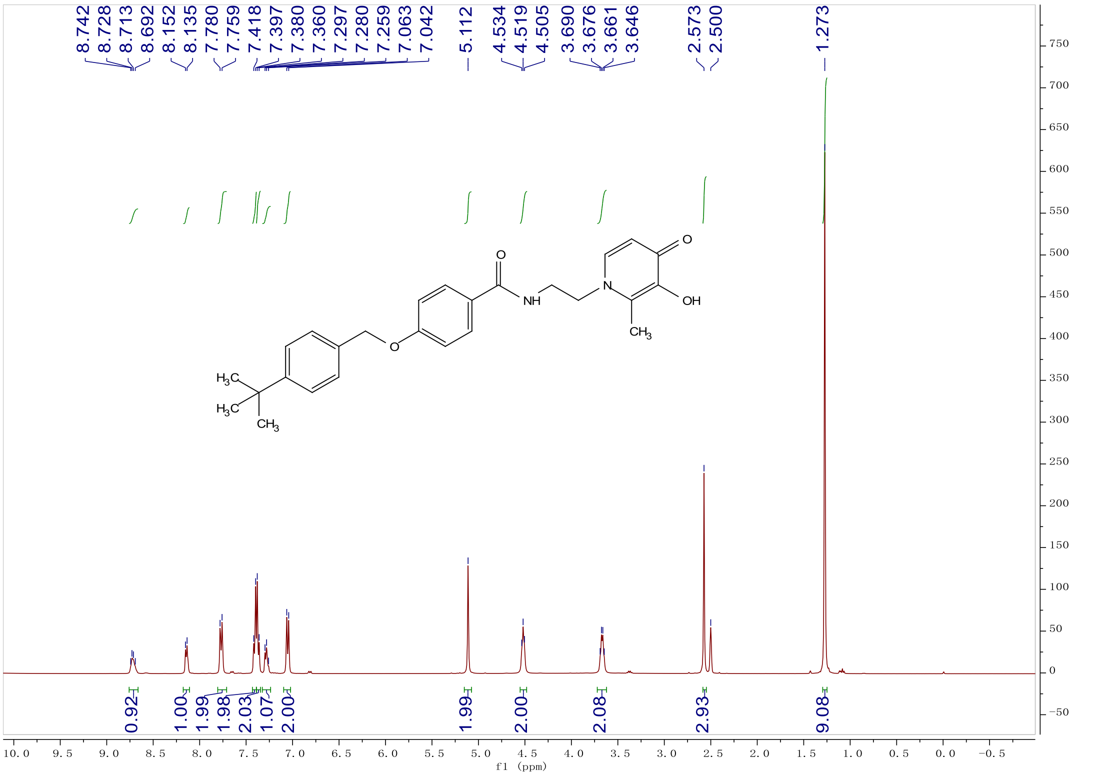

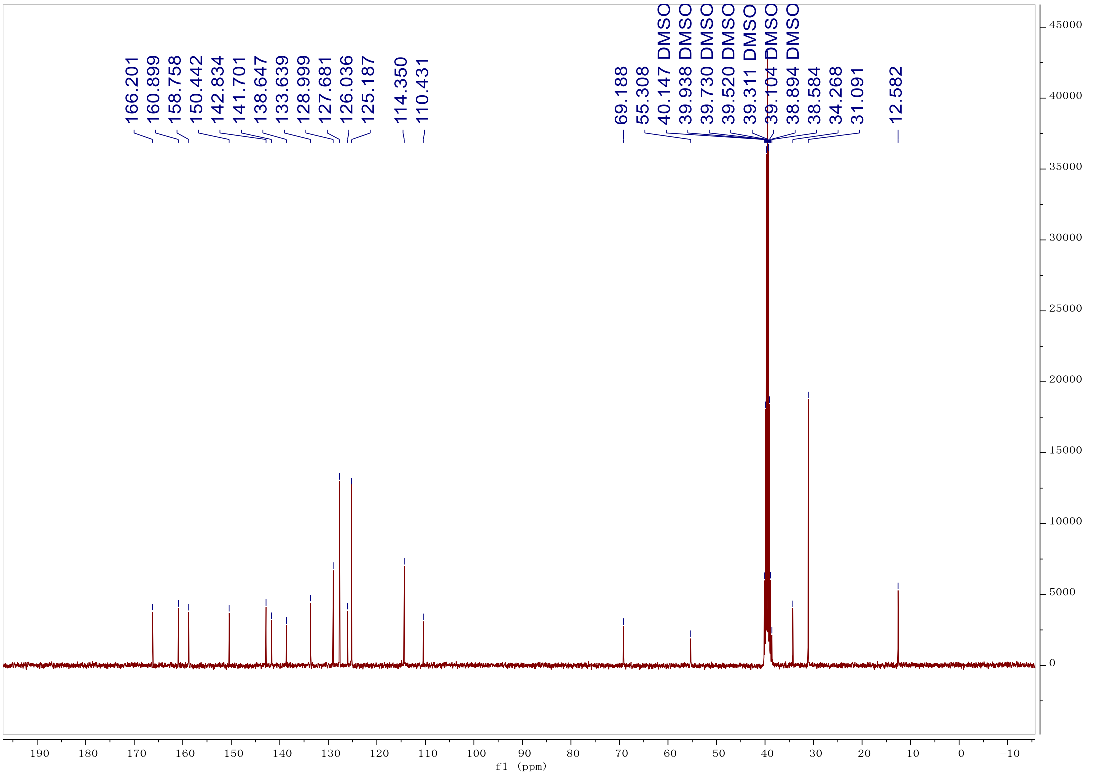

2-Hydroxy-*N*-(2-(3-hydroxy-2-methyl-4-oxopyridin-1(4*H*)-yl)ethyl) benzamide (**8w**)

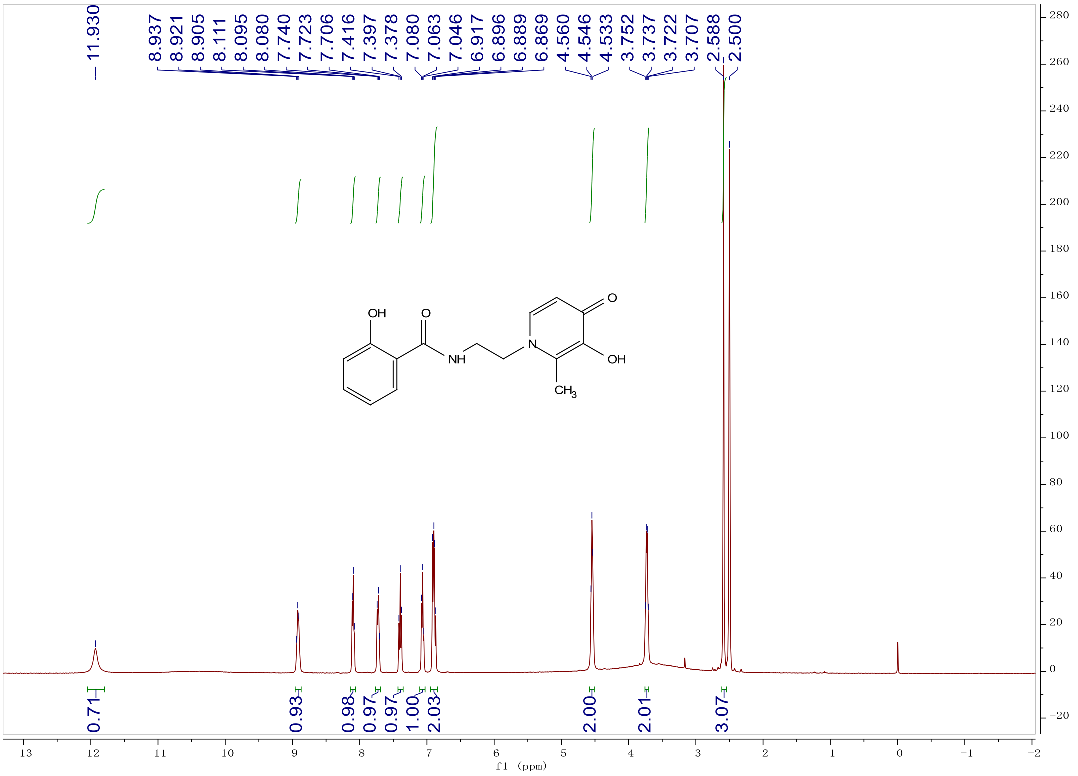

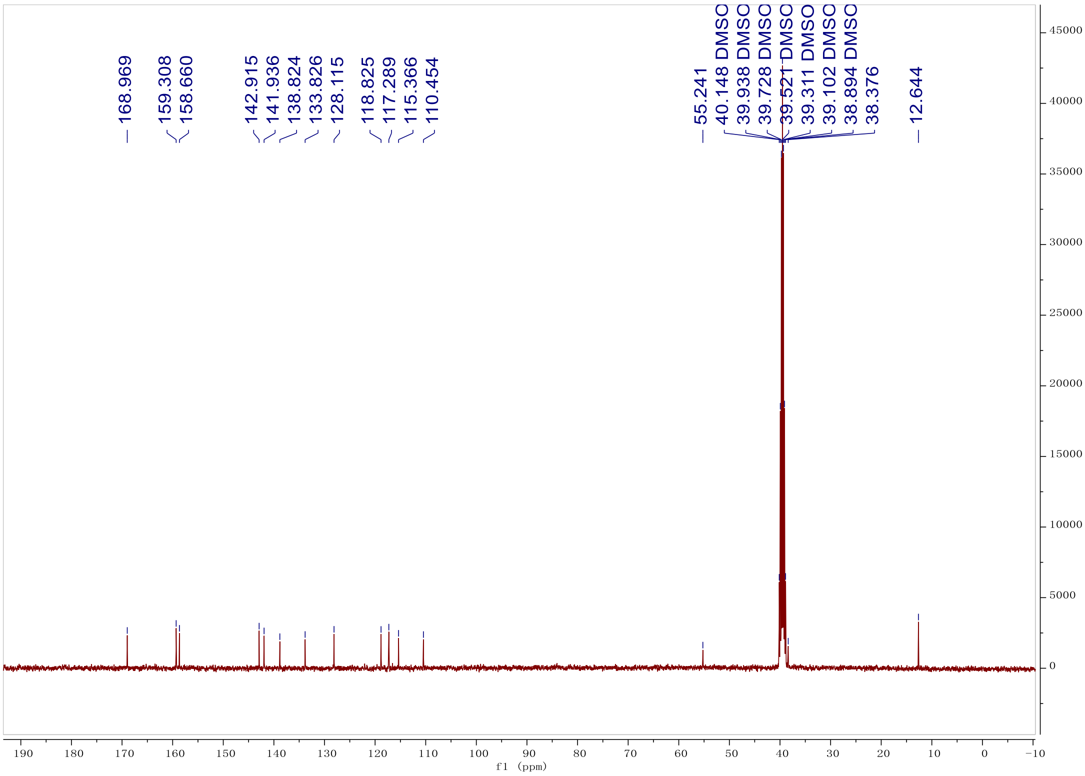

3-Hydroxy-*N*-(2-(3-hydroxy-2-methyl-4-oxopyridin-1(4*H*)-yl)ethyl) benzamide (**8x**)

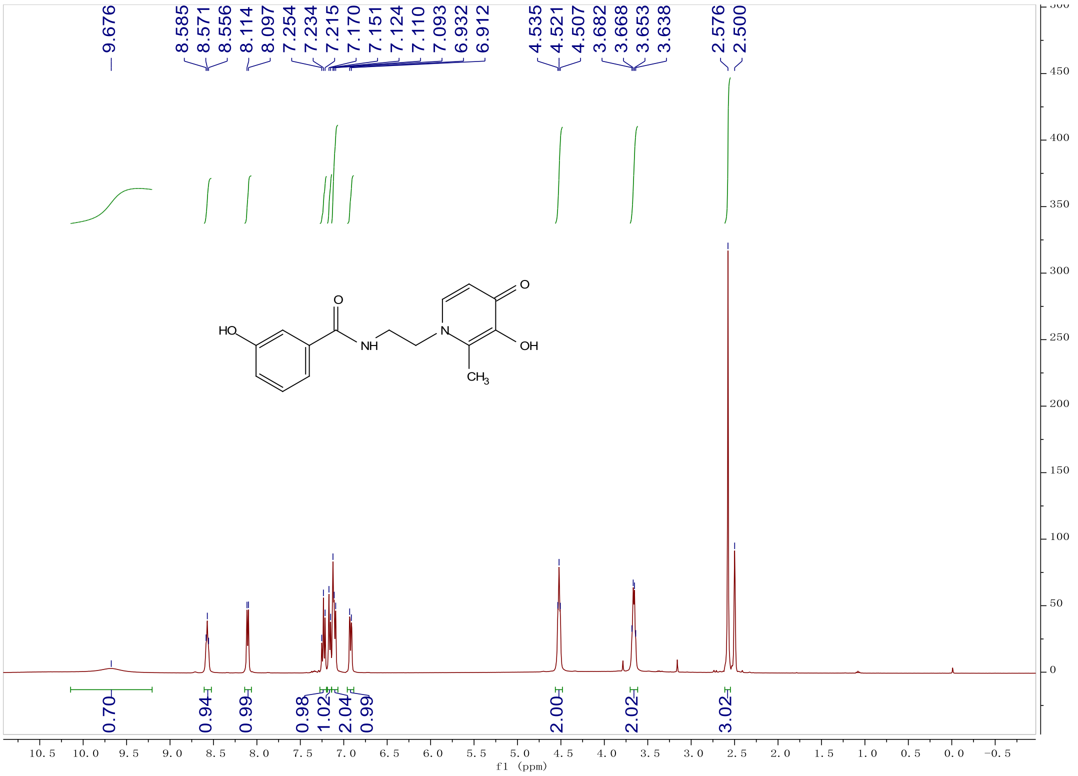

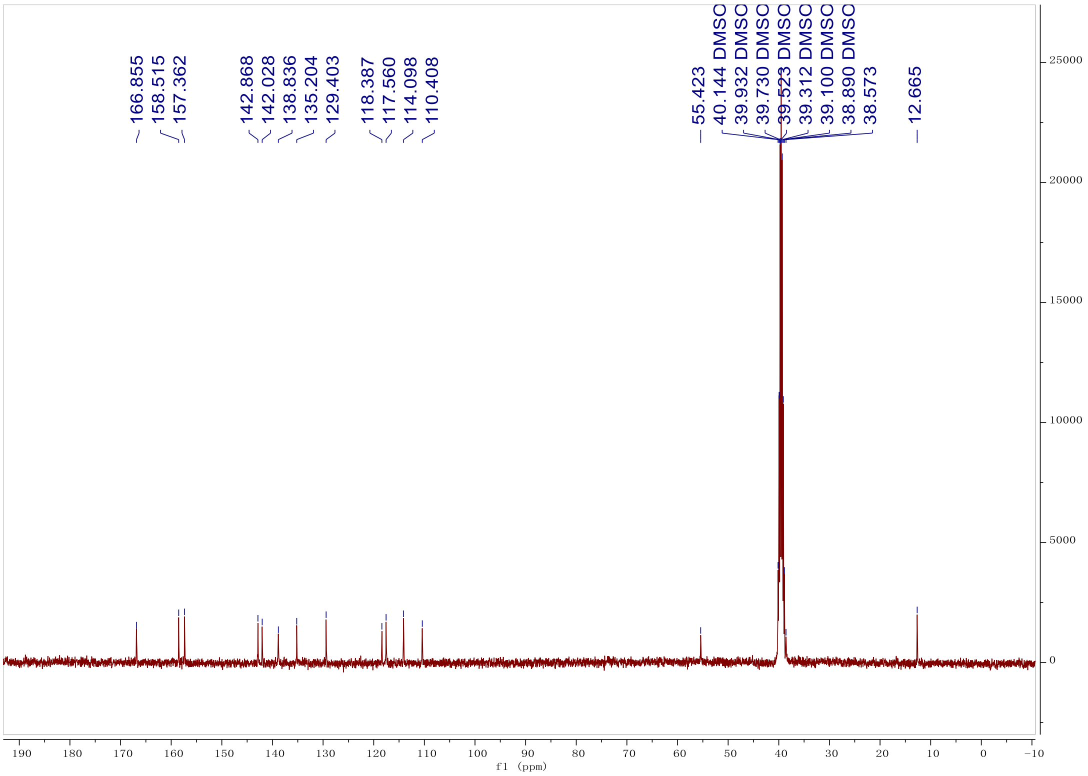

4-Hydroxy-*N*-(2-(3-hydroxy-2-methyl-4-oxopyridin-1(4*H*)-yl)ethyl) benzamide (**8y**)

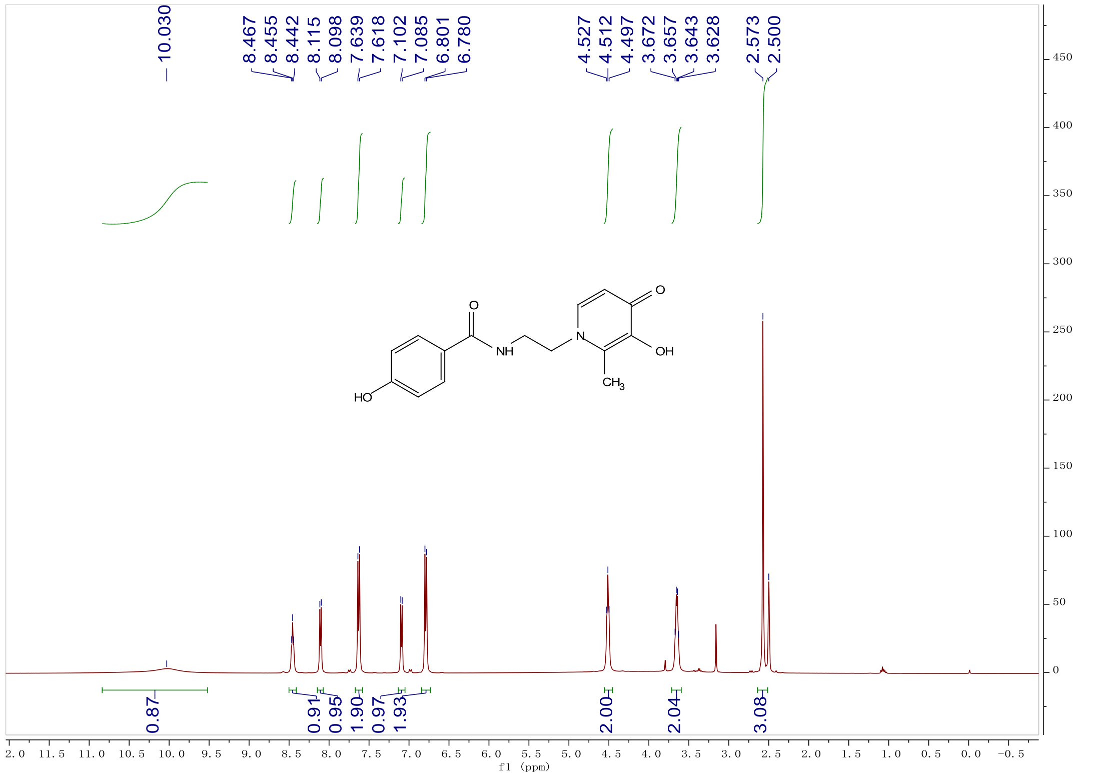

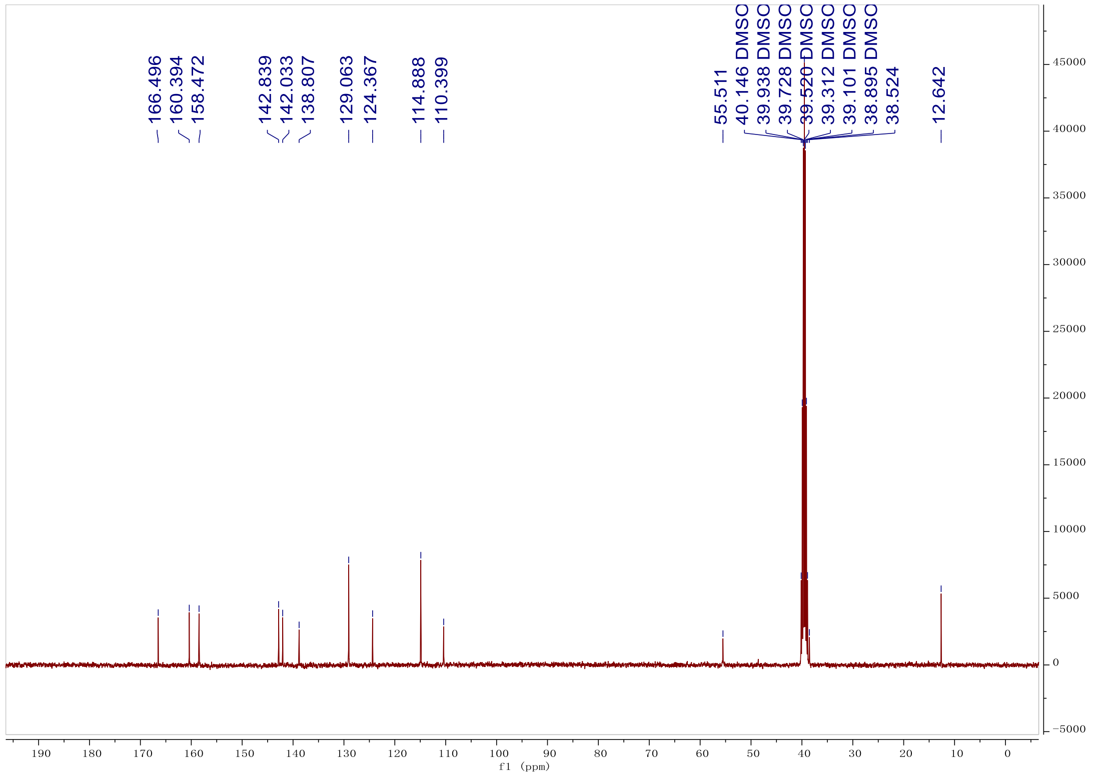

N-(2-(3-hydroxy-2-methyl-4-oxopyridin-1(4*H*)-yl)ethyl)picolinamide (**11a**)

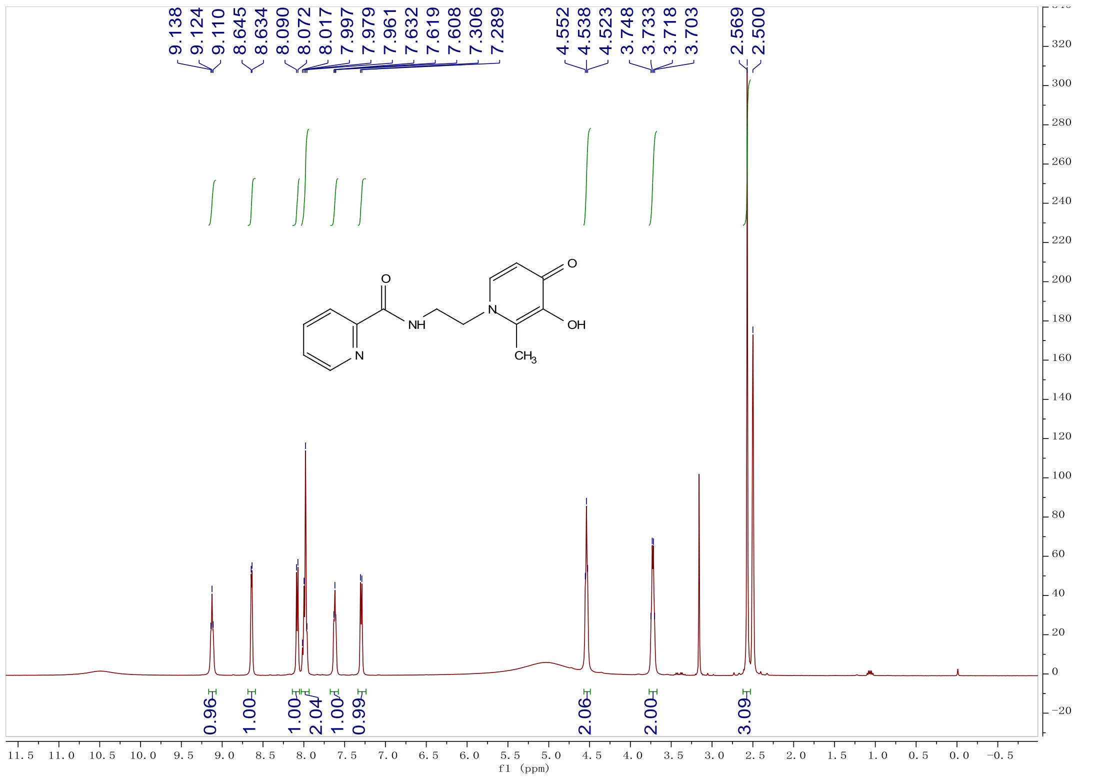

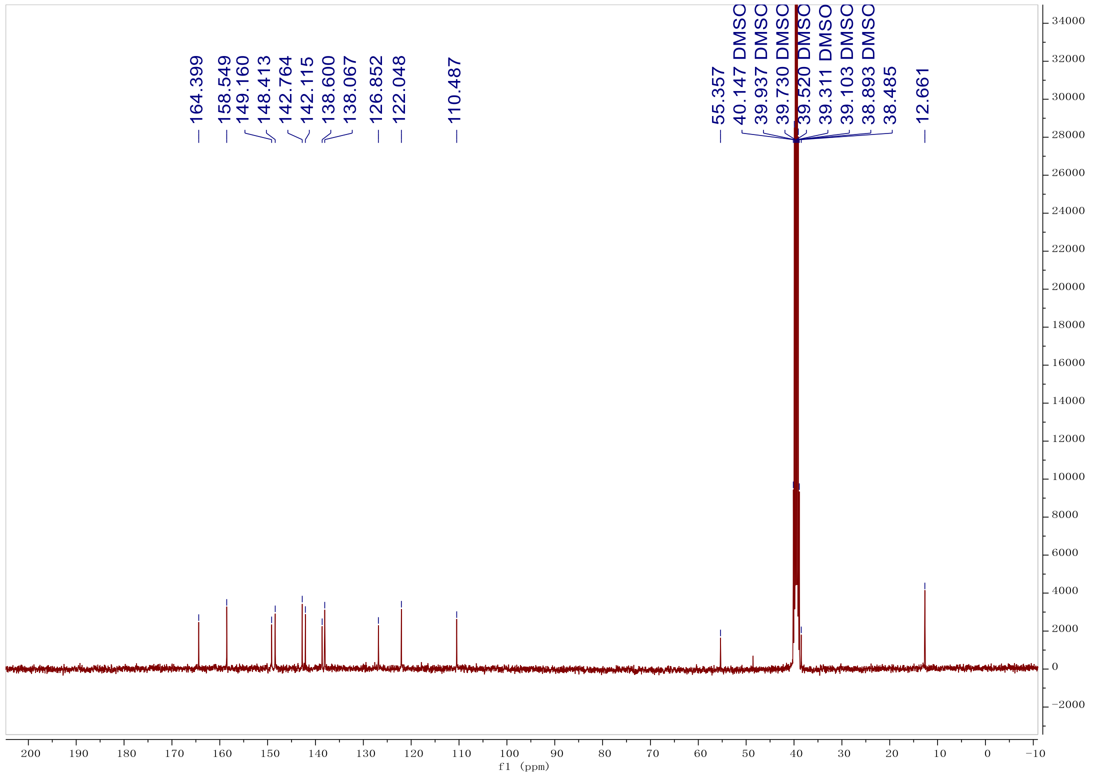

N-(2-(3-hydroxy-2-methyl-4-oxopyridin-1(4*H*)-yl)ethyl)nicotinamide (**11b**)

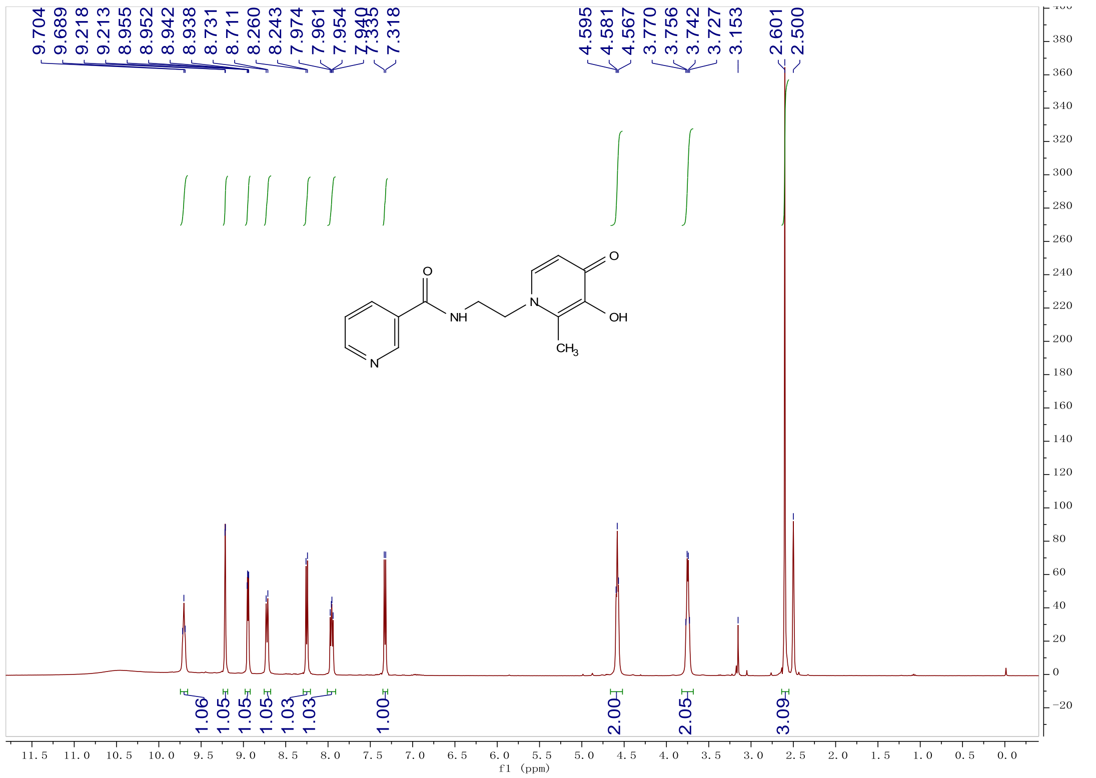

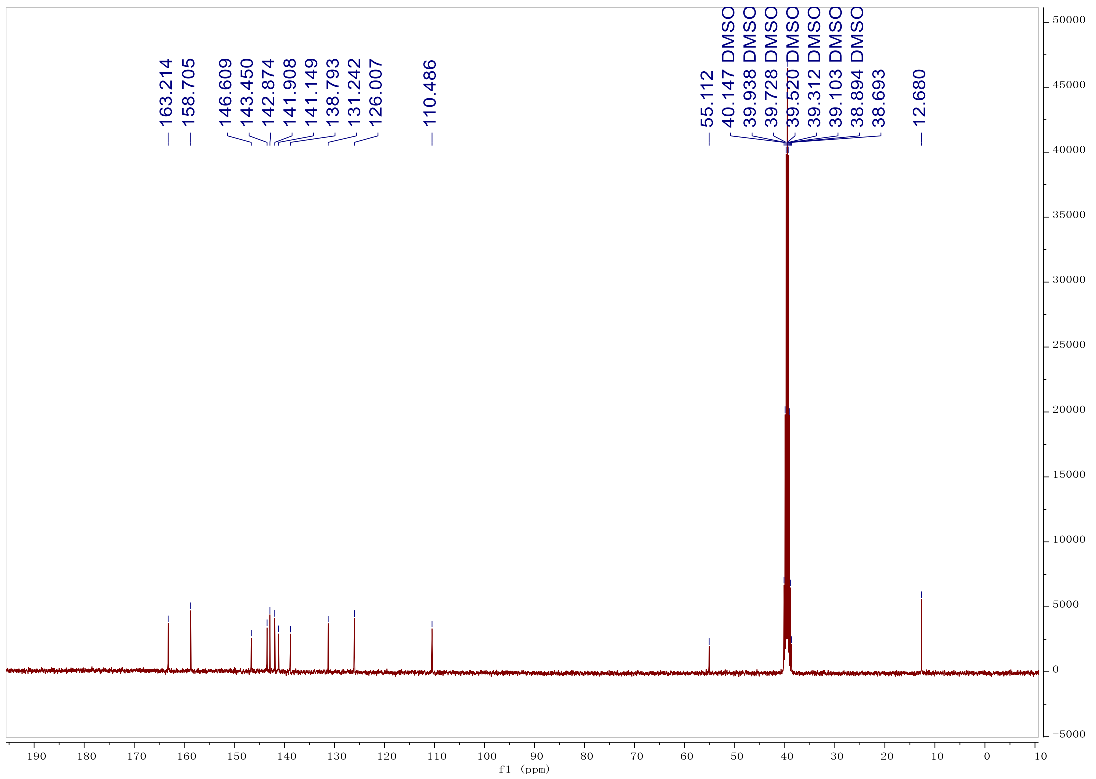

N-(2-(3-hydroxy-2-methyl-4-oxopyridin-1(4*H*)-yl)ethyl)isonicotinamide (**11c**)

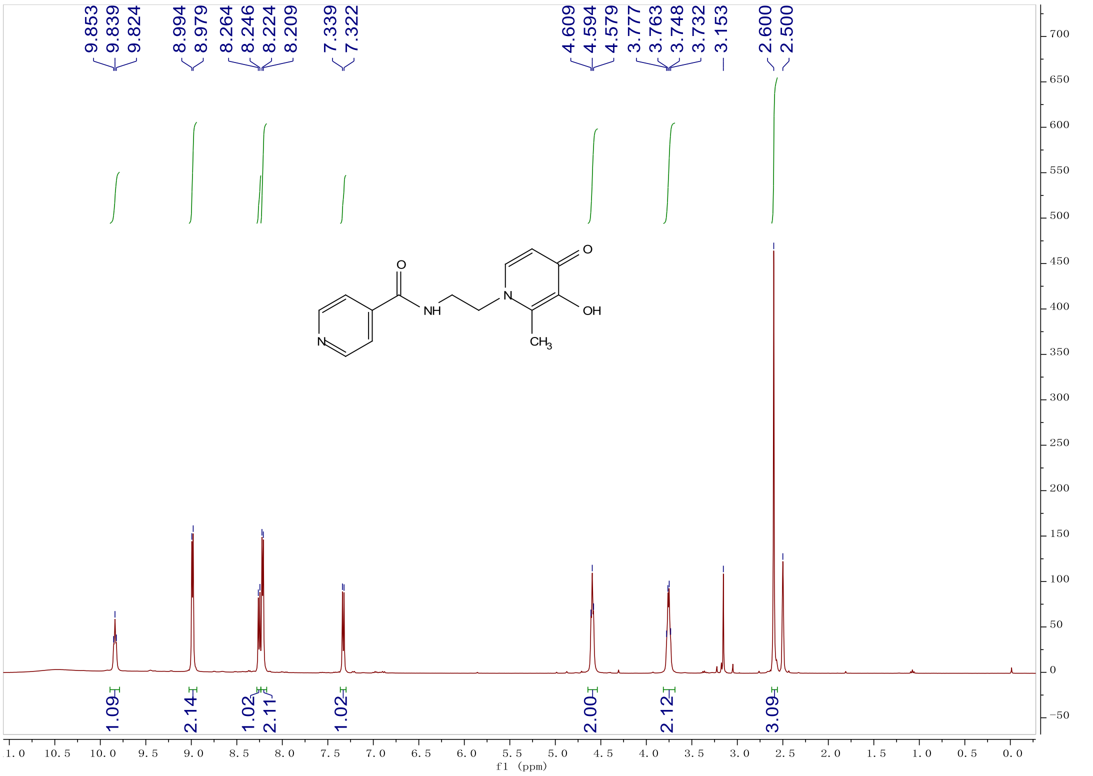

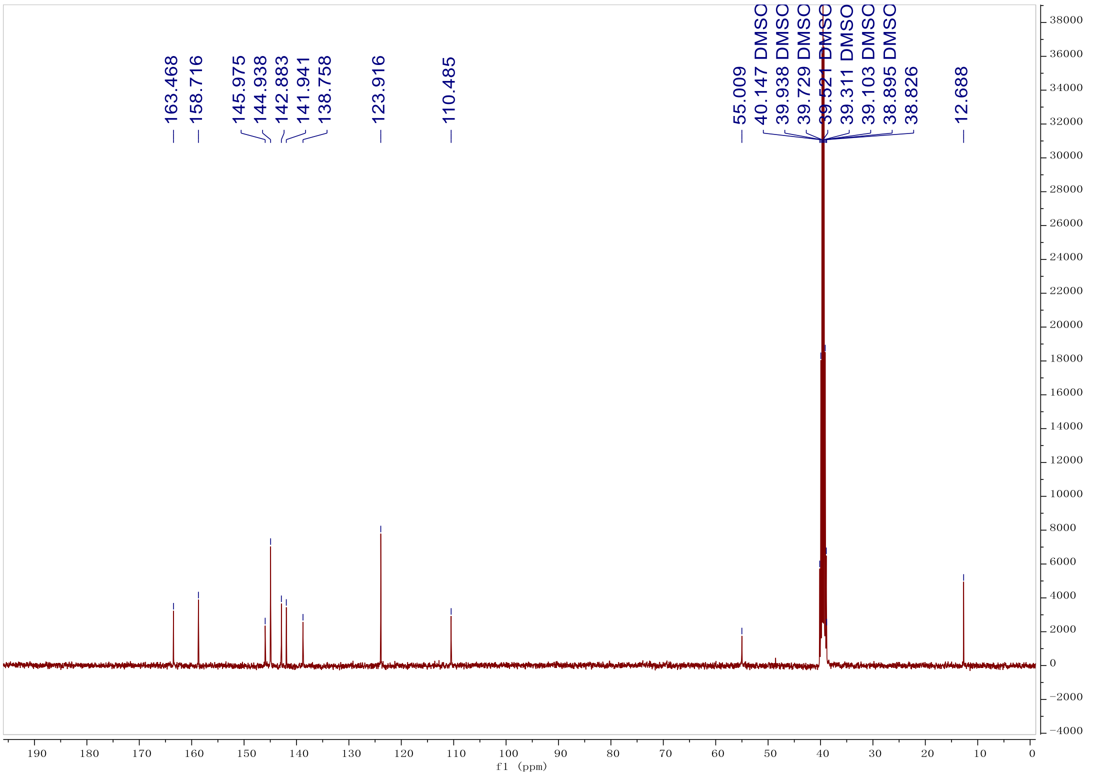

11. References

[1] Baramov, T.; Schmid, B.; Ryu, H.; Jeong, J.; Keijzer, K.; Eckardstein, L.; Baik, M.; D., S. R. How many *O*-donor groups in enterobactin does it take to bind a metal cation? *Chem. Eur. J.* **2019**, *25*, 6955-6962.

[2] Sun, C. Y.; Li, Y. S.; Shi, A. L.; Zhang, J. Z.; Li, Y. F.; Zhao, M. M.; Zhang, L. J.; Zheng, H. C.; Meng, Y.; Ding, H. W.; Song, H. R. Synthesis and evaluation of novel N-3-benzimidazolephenylbisamide derivatives for antiproliferative and Hedgehog pathway inhibitory activity. *Med. Chem. Comm.* **2015**, *6* (6), 1137-1142.

[3] Sit, S. Y.; Conway, C. M.; Xie, K.; Bertekap, R.; Bourin, C.; Burris, K. D. Oxime carbamate--discovery of a series of novel FAAH inhibitors. *Bioorg. Med. Chem. Lett.* **2010**, *20* (3), 1272-1277.

[4] Xu, H. Y.; Wang, X.; Wu, J. C. Preparation and property of a novel soluble oxadiazole-containing functional polyacetylene. *Chin. Chem. Lett.* **2008**, *19* (2), 141-145.

[5] Tang, P.; Dinh, S. Compounds having a cyclic moiety and compositions for delivering active agents. US 8383852 B2. 2013-02-26.

[6] Sun, C. Y.; Zhang, Y.; Wang, H.; Yin, Z. X.; Wu, L. Q.; Huang, Y. M.; Zhang, W. H.; Wang, Y. B.; Hu, Q. B. Design and biological evaluation of phenyl imidazole analogs as hedgehog signaling pathway inhibitors. *Chem. Biol. Drug Des.* **2021**, *97* (3), 546-552.

[7] Xie, Y. Y.; Jiang, X. Y.; Guo, J. N.; Zhang, Y. J. Preparation of formamide pyridinone iron chelator derivatives for anti alzheimer's disease and parkinson's disease drugs. CN 111995567 A. 2020-11-27.

[8] Ducray, P.; Bouvier, J.; Keller, M.; Bergamin, C. Preparation of benzamidoacetonitriles for controlling parasites. WO 2002092552 A2. 2002-11-21.

[9] Jiang, X. Y.; Guo, J. N.; Lv, Y. J.; Yao, C. S.; Zhang, C. J.; Mi, Z. S.; Shi, Y.; Gu, J. P.; Zhou, T.; Bai, R. R.; Xie, Y. Y. Rational design, synthesis and biological evaluation of novel multitargeting anti-AD iron chelators with potent MAO-B inhibitory and antioxidant activity. *Bioorg. Med. Chem.* **2020,** *28* (12), 115550.

[10] Mi, Z. S.; Gan, B.; Yu, S. H.; Guo, J. N.; Zhang, C. J.; Jiang, X. Y.; Zhou, T.; Su, J.; Bai, R. R.; Xie, Y. Y. Dual-target anti-Alzheimer's disease agents with both iron ion chelating and monoamine oxidase-B inhibitory activity. *J. Enzyme Inhib. Med. Chem.* **2019,** *34* (1), 1489-1497.

[11] Gans, P.; Sabatini, A.; Vacca, A. Determination of equilibrium constants from spectrophometric data obtained from solutions of known pH: the program pHab. *Ann. Chim.* **1999,** *89*, 45-49.

[12] Alderighi, L.; Gans, P.; Peters, D.; Sabatini, A.; Ienco, A.; Vacca, A. Hyperquad simulation and speciation (HySS): a utility program for the investigation of equilibria involving soluble and partially soluble species. *Coord. Chem. Rev.* **1999,** *184*, 311-318.

[13] Zhang, C. J.; Yang, K.; Yu, S. H.; Su, J.; Yuan, S. L.; Han, J. X.; Chen, Y.; Gu, J. P.; Zhou, T.; Bai, R. R.; Xie, Y. Y. Design, synthesis and biological evaluation of hydroxypyridinone-coumarin hybrids as multimodal monoamine oxidase B inhibitors and iron chelates against Alzheimer's disease. *Eur. J. Med. Chem.* **2019,** *180*, 367-382.
